# A bioinformatic survey of RNA isoform diversity and expression across 9 GTEx tissues using long-read sequencing data

**DOI:** 10.1186/s12864-025-11919-w

**Published:** 2025-12-05

**Authors:** Madeline L. Page, Bernardo Aguzzoli Heberle, J. Anthony Brandon, Mark E. Wadsworth, Lacey A. Gordon, Kayla A. Nations, David W. Fardo, Mark T. W. Ebbert

**Affiliations:** 1 Sanders-Brown Center on Aging, University of Kentucky, Lexington, KY USA; 2 Division of Biomedical Informatics, Department of Internal Medicine, College of Medicine, University of Kentucky, Lexington, KY USA; 3 Department of Neuroscience, College of Medicine, University of Kentucky, Lexington, KY USA; 4 Department of Biostatistics, College of Public Health, University of Kentucky, Lexington, KY USA

**Keywords:** RNA isoforms, Long-reads, Nanopore sequencing, GTEx

## Abstract

**Background:**

Even though alternative RNA splicing was discovered nearly 50 years ago (1977), we still understand very little about most isoforms arising from a single gene, including in which tissues they are expressed and if their functions differ. Human gene annotations suggest remarkable transcriptional complexity, with approximately 252,798 distinct RNA isoform annotations from 62,710 gene bodies (Ensembl v109; 2023), emphasizing the need to understand their biological effects. For example, 256 gene bodies have ≥ 50 annotated isoforms, and 30 have ≥ 100, where one protein-coding gene (*MAPK10*) even has 192 distinct RNA isoform annotations. Whether such isoform diversity results from biological redundancy or spurious alternative splicing (i.e., noise), or whether individual isoforms have specialized functions (even if subtle) remains a mystery for most genes. Three recent studies demonstrated that long-read RNAseq enables improved RNA isoform quantification for essentially any tissue, cell type, or biological condition (e.g., disease, development, aging, etc.), making it possible to better assess individual isoform expression and function. While each study provided important discoveries related to RNA isoform diversity, deeper exploration is needed.

**Results:**

We sought to quantify and characterize real isoform usage across tissues (compared to annotations). We used long-read RNAseq data from 58 GTEx samples across nine tissues (three brain, two heart, muscle, lung, liver, and cultured fibroblasts) generated by Glinos et al. and found considerable isoform diversity within and across tissues. Cerebellar hemisphere was the most transcriptionally complex tissue (22,522 distinct isoforms; 3,726 unique); liver was the least diverse (12,435 distinct isoforms; 1,039 unique). We highlight gene clusters exhibiting high tissue-specific isoform diversity per tissue (e.g., *TPM1* expresses 19 in heart’s atrial appendage). We also validated 447 of the 700 new isoforms discovered by Aguzzoli-Heberle et al. and found that 88 were expressed in all nine tissues, while 58 were specific to a single tissue.

**Conclusions:**

This study represents a broad bioinformatic survey of the RNA isoform landscape, demonstrating isoform diversity across nine tissues and emphasizes the need for further verification, validation, and functional annotation research to better understand how individual isoforms from a single gene body contribute to human health and disease.

**Supplementary Information:**

The online version contains supplementary material available at 10.1186/s12864-025-11919-w.

## Introduction

According to our analysis of Ensembl gene annotations v109 (released February 2023 [1]), 38% of human gene bodies (85% protein-coding) express multiple RNA and protein isoforms via alternative splicing [2], corroborating other analyses [3, 4]. Yet, despite researchers knowing about alternative splicing for decades [5–7], little is known about individual isoforms for most genes, including if or how their functions differ. In fact, when discussing a given gene’s function, researchers often speak as if the gene has a single function, despite the many RNA and protein isoforms it expresses. We argue that one of the most important next steps in understanding the biology of complex organisms, including human health and disease, will be to understand the functions of individual RNA and protein isoforms arising from a “single” gene (i.e., expressed from a single gene body). While by definition, a gene is simply a specific DNA sequence, if a single gene body gives rise to multiple RNA and protein isoforms with different functions, we question whether it truly is a “single” gene in practice.

Historically, RNA sequencing studies have used short-read sequencing to determine expression across all RNA isoforms for a given gene. Though short-read sequencing can identify splice junctions and has helped make invaluable strides in determining gene and RNA isoform expression, due to technical limitations, it lacks the ability to fully resolve the entire array of isoforms expressed by genes with many exons, inadvertently ignoring the underlying complexity. This approach prevents researchers from: (1) discovering and characterizing all isoforms for a given gene, (2) quantifying expression for distinct isoforms, and (3) fully understanding each isoform’s function, as it is not possible to truly understand an isoform’s function without knowing its expression patterns across all tissues and cell types.

Given the complexity of many eukaryotes, including humans, it is reasonable to assume that distinct RNA and protein isoforms from a single gene body may have unique functions, even if the functions are closely related. There are a few well-documented examples where different isoforms from a single gene body have clearly different (even opposite) functions. Perhaps the most recognized example is *BCL-X* (*BCL2L1*) [8], where one isoform is pro-apoptotic (BCL-Xs) while the other is anti-apoptotic (BCL-XL). Additional examples, where different isoforms appear to have more subtle functional differences, include: (1) *RAP1GDS1* (also known as *SmgGDS*) [9] where its isoforms interact differently with small GTPases [9, 10]; and (2) *TRPM3*, which encodes cation-selective channels in humans, and can be alternatively spliced into two variants targeting different ions [11–13]. *RAP1GDS1* and *TRPM3* showcase isoforms performing functions that are closely related yet not identical, while *BCL-X* (*BCL2L1*) is an excellent example where the isoforms perform entirely opposite functions.

With the advent of long-read sequencing technologies like PacBio and Oxford Nanopore Technologies (ONT), it is now possible to more accurately characterize and quantify expression for individual RNA isoforms expressed for each gene across essentially any tissue, cell type, or biological condition (e.g., diseases, development, aging, etc.). Long-reads are not perfect because of RNA degradation and technical challenges [14], but along with work by Leung et al. [15], our recent work in Aguzzoli-Heberle et al. [3] demonstrated the value of long-read sequencing in human brain and the technology’s ability to quantify expression for individual RNA isoforms, including *de novo* isoforms and entirely new gene bodies. Similarly, Glinos et al. [16] performed long-read RNA sequencing across 15 tissues, providing valuable data to begin assessing expression patterns for individual RNA isoforms across human tissues. By characterizing and quantifying expression for individual RNA isoforms across human tissues, we can begin to understand whether different RNA isoforms from a single gene body perform different functions—even if subtle—where an isoform might be more carefully tuned for the tissue it is expressed in. In fact, some of the subtler differences may explain challenging medical mysteries that remain unresolved, including why patients react differently to certain exposures (e.g., medicines, vaccines, etc.).

Alzheimer’s disease is a prime example of an unsolved medical mystery that could benefit greatly from increased study into RNA isoforms. A number of genes have been implicated in Alzheimer’s disease [17–20], where most—if not all—have multiple annotated RNA isoforms. With the ability to quantify each isoform specifically, we will be able to perform not only differential expression on genes, but on individual RNA isoforms as well. A proof of concept of this was done in our recent work in Aguzzoli-Heberle et al. [3]. Identifying RNA isoforms associated with Alzheimer’s disease would be a large step towards understanding the underlying biology of the disease, determining a pre-symptomatic diagnosis, and discovering and implementing effective treatments.

In their research, Glinos et al. [16] initiated an investigation into tissue-specific RNA isoform expression. Given the broad scope of their article and the crucial importance of this subject, a more thorough exploration as a next step of much downstream research is warranted. To provide a broad bioinformatic survey to characterize the RNA isoform landscape across the human genome and to emphasize the importance of alternative splicing in complex organisms, including humans, we characterize individual isoform expression across nine GTEx tissues (58 samples total) using data generated by Glinos et al. [16], and verify the existence and quantify the expression of new RNA isoforms, including those from new gene bodies, that we previously discovered in Aguzzoli-Heberle et al. [3] across the nine GTEx tissues. We further provide a user-friendly website to explore the RNA isoform expression data for the GTEx samples (https://ebbertlab.com/gtex_rna_isoform_seq.html).

## Results

Here, we present a more focused analysis of RNA isoform expression across nine tissue types, using long-read data generated by Glinos et al. [16] combined with results from our recent study of deep long-read cDNA sequencing in human prefrontal cortex (Brodmann 9/46) in Aguzzoli-Heberle et al. [3]. As isoform discovery has been previously reported for this data both by Glinos et al. [16] originally (71,735 new isoforms discovered; 93,718 isoforms quantified using FLAIR and GENCODE v.26) and by Aguzzoli-Heberle et al. [3] (700 new, high-confidence isoforms discovered; 28,989 isoforms quantified using Bambu and hg38 Ensembl annotation v107), we opted to focus purely on isoform quantification of annotated isoforms and the 700 high-confidence isoforms found in Aguzzoli-Heberle et al. across tissues.

### Methodological overview

**Fig. 1:**
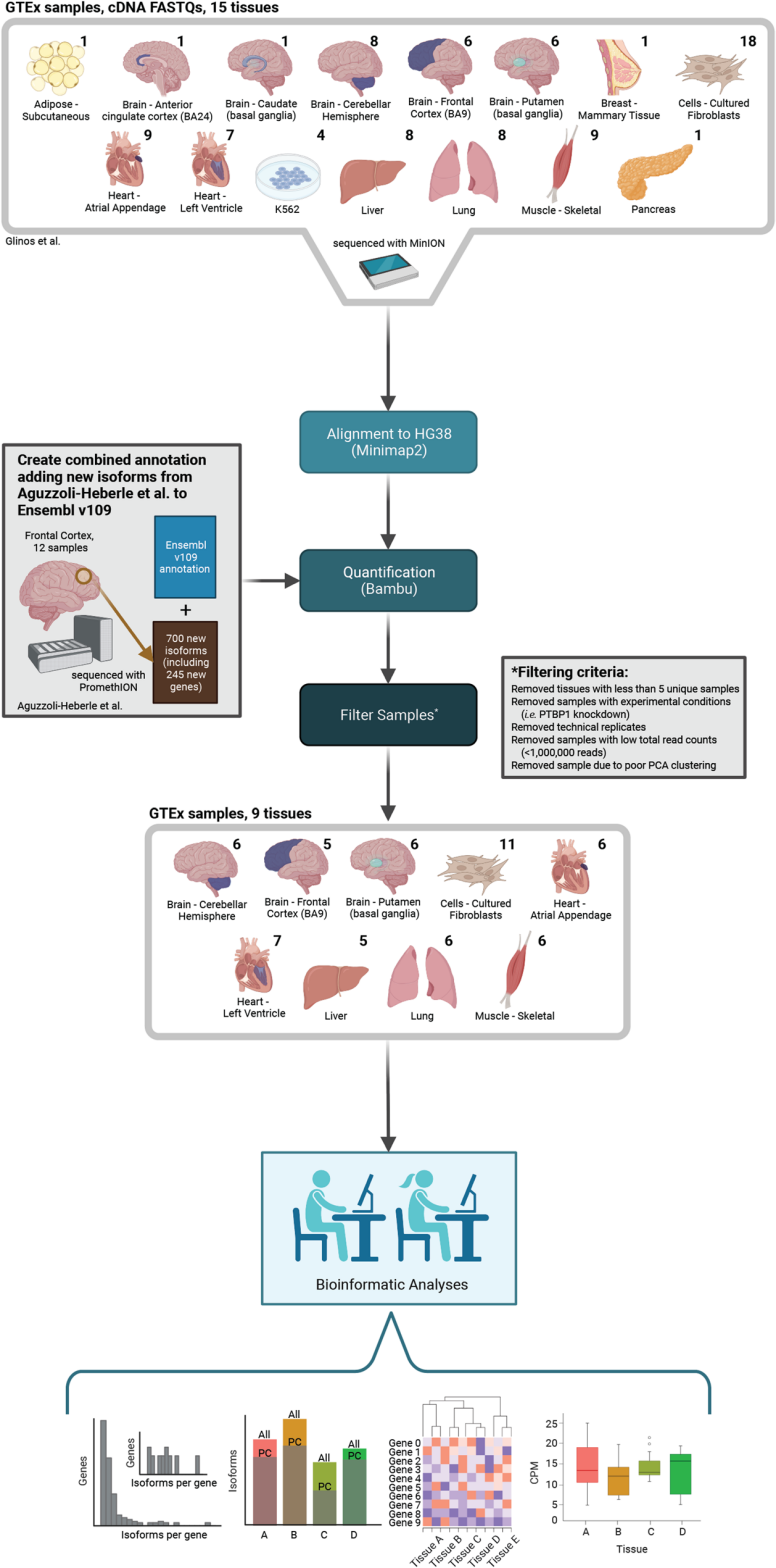
*Study design to assess RNA isoform diversity across human tissues.* We used long-read RNAseq data from GTEx samples generated by Glinos et al. to characterize and quantify RNA isoform expression and diversity across various tissues. These data were combined with results from our recent work in Aguzzoli-Heberle et al. GTEx data were reanalyzed for consistency across data sets, as shown. After filtering samples and tissues (as shown) we included nine tissues in our analyses. The numbers above each tissue indicate the number of samples. Our bioinformatic analyses include determining the expression of isoforms per gene, comparing all isoforms expressed vs. the protein-coding isoforms expressed per tissue, clustering genes expressing at more than five distinct isoforms in at least one tissue, and comparing the expression of an isoform across tissues. Created in BioRender. Ebbert, M. (2024) BioRender.com/t36b121 (Citation for icons)

For this study (see study design in Fig. 1), we retrieved long-read cDNA data for GTEx samples from Glinos et al. [16] sequenced using the Oxford Nanopore Technologies (ONT) minION. For consistency across data sets, we reanalyzed samples using our bioinformatics pipeline. Analysis included isoform quantification with Bambu [21] using Ensembl [1] v109 annotations with the addition of the 700 new isoforms discovered in Aguzzoli-Heberle et al. [3]. We only retained tissues with at least five samples from unique subjects in our analyses. We further removed technical replicates, samples with experimental conditions (i.e., *PTBP1* knockdown), and samples with low total read counts (< 1,000,000 reads; Fig. 1). One liver sample was removed because it clustered poorly during principal component analysis (PCA; Supplemental Figure S1-S2; Supplemental Table S1).

While it is possible to sequence entire RNA isoforms with long-read sequencing, many reads still cannot be uniquely mapped to an isoform because of degradation and technical limitations [14], even with high-quality RNA (e.g., RIN ≥ 9). To address this challenge, Bambu—the quantification tool we employed—provides RNA isoform expression estimates in three forms, each with particular pros and cons: (1) total counts, (2) full-length counts, and (3) unique counts.

The primary advantage of the “total counts” metric is that it utilizes all aligned reads. Credit for a single read count is split for reads that map to multiple isoforms. For example, if a read is equally likely to originate from two isoforms, each isoform receives credit for 0.5 reads. The disadvantage is that this approach likely overestimates expression for some isoforms while underestimating expression for others, but is a valid approach given the challenge of assigning ambiguous reads to the correct isoform.

The “full-length counts” metric only includes reads containing all exon junctions for at least one isoform. If the read is still not unique to a single isoform, Bambu will split the read count among the relevant isoforms (same as total counts). Thus, the full-length counts metric reduces ambiguity, but also likely dramatically underrepresents longer isoforms because they will not be fully sequenced as often [14]. For reference, 51% of reads were “full-length” in this study.

The “unique counts” metric is the strictest and includes only reads that align *uniquely* to a single isoform, because of a unique exon-exon junction structure. In theory, unique counts would be the ideal metric because the alignment is unambiguous and would best represent the observed expression for all isoforms. Unfortunately, this approach is biased against isoforms that only differ towards the 5’ end (degradation bias) and isoforms that have the same exon-exon junctions but only differ in their start or end sites (e.g., shortened 3’ UTR) [21]. Approximately 63% of reads aligned uniquely to an isoform in this study; while far from perfect, this is still a significant improvement over short-read sequencing.

Currently, there is no perfect solution to the challenge of unambiguously assigning reads to the correct isoform. While we anticipate more sophisticated approaches will be developed in the coming years, we used a relatively simplistic approach involving both the total counts (to utilize all reads; i.e., sensitivity) and unique counts (for specificity) provided by Bambu. Specifically, to minimize false positives, a given isoform was only included in this study if it had both a median counts-per-million (CPM) > 1 (total counts) and a median unique counts ≥ 1 across all samples within a given tissue, unless otherwise specified.

### Long-read RNA isoform expression reveals high isoform diversity and varying protein-coding isoform ratios across nine tissues

#### RNA isoform diversity across tissues

In another work, we saw the large number of annotated RNA isoforms in Ensembl v109 (252,798 [2]) and we wanted to characterize and quantify individual isoforms being expressed in human tissues. Using long-read RNAseq data from Glinos et al. [16], we compared RNA isoform expression patterns across nine tissues, including three brain regions (cerebellar hemisphere, frontal cortex, and putamen), two heart regions (left ventricle and atrial appendage), liver, lung, skeletal muscle, and cultured fibroblast cells. We included cultured fibroblast cells for interest since many scientific experiments utilize them.

**Fig. 2:**
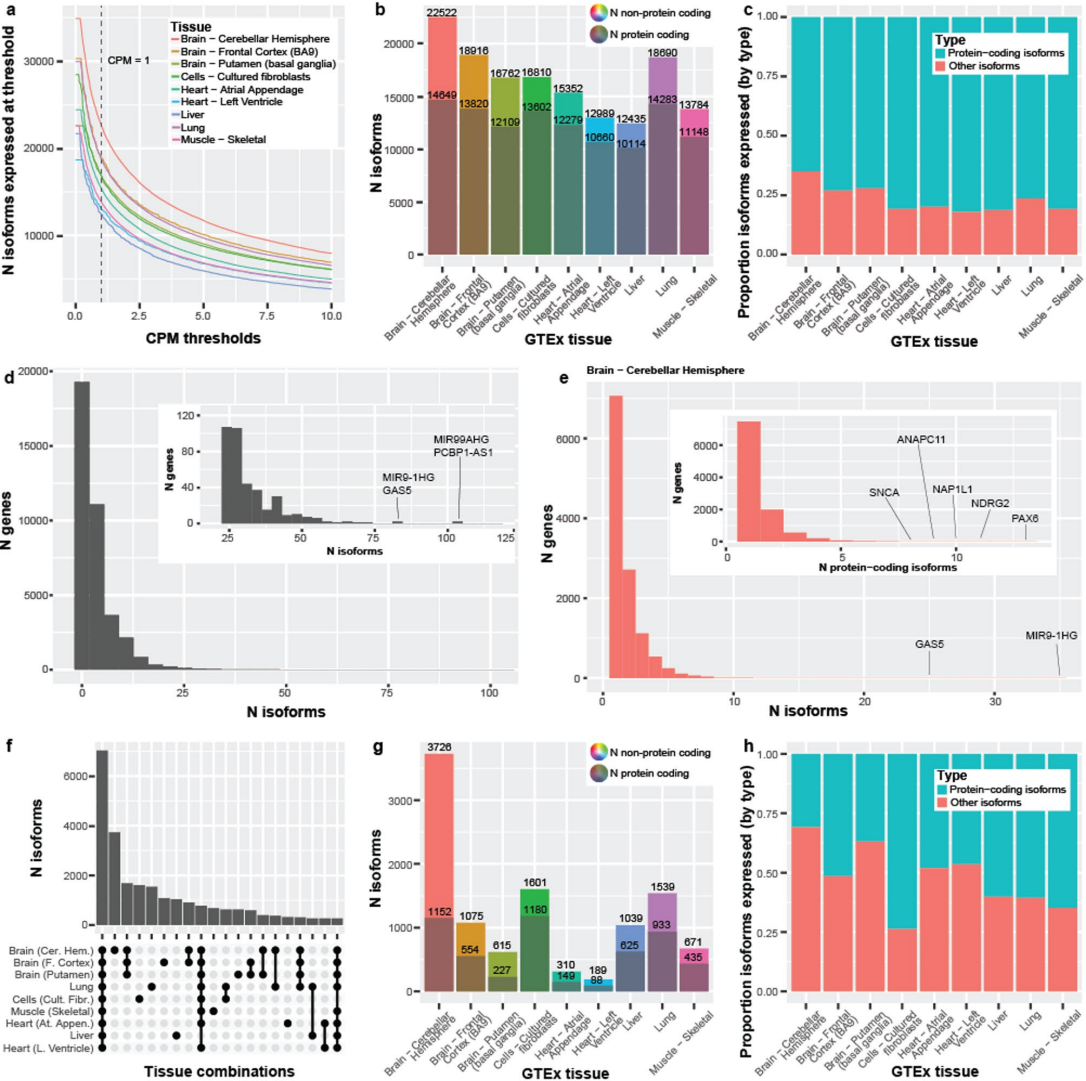
*Long-read RNA isoform expression reveals high isoform diversity and variation across nine GTEx tissues.* **(a)** Isoforms expressed across counts-per-million (CPM) thresholds. Vertical line represents CPM = 1. The flat portion of each distribution reflects the removal of isoforms without median unique counts ≥ 1. **(b)** Isoforms and protein-coding isoforms (shaded) expressed at our standard thresholds (median unique counts ≥ 1 and CPM > 1). Total expressed isoforms differ between tissues. **(c)** Proportion of protein-coding verses other isoforms expressed across the nine tissues. Cerebellar hemisphere expressed the most distinct non-protein-coding RNA isoforms (7,873; 35.0%), while left ventricle of the heart expressed the smallest ratio (17.9%; 2,329 isoforms; Chi-square *p* = 1.90e-255). **(d)** Distinct isoforms expressed per gene, using a threshold of one unique count in any sample (maximum sensitivity). Zoomed subplot shown for convenience. *PCBP1-AS1* expressed the most isoforms (105) for a single gene body; *FANCL* expressed the most (60) for a single protein-coding gene. 88.1% of gene bodies expressing ≥ 10 distinct RNA isoforms were protein-coding, and 1,085 were medically relevant genes including *PSEN2* (19 isoforms), *PAX6* (43), and *MAPK10* (46). Ten gene bodies expressed ≥ 60 distinct RNA isoforms. **(e)** Isoforms per gene and protein-coding isoforms per gene (nested plot) expressed in cerebellar hemisphere using our standard threshold. 25 genes expressed ≥ 10 isoforms and 123 genes expressed ≥ 5 protein-coding isoforms. **(f)** Upset plot showing top 20 interactions of isoform overlap between tissues. 7,023 isoforms were expressed in all nine tissues. Cerebellar hemisphere has the largest number of isoforms uniquely expressed in a single tissue—more than half (53.1%) the number of isoforms expressed across all nine tissues. **(g)** Total number of isoforms and protein-coding isoforms (shaded) uniquely expressed in each tissue. **(h)** Proportion of protein-coding RNA isoforms compared to non-protein-coding, uniquely expressed by tissue. Cerebellar hemisphere expressed 3,726 isoforms uniquely, where only 30.9% (1,152) were protein-coding. 64.8% (435 out of 671) were protein-coding for skeletal muscle.

The number of distinct isoforms that exceeded our thresholds ranged between 12,435 (liver; from 8,857 genes) and 22,522 (cerebellar hemisphere; from 13,236 genes; Fig. 2a, b)—a large range (a difference of 10,087), demonstrating substantial molecular diversity across tissues. For interest, the total number of isoforms with a 0 < CPM ≤ 1 ranged from 5,691 (heart [left ventricle]) to 12,385 (cerebellar hemisphere; Fig 2a), demonstrating the large number of isoforms that were observed with a CPM ≤ 1. Using an ultra-conservative CPM threshold of 10, expressed isoforms ranged between 3,815 (liver) and 7,916 (cerebellar hemisphere; Fig. 2a).

For RNA isoforms annotated as protein-coding (regardless of the gene annotation, i.e. transcript_biotype = protein-coding), the number ranged from 10,114 in liver to 14,649 in cerebellar hemisphere (Supplemental Figure S3, Fig. 2b). Isoforms expressed with a 0 < CPM ≤ 1 ranged from 2,888 (heart [left ventricle]) to 4,723 (cerebellar hemisphere; Supplemental Figure S3). Between 3,521 (liver) and 6,560 (cerebellar hemisphere) protein-coding isoforms were expressed using an ultra-conservative threshold of 10 (Supplemental Figure S3). For interest, the top five expressed transcript biotypes for each tissue closely mirrored the distributions from Ensembl annotations, with some minor exceptions (compare Fig. 1e in Page et al. [2] and Supplemental Figure S4).

Comparing the number of RNA isoforms expressed across tissues, the cerebellar hemisphere not only expressed substantially more total isoforms, but also a substantially larger proportion of non-protein-coding RNAs when compared to heart (Fig. 2b, c). Specifically, cerebellar hemisphere expressed 7,873 (35.0%) non-protein-coding RNA isoforms compared to 2,329 for the heart’s left ventricle (17.9%; *p* = 1.90e-255; Chi-square test; Fig. 2b, c); this includes all RNA isoforms not annotated as “protein-coding”, even if it derived from a known protein-coding gene body. It is unclear whether the significant increase in non-protein-coding RNAs within cerebellar hemisphere compared to an arguably less complex tissue like the heart is biologically meaningful, but it merits further investigation. Determining what role non-coding RNAs play in human health and disease remains a critical question in human biology.

#### RNA isoforms per gene

As discussed in Page et al. [2], Ensembl v109 suggests that 7,255; 256; and 30 genes express ≥ 10; ≥ 50; and ≥ 100 RNA isoforms, where the highest number of annotated isoforms for a single gene body was for *PCBP1-AS1* (long non-coding RNA [lncRNA]; 296) and, for protein-coding, *MAPK10* (192). We were skeptical that such a high number of isoforms could legitimately exist for a single gene. We wanted to determine whether we actually observe genes expressing that many unique isoforms, thus, we quantified the number of isoforms we observed for all genes across the nine tissues included in this study.

To estimate the maximum number of distinct RNA isoforms we observed for each gene body across the nine tissues (erring on the side of sensitivity), we used a minimum threshold of one unique count in any sample for any tissue (i.e., a single read that uniquely aligns to the given isoform for ≥ 1 sample). A large number of annotated isoforms for many of these genes are, in fact, observed at this forgiving threshold (Fig. 2d; Supplemental Table S2). The most RNA isoforms observed for a single gene body and protein-coding gene body were 105 (*PCBP1-AS1*) and 60 (*FANCL*), respectively. Of the gene bodies expressing ≥ 10 distinct RNA isoforms, 2,751 of the 3,121 (88.1%) gene bodies were protein-coding, and 1,085 were medically relevant genes, as defined by Wagner et al. [22], including *PSEN2* (19 isoforms), *PAX6* (43), and *MAPK10* (46; Supplemental Table S2, S3). Similarly, 231 of the 304 (80.0%) gene bodies expressing ≥ 25 distinct RNA isoforms were protein-coding. We only observed ten gene bodies expressing ≥ 60 distinct RNA isoforms, where only one (10%) was protein-coding. Considering that RNA expression measurements are simply a snapshot of expression in time, and the large number of additional tissues not included in this data set, it seems reasonable that some genes may legitimately express the additional RNA isoforms not observed here.

When using a stricter threshold of five unique counts, we observed 1,188 total gene bodies that expressed ≥ 10 distinct RNA isoforms, where 1,036 (87.2%) were protein-coding (Supplemental Table S3; Supplemental Figure S5a); only three gene bodies expressed ≥ 60 RNA isoforms (all lncRNAs). Similarly, when using thresholds of 10 and 20 unique counts (Supplemental Table S3; Supplemental Figure S5b, c), we observed 608 (524 protein-coding; 86.2%) and 270 (227 protein-coding; 84.1%) gene bodies expressing ≥ 10 RNA isoforms, respectively; exactly two and one gene bodies expressed ≥ 60 RNA isoforms, respectively.

Returning to our standard and stricter threshold (median unique count ≥ 1 and CPM > 1), we quantified how many distinct isoforms per gene for a given tissue are consistently expressed. We still consistently observed gene bodies expressing ≥ 10 isoforms and protein-coding gene bodies expressing ≥ 5 isoforms at these high levels (Fig. 2e; Supplemental Figure S6; Supplemental Table S4). Using cerebellar hemisphere as an example (Fig. 2e), 25 genes expressed ≥ 10 isoforms and 123 expressed ≥ 5 protein-coding isoforms. Cerebellar hemisphere expresses more isoforms than other tissues.

#### RNA isoforms per tissue and overlap

Looking at the overall number of expressed isoforms per tissue is itself informative, but understanding the overlap of isoforms expressed across tissues is equally important. Knowing which isoforms are common across all tissues versus those expressed in only a few or even a single tissue provides additional understanding about the potential function of the isoforms. Using our thresholds, 7,023 isoforms had shared expression across all nine tissues (Fig. 2f). Cerebellar hemisphere uniquely expressed 3,726 isoforms—more than half (53.1%) of the number of isoforms expressed across all nine tissues, 2.2x more than the next largest overlap (1,680 isoforms; expressed in all three brain regions), and 19.7x more than the tissue with the least number of uniquely expressed isoforms (189; heart [left ventricle]; Fig. 2f, g). These stark differences mark the cerebellar hemisphere as a truly unique tissue in this study, as similarly suggested by Glinos et al. [16].

Curiously, the proportion of protein-coding isoforms expressed uniquely within a tissue varied. The cerebellar hemisphere uniquely expressed 3,726 isoforms, where only 30.9% (1,152) were protein-coding. Approximately 64.8% (435 out of 671) were protein-coding for skeletal muscle, however (Fig. 2g, h). Cultured fibroblasts had the highest percentage of protein-coding isoforms at 73.7% (1,180 out of 1,601), though whether this would generalize to fibroblasts *in vivo*, we cannot say. Recent work by Cadiz et al. [23], however, demonstrated that microglial cell cultures exhibited significantly unique expression signatures compared to freshly isolated microglia, demonstrating that cell cultures likely do not accurately represent reality. The large range in ratio of protein-coding isoforms to all other RNA isoforms solidifies our stance that understanding the interaction of isoforms in different tissues is needed.

#### Genes selectively express many isoforms per tissue

We were intrigued to find certain genes actively transcribe many distinct isoforms in a single tissue type, which raises the question of why a single gene body is expressing multiple distinct isoforms simultaneously. Ultimately, targeted experimental work will be required to determine what function, if any, each isoform from a single gene body performs, but characterizing which genes and tissues these events occur in is the first step to understanding this phenomenon.

**Fig. 3:**
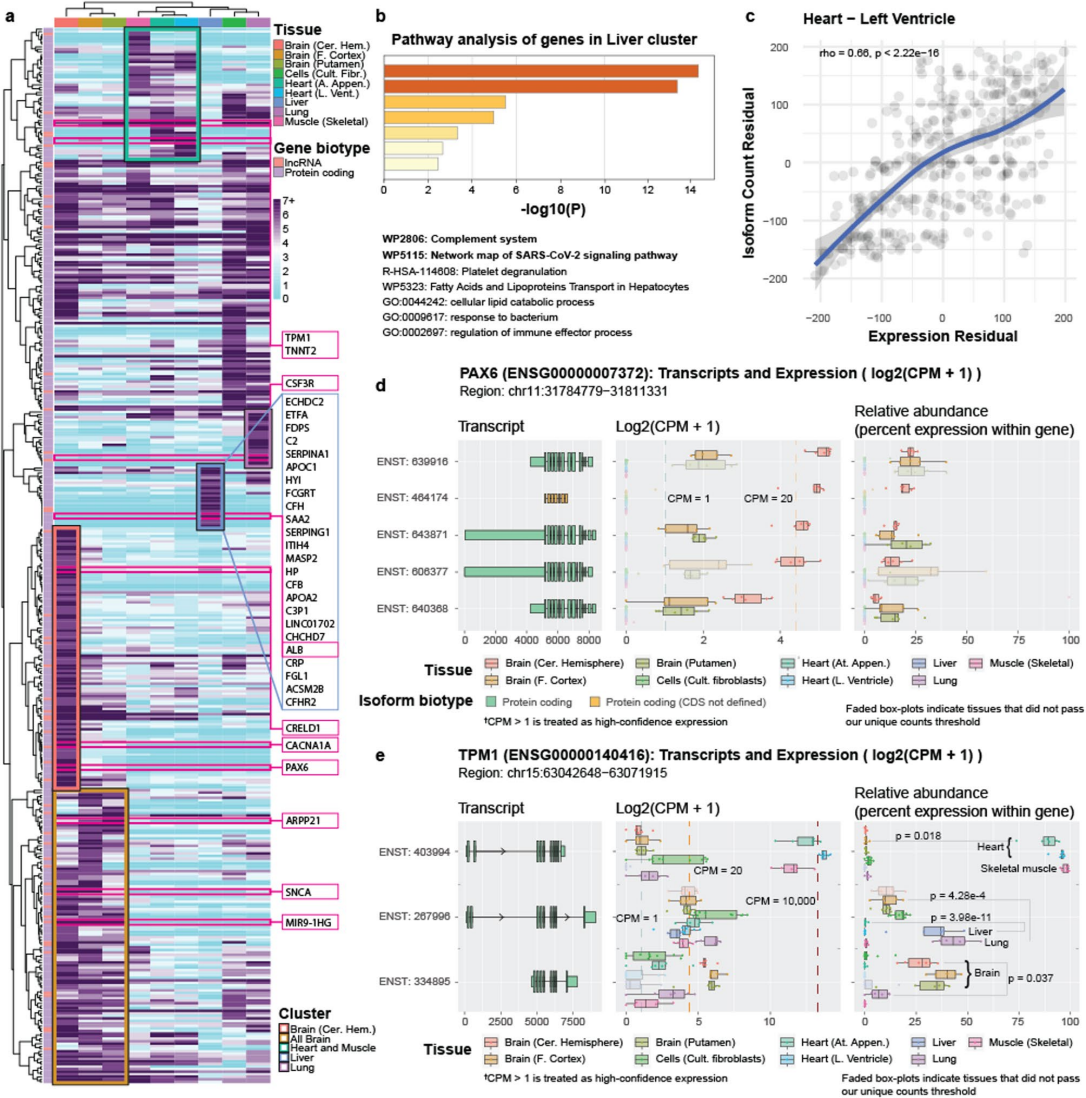
*Genes selectively express many isoforms per tissue.* We selected all genes expressing > 5 isoforms in ≥ 1 tissue. **(a)** Clustered heatmap showing isoform diversity (i.e., number of isoforms, not expression values) to identify gene clusters that preferentially express many isoforms in specific tissues. Dark purple indicates ≥ 7 distinct isoforms. Each tissue set had a representative cluster. One gene (*MIR9-1HG*) expressed 36 isoforms in putamen. **(b)** Metascape pathway analysis for liver cluster shows enrichment for genes associated with immune response, including the complement system and SARS-CoV-2 signaling pathway. **(c)** Gene expression residuals and isoform count residuals (controlling for gene length) were correlated; left ventricle shown as representative sample (Spearman’s; rho = 0.66 and *p* < 2.22e-16; see Supplemental Figure S37 for other tissues). We cannot determine cause and effect from these data alone, however, but can only conclude the two are correlated. Figures **(d-e)** show two representative gene examples from our R Shiny app (https://ebbertlab.com/gtex_rna_isoform_seq.html) expressing many isoforms within a single tissue, but with distinct expression patterns. For both genes (*PAX6* & *TPM1*), it is unclear whether overall gene expression is driving the high number of expressed isoforms or if the need for multiple isoforms causes a higher gene expression, but demonstrates the need to determine whether individual isoforms are biologically functional. **(d)** *PAX6* expressed 16 isoforms in cerebellar hemisphere; we highlight the top five, here (see full plot in Supplemental Figure S17). **(e)** *TPM1* expressed 16 and 19 isoforms in left ventricle and atrial appendage, respectively. We highlight three *TPM1* isoforms showing preferential isoform expression across distinct tissue sets (see full plot in Supplemental Figure S27). For those isoforms, we included adjusted p-values for t-tests performed by IsoformSwitchAnalyzeR indicating the differential isoform usage between tissues. See Supplemental Table S6 for all values.

We selected all genes expressing > 5 distinct isoforms above our thresholds in at least one tissue, totaling 416 genes. We generated a clustered heatmap based on the isoform diversity (i.e., the number of isoforms, not expression values) to identify gene clusters that preferentially express many isoforms for specific tissues (Fig. 3a; Supplemental Figure S7; Supplemental Table S5). We identified several tissue-specific clusters, including brain (as a whole), cerebellar hemisphere, muscle (including heart and skeletal muscle), liver, and lung (Fig. 3a; Supplemental Figure S7; Supplemental Table S5). There are several fascinating examples of isoform complexity for each cluster, but we will only highlight a few. We additionally ran these highlighted genes through the R package IsoformSwitchAnalyseR [24] and related external tools (CPAT3 [25], Pfam [26], IUPred2A [27], SignalP6 [28], DeepLoc2 [29], and DeepTMHMM [30]) to assess predicted protein domains as well as differential isoform usage. Output from the external tools was fed back into IsoformSwitchAnalyseR for differential isoform usage and plotting. For ease in writing, when referring to outputs from any of these, we will only refer to IsoformSwitchAnalyseR. Our intent in this section is to showcase the breadth of genes and fields of study affected by high isoform diversity.

#### *ARPP21*, *SNCA*, and *MIR9-1HG* exhibit high isoform diversity in brain

An interesting example of RNA isoform diversity across all three brain regions, compared to the other tissues, is *ARPP21*. Though little is known about this gene, *ARPP21* is highly expressed across brain regions and has been associated with entorhinal cortex thickness in an Alzheimer’s disease study [31]. It also displays high isoform diversity across all three brain regions included in this study, ranging from four (cerebellar hemisphere) to nine (putamen; Fig. 3a; Supplemental Figure S8; Supplemental Table S5). Interestingly, the only other tissue within this study where any *ARPP21* isoform exceeds our threshold is skeletal muscle, which raises another critical point about gene and isoform function: given the distinct differences between the two tissues, what common molecular function fulfilled by *ARPP21* is needed by both brain and skeletal muscle but not other tissues (Fig. 3a; Supplemental Figure S8; Supplemental Table S5). Indeed, according to GTEx, relative expression of *ARPP21* is highest in brain, followed by skeletal muscle, where most other tissues have very low expression [32] (Supplemental Figure S9). Looking at the IsoformSwitchAnalyzeR transcript plot, the two isoforms with the longer coding regions have additional protein domains compared with the other five isoforms, which appear to only differ from each other in their untranslated regions (UTR; Supplemental Figure S10). Interestingly, the longer isoforms have lower isoform usage in putamen compared to the other brain regions and skeletal muscle, though the overall expression of the isoforms is still high (ENST00000684406: frontal cortex to putamen *padj* = 4.52e-7; cerebellar hemisphere to putamen *padj* = 1.12e-12; putamen to skeletal muscle *padj* = 2.08e-10; Supplemental Table S6).

*SNCA*, acknowledged for its role in both Parkinson’s [33] and Alzheimer’s disease [33–36], is also highly expressed across all included brain regions and has elevated isoform diversity (putamen: 4; frontal cortex: 8; cerebellar hemisphere: 8) compared to other tissues that express this gene (lung: 2; heart [left ventricle]: 1; heart [atrial appendage]: 1; Fig. 3a; Supplemental Figure S11; Supplemental Table S5). The transcript ENST00000394991 is expressed across all tissues that express *SNCA* and accounts for more than half of the total gene expression, but its expression is at least 5.5x higher in the three brain regions compared to other tissues, with median CPMs > 50 for all three regions. This isoform shares identical protein-coding sequence with four other isoforms expressed in this data (differing only in theirUTRs; three seen in the IsoformSwitchAnalyzeR transcript plot Supplemental Figure S12), all of which have much lower CPM values (1.42 to 38.87 in brain). Why ENST00000394991 is clearly favored over the other isoforms when the only differences are in the UTR, and whether the UTR differences are biologically meaningful remain important biological questions that could have huge implications on our understanding of diseases, such as Alzheimer’s disease. Note that the most highly expressed isoform in GTEx short-read data is ENST00000508895, but the trend of more isoforms being expressed, and higher expression in the brain, holds (Supplemental Figure S13).

As a non-protein-coding example, we observed a long non-coding RNA (lncRNA), *MIR9-1HG* (also known as *C1orf61*) that is only expressed in brain regions and expressed between 32 and 36 distinct isoforms (Fig. 3a; Supplemental Figure S14, S15). While little is generally known about non-protein-coding gene bodies, they constitute 68% of all annotated gene bodies in Ensembl v109 [1, 2]. *MIR9-1HG*, specifically, has been implicated in development of ganglionic eminences, according to Zhao et al. [37], which is consistent with *MIR9-1HG* being so highly expressed in brain tissue. Two isoforms were predicted from the IsoformSwitchAnalyzeR output to be protein-coding (Supplemental Figure S16). Why *MIR9-1HG* expresses > 30 distinct isoforms and whether they have distinct functions (or any function at all) is an important question.

#### PAX6, CACNA1A, and CRELD1 exhibit high isoform diversity in cerebellar hemisphere

Throughout this work, we have highlighted that the cerebellar hemisphere has greater isoform diversity than other tissues, including other brain regions. Thus, it is not surprising that cerebellar hemisphere has its own cluster that is distinct from the other brain regions (Fig. 3a; Supplemental Figure S7). Two examples of isoform diversity within the cerebellar hemisphere are *PAX6* and *CACNA1A*, where only the three brain regions have any isoforms above our noise threshold. Only two to three isoforms met our criteria for putamen and prefrontal cortex for both genes, yet 16 and 11 isoforms met our criteria for the cerebellar hemisphere for *PAX6* and *CACNA1A*, respectively (Fig. 3a; Supplemental Figure S7, S17, S18; Supplemental Table S5). Similar patterns hold for GTEx short-read data (Supplemental Figure S19, S20). Notably, *PAX6* is historically associated with eye diseases [38–40] and early neural development [41], yet it is already known to be highly expressed in the cerebellar hemisphere in adults [42], suggesting a major function in this tissue. The isoform ENST000004664174, labeled as “protein-coding CDS not defined” is predicted from the IsoformSwitchAnalyzeR output to be “nonsense mediated decay sensitive” and is not expressed in any other tissues (Supplemental Figure S21). *CACNA1A*, on the other hand, is known to be directly involved in spinocerebellar ataxia type 6 (SCA6) [43], where several DNA variants directly cause the disease. The top four protein-coding isoforms did not meet our unique counts threshold, though IsoformSwitchAnalyzeR shows that they have many predicted domains (Supplemental Figure S22) and expression of at least one of the isoforms is likely high in brain samples. Knowing the individual functions of individual RNA isoforms could be essential to treating this disease.

Similarly, the number of unique *CRELD1* isoforms ranges from three to five for all tissues except for the cerebellar hemisphere, where 12 exceeded our noise threshold (Fig. 3a; Supplemental Figure S23; Supplemental Table S5)—double that of the next highest (liver). *CRELD1* is a known heart disease gene [44, 45], yet it has recently been implicated in a range of neurodevelopmental disorders, including epilepsy and movement disorders, for which the cerebellar hemisphere is a major player [46]. A foundation dedicated to *CRELD1*-related neurodevelopmental diseases and associated research (CRELD1 Warriors; https://www.creld1.com/) has even been established in recent years [47]. Notably, total *CRELD1* expression is approximately 3x higher in cerebellar hemisphere than in heart, according to GTEx [48] (Supplemental Figure S24), and its isoform diversity in cerebellar hemisphere is 4x greater (twelve) than what we observed in heart (three). The stark difference in isoform diversity between tissue regions of the same primary organ (e.g., brain) and the differences in coding regions and predicted protein domains (Supplemental Figure S25) of these isoforms are excellent examples demonstrating the need to understand why a single gene body can express many unique isoforms.

#### *TPM1* and *TNNT2* exhibit high isoform diversity in heart and skeletal muscle

According to the GTEx portal [49], *TPM1* has high expression across several tissues, including heart and skeletal muscle, and plays an important role in cellular structure across numerous cell and tissue types (Supplemental Figure S26). In muscle cells, specifically, *TPM1* provides structural support to the actin filament [50]. Most of the nine tissues expressed between two and nine isoforms (Fig. 3a; Supplemental Table S5), but 16 and 19 RNA isoforms were expressed in the heart’s left ventricle and atrial appendage, respectively, demonstrating *TPM1*’s heart-specific isoform diversity. GTEx short-read data shows additional tissues that have high *TPM1* isoform diversity (Supplemental Figure S26). Total gene expression in GTEx long-read data was a median CPM of 14,355.69 and 8,890.88, respectively, including isoforms that did not exceed our noise threshold (Fig. 3a; Supplemental Figure S27; Supplemental Table S5). Those expression values are roughly 1,344x and 859x greater than the total median expression value in each tissue, respectively. The tissue expressing the next highest number of distinct isoforms was cultured fibroblasts with eleven, but whether this pattern would generalize to fibroblasts *in vivo* is unknown.

The *TNNT2* gene is directly associated with various cardiomyopathies [51–53] and is primarily expressed in heart muscle [54] (Supplemental Figure S28). Like *TPM1*, *TNNT2* expresses a large repertoire of RNA isoforms (16), but six are annotated as non-protein-coding (Fig. 3a; Supplemental Figure S29; Supplemental Table S5)—again raising the question regarding what role each of the individual isoforms play in heart health. The complexities of understanding how genes interact throughout tissues are staggering. Adding the complexity of isoform diversity is even more difficult to comprehend. Interestingly, five *TNNT2* isoforms were expressed in prefrontal cortex and one in lung, but those are expressed at much lower levels (median CPMs ranged from 1.39 to 29.5) than what we observed for *TNNT2* in heart (median CPMs ranged from 1.01 to 3,494.74 for the 16 isoforms; Fig. 3a; Supplemental Table S5). It is interesting that the most highly expressed isoform in frontal cortex is not the isoform that is most highly expressed in heart, and the two isoforms (ENST00000438742 and ENST00000422165) differ in exons and UTR (Supplemental Figure S30). Whether the relatively lowly expressed isoforms in prefrontal cortex and lung are performing important biological functions in these tissues remains unknown.

#### Albumin (*ALB*) isoform diversity in liver may explain its many known functions

The albumin protein (from the gene *ALB*) was allegedly one of the first proteins discovered, and one of the most studied in history, where it was first precipitated from urine circa 1500 A.D [55]. The breadth of albumin’s functions is remarkable. The albumin protein is expressed and excreted by the liver and is widely reported to be the most abundant protein in blood plasma [55–58]. Albumin is a multifunctional protein that contributes approximately 70% of osmotic pressure [56, 59] while also serving to bind and transport a range of endogenous and exogenous compounds, including reactive oxygen species (ROS), pharmaceuticals, and hormones [55–59]. We observed eight *ALB* isoforms expressed in the liver (Fig. 3a; Supplemental Figure S31, S32; Supplemental Table S5), seven of which are annotated as protein-coding with distinct CDS regions. No other tissue expresses a single *ALB* isoform passing our thresholds. The most highly expressed isoform is ENST00000295897 (median CPM: 31,327.14), followed by ENST00000415165 (median CPM: 7,896.24). For reference, the median CPM for all isoforms expressed in the liver is 4.51, even when excluding isoforms that did not reach our noise threshold. Given the many functions attributed to *ALB*, it seems plausible that the multiple isoforms afforded by alternative splicing make this possible. The IsoformSwitchAnalyzeR output plot shows that there is a difference in the number of Serum_albumin domains in these two isoforms (Supplemental Figure S33). Interrogating these isoforms further would allow us to determine what the differences are in the protein products and if the proteins have different or complementary functions.

Using the 24 genes in the liver cluster (Fig. 3a), we performed a pathway enrichment analysis using Metascape [60] and found enrichment for immune response, including the “complement system” and “network map of SARS-CoV-2 signaling pathways” (Fig. 3b).

#### CSF3R isoform diversity in lung highlights lung’s role in immunity

A part of the lung cluster, *CSF3R* is a gene that encodes a cytokine receptor and is involved in the creation and regulation of granulocytes, a type of white blood cell. *CSF3R* has been directly implicated as a causal gene for chronic neutrophilic leukemia and atypical chronic myloid leukemia [61, 62], and has also been associated with neutropenia (insufficient neutrophils) [63]. In all, *CSF3R* is strongly implicated as an immune-related gene, and neutrophils specifically, yet we observed seven different isoforms above our thresholds in the lung, while other tissues expressed zero or one isoforms (Fig. 3a; Supplemental Figure S34, S35). Observing a neutrophil-related gene expressing seven distinct isoforms in the lungs may seem counterintuitive initially, but the lungs host a large number of both innate and adaptive immune cells to protect against the regular exposure to pathogens [64, 65]. Of the seven observed isoforms, three are annotated as protein-coding, three are annotated as retained intron, and one is protein-coding without a defined coding sequence (CDS). For *CSF3R*, the differences between protein-coding isoforms appear to be minimal compared to some of the other genes we have highlighted, raising the question of what the biological importance of each isoform is. Output from IsoformSwitchAnalyzeR predicts one of the retained intron isoforms as protein-coding with a very different coding region compared to the others (Supplemental Figure S36). Larger studies, deeper analyses, and, ultimately, experimental work will be required to determine the functional changes and importance of these many isoforms.

#### A word of caution: total gene expression and number of expressed isoforms are correlated

While we have highlighted gene clusters that express an enriched number of isoforms in specific tissues, it is important to exercise caution when drawing conclusions. In biology, proper interpretation is often not simple. Many reasons and causes can be identified as the potential answer, but without proper experiments and validation, we cannot be sure of their accuracy. There are likely additional underlying reasons for these genes to express multiple isoforms in a single tissue and not in others. Understanding the biological purpose in isoform diversity is, indeed, complex.

In an attempt to assess whether increased isoform diversity could result from increased gene expression, we tested for a correlation between the two measures using Spearman correlation and controlling for gene length. We found that total gene expression is, in fact, correlated with the number of distinct isoforms expressed (rho = 0.66, *p* < 2.22e-16 for heart [left ventricle]; Fig. 3c, Supplemental Figure S37). This correlation, however, does not infer cause (i.e., whether increased gene expression drives isoform diversity or vice-versa). Additional experiments are needed, as the answer surely depends on the gene in question.

One potential reason for more isoform diversity in one tissue over another is that the increased number of isoforms exceeding our threshold results from an overall increased gene expression and potentially spurious alternative splicing. Even though a given gene body may be expressing ten discrete isoforms, often most of the expression can come from one or two specific isoforms. Still, some of the isoforms with lower expression relative to other isoforms from the same gene body are expressed at levels much higher than most genes in the sample as a whole. On the other hand, overall gene expression would also increase if a given cell expresses multiple distinct isoforms to achieve multiple distinct biological functions. Thus, we cannot infer cause or biological significance from these data alone but can only conclude that the two metrics are correlated. We think it is likely that some isoform diversity is caused by spurious splicing events, but that much is likely functional. Specifically, it has long been established that alternative splicing is an evolved process that enables biological diversity and complexity [66–68] and it seems unlikely to us that only 20,000+ protein-coding genes performing a single function could support organisms as complex as humans with such diverse cell types, tissues, and developmental stages. Regardless of whether the distinct isoforms expressed from a single gene perform distinct functions (even if subtle), we need to fully characterize their nature to truly understand the underlying biology of human health and disease.

Here, we provide two examples (*PAX6* & *TPM1*) with different expression patterns, where it is unclear if total gene expression is driving the number of isoforms or if the number of isoforms is driving total gene expression. As discussed, *PAX6* expresses 16 isoforms above our thresholds in cerebellar hemisphere (Supplemental Figure S17). Twelve of the 16 isoforms represent only minimal relative expression, ranging from 0.71% to 4.96%, the biological significance of which is unclear. The other four, however, have high expression (CPM between 19.99 and 35.67) and arguably similar relative expression levels (ranging from 12.69% to 22.22%; Fig. 3d). Given the top four isoforms constitute 68.74% of total *PAX6* expression with similar relative abundances, it could indicate the four isoforms are specifically necessary for optimal cellular function. In other words, based on these data alone, we cannot draw clear conclusions about the biological necessity of the twelve lowest-expressed isoforms within cerebellar hemisphere, nor can we determine the biological function of the top four isoforms. We can say, however, that at least four isoforms are actively expressed, and thus deeper work is needed to formally assess their function.

*TPM1* also appears to raise the same question (is overall gene expression driving isoform expression or vice versa) but based on a different expression pattern compared to *PAX6*. As discussed, total *TPM1* expression levels in the heart’s left ventricle and atrial appendage are extremely high, being 1,344x and 859x greater than the median expression value in the respective tissues (10.68 and 10.35). Thus, given such high expression, it is plausible that many of the 16 and 19 expressed isoforms may have met our inclusion criteria due to potential spurious splicing. Additionally, a single isoform (ENST00000403994) constitutes between 89.2% and 98.2% of *TPM1*’s total expression for skeletal muscle and the two heart tissues (next highest tissue at 2.0%; Fig. 3e; Supplemental Figure S27).

The evidence supporting at least some of the additional *TPM1* isoforms as biologically functional, however, is that three isoforms have tissue-specific expression, where ENST00000403994, ENST00000267996, and ENST00000334895 are preferentially expressed in heart and skeletal muscle, lung and liver (percent abundance: 37.1% to 43.0%; next highest tissue at 18.3%; Fig. 3e; Supplemental Table S6), and brain tissues (percent abundance: 28.1% to 40.1%; next highest tissue at 6.8%; Fig. 3e; Supplemental Table S6), respectively. Interestingly, the coding regions for these three isoforms are different, and though all three contain the same types of protein domains according to IsoformSwitchAnalyzeR, their predicted domain organizations are different (Supplemental Figure S38). Whether these distinct isoforms are ultimately functionally different remains to be seen. In either case, the examples of *PAX6* and *TPM1* highlight the need to exercise caution when interpreting results, as individual metrics can be misleading.

### Newly discovered isoforms from brain frontal cortex are expressed across various tissues

In our recent work by Aguzzoli-Heberle et al. [3], we discovered a total of 700 new, high-confidence RNA isoforms using deep long-read cDNA sequencing in human prefrontal cortex (Brodmann area 9/46) from twelve aged brain samples (six Alzheimer’s disease cases and six age-matched controls), where 428 of the 700 new isoforms were from known nuclear gene bodies, 267 were from entirely new nuclear gene bodies, and five were spliced isoforms from mitochondrially encoded gene bodies. We directly validated many of these new RNA isoforms from known genes at the protein level via mass spectrometry, along with three from new gene bodies [3]. Observing spliced isoforms from mitochondrially encoded gene bodies was entirely unexpected, given dogma dictates that mitochondrially encoded genes are not spliced, but our results supported previous work by Herai et al. [69], demonstrating the same phenomenon. For interest, we also discovered 2,729 other potential new RNA isoforms [3] but here, we limit our analyses to those we considered high-confidence (i.e., median CPM > 1 in our previous study). Thus, here, we sought to quantify expression for our high-confidence isoforms across the nine GTEx tissues.

**Fig. 4:**
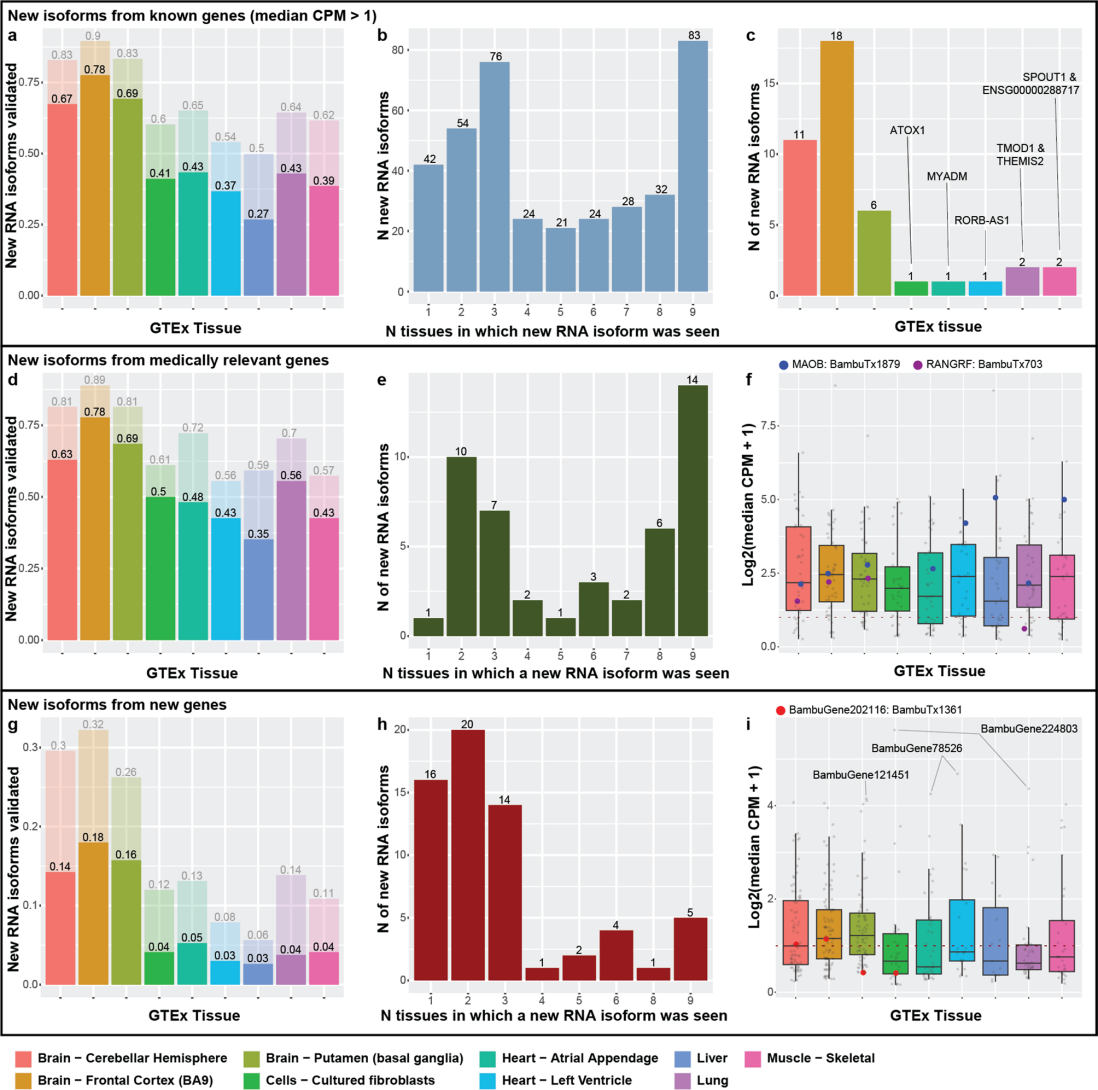
*Newly discovered isoforms validated across nine GTEx tissues, including many from new gene bodies.* **(a-c)** deal with new isoforms from previously known gene bodies. **(a)** Proportion of new isoforms from known genes that validated (at median unique counts ≥ 1 and median CPM > 1) for each tissue. 292 (67%), 336 (78%), and 300 (69%) of our newly discovered RNA isoforms from known gene bodies were expressed in the cerebellar hemisphere, frontal cortex, and putamen, respectively, within the GTEx data. New isoforms from known genes were also expressed in the other tissues, ranging from 116 (27%; liver) to 188 (43%; atrial appendage). In total, 384 (90%) of the new isoforms from known genes validated in at least one tissue. **(b)** Number of tissues in which isoforms were expressed. Of those isoforms that validated in GTEx tissues, 83 were expressed in all nine (largest bar), whereas the next highest group was for isoforms found in three tissues (76). **(c)** Tissues where isoforms expressed in only a single tissue were seen. As expected, most isoforms validated in only a single tissue were found in the prefrontal cortex (18), but several appeared to be “specific” to other, non-brain tissues. **(d-f)** deal with new isoforms from medically relevant genes (subset of new isoforms from known genes). **(d)** Proportion of new isoforms from medically relevant genes that validated in each tissue. **(e)** Same as (b) but for medically relevant genes. For **(f**,** i)**, colored dots indicate expression of isoforms that validated at the protein level in Aguzzoli-Heberle et al. [3] **(f)** log2(median CPM + 1) of each isoform expressed from the medically relevant genes. **(g-i)** deal with isoforms from new gene bodies. **(g)** Proportion of isoforms from new gene bodies that validated in each respective tissue. **(h)** Same as (b) and (e), but for new gene bodies. **(i)** Boxplot of the log2(median CPM + 1) of each isoform from new gene bodies.

For each tissue, we first quantified the proportion of the new isoforms that exceeded our threshold (median unique count ≥ 1 and median CPM > 1; Fig. 4a, d, g). For new isoforms from known gene bodies (nuclear and mitochondrial), we unsurprisingly found the greatest proportion of isoforms validated in the GTEx frontal cortex samples, where 336 (78%) of our recently discovered isoforms were expressed above our required threshold (Fig. 4a). Of the remaining 92 (22%) that did not meet our threshold, 52 (12%) were observed with 0 < median CPM ≤ 1, while still requiring the median unique counts ≥ 1. The large difference in sequencing depth between the two studies may explain why the remaining 10% did not validate in the GTEx frontal cortex data. Median sequencing depth per sample in Aguzzoli-Heberle et al. was 35.5 million aligned reads per sample [3] whereas median sequencing depth within the GTEx samples was 4.95 million aligned reads per sample [16]. We also found that 292 (67%) and 300 (69%) of our newly discovered RNA isoforms from known gene bodies were expressed in the cerebellar hemisphere (brain) and putamen (brain), respectively (Fig. 4a).

Many of the new RNA isoforms from known genes were expressed in the other tissues, ranging from 116 (27%; liver) to 188 (43% atrial appendage; Fig. 4a). In total, 447 and 384 isoforms validated in at least one of the nine tissues for all new isoforms and only isoforms from known genes, respectively.

Next, we quantified how many new isoforms from known gene bodies were expressed in multiple tissues (Fig. 4b). Surprisingly, of those isoforms that validated in the GTEx tissues, 83 were expressed in all nine (largest bar in Fig. 4b), whereas the next highest group was for isoforms found in three tissues with 76. Observing such a large proportion that validate in three tissues was unsurprising since three of the tissues are from brain—the organ where the isoforms were originally discovered—but observing so many expressed in all nine tissues was surprising. A potential explanation for why such a large proportion validated in all nine tissues could be that many of these RNA isoforms perform essential “housekeeping” functions across tissue types. Only deeper experimental work will be able to determine potential functionality with certitude.

For the 42 new RNA isoforms from known gene bodies expressed in a single tissue, we assessed which tissue they validated in (Fig. 4b, c) because isoforms being expressed in a single tissue could indicate tissue “specificity”. Even though these new isoforms were discovered in prefrontal cortex, having greater expression in another tissue may indicate the isoform is “more essential” in that tissue. Unsurprisingly, most of the new isoforms that validated in only a single tissue were found in the prefrontal cortex (18), but several appeared to be “specific” to other, non-brain tissues (Fig. 4c). In total, there were seven isoforms—one in cultured fibroblasts (*ATOX1*), one in each heart tissue (*MYADM* & *RORB-AS1*), two in skeletal muscle (*SPOUT1* & *ENSG00000288717*), and two in lung (*TMOD1* & *THEMIS2*; Fig. 4c; Supplemental Table S7).

We found similar validation patterns when limiting to new isoforms from known medically relevant genes (Fig. 4d, e), except only one validated in only a single tissue (*GAP43* in frontal cortex). Median expression for new medically relevant isoforms was comparable across all tissues (Fig. 4f). All eleven of the new isoforms from known genes that validated at the protein level in our previous work were also expressed in at least one GTEx tissue, two of which were from medically relevant genes (Fig. 4f).

### Many newly discovered gene bodies are expressed across the nine tissues

In addition to discovering new isoforms from known genes, we also previously discovered 267 isoforms from 245 new gene bodies [3]. Exactly 63 isoforms from the new genes validated in at least one tissue (Fig. 4g, h), where most that were expressed in only a single tissue were primarily seen in a brain tissue. Only five were expressed in all nine tissues (Fig. 4h).

Unsurprisingly, most isoforms from the new gene bodies were more highly expressed across the three brain tissues, though the median isoform expression across all tissues was below 3 CPM (Fig. 4i). Surprisingly, the highest median CPM values for isoforms from new gene bodies did not come from a brain region, however. Cultured fibroblasts and lung expressed BambuGene224803 at median CPM 48.26 and 19.61, respectively. BambuGene78526 has an isoform (BambuTx2076) expressed in heart left ventricle at 24.71 and heart atrial appendage at 18.04. The highest median CPM for an isoform from a new gene body in brain came from BambuGene121451, expressed in putamen at 16.78. Overall, 6.0% (16) of isoforms from new gene bodies were seen in at least one tissue above a CPM of 5. Of the three isoforms from new gene bodies that previously validated via mass spectrometry, only one was expressed in the GTEx samples, and it was only in two of the brain tissues (Fig. 4i).

### Newly discovered isoforms demonstrate potential tissue specificity or housekeeping roles

We were especially intrigued by the newly discovered RNA isoforms that fell into two specific categories: (1) those that only validated in a single tissue, suggesting potential tissue specificity; and (2) those that validated in all nine tissues, suggesting potential housekeeping roles. In both cases, these newly discovered RNA isoforms could have significant roles in human health and disease. To refine the list of isoforms showing potential tissue specificity or housekeeping roles, we performed differential expression using DESeq2, because simply separating isoforms by the number of tissues in which they are expressed is an overly simplistic approach.

#### Many isoforms exhibit “preferential” expression for specific tissues

Given we have data for two heart regions and three brain regions, we allowed isoforms to be “preferentially expressed” in up to three tissues for this analysis. For our purposes, we designated isoforms as preferentially expressed if they were positively and differentially expressed in up to three tissues compared to all other tissues (pairwise). Specifically, we required a log2-fold change ≥ 1, a false discovery rate (FDR) less than 0.1, and the isoform had to be upregulated relative to the other tissues. We were not interested in isoforms with lower expression relative to the other tissues because this would not suggest tissue specificity. We only included isoforms expressed with a median CPM > 1 in at least one tissue.

**Fig. 5:**
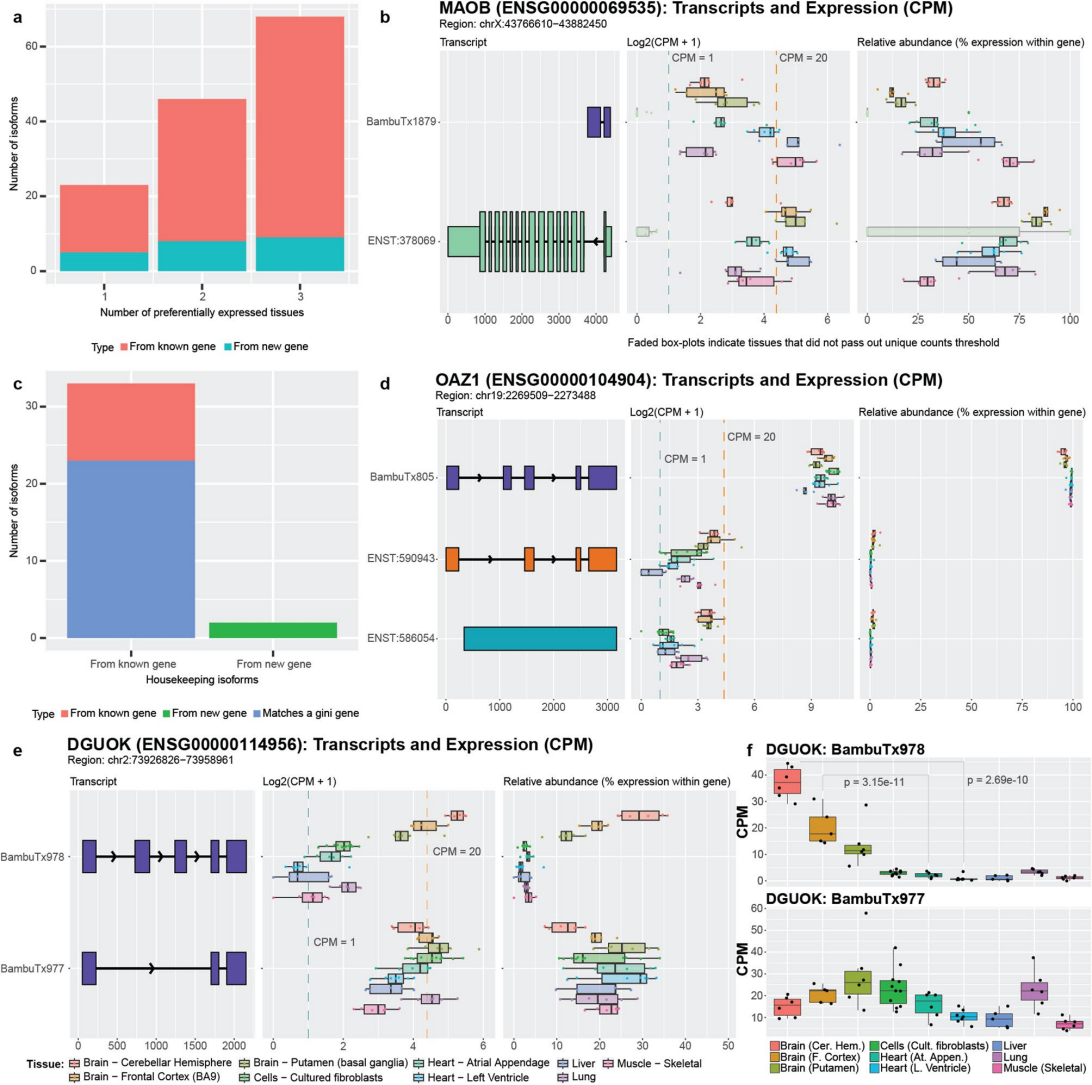
*Newly discovered isoforms may have preferential expression and/or housekeeping potential.* **(a)** Number of newly discovered isoforms that are preferentially expressed across one, two, and three tissues range from 18 to 59. For new gene bodies, they range from 5 to 9. **(b)** *MAOB* expression and relative abundance. The new isoform (BambuTx1879) is much shorter than the primary isoform but has a new exon. It is preferentially expressed across liver, muscle, and heart left ventricle (Supplemental Figure S39 has unedited plot); whether the new isoform is biologically functional is unknown. **(c)** Number of newly discovered isoforms that are expressed across all tissues within a log2-fold change of each other for known and new gene bodies, meeting our criteria for housekeeper expression patterns. 23 of the 35 (66%) isoforms that met our criteria for housekeeping expression patterns came from genes that were already identified as housekeeping genes by Joshi et al. [72], supporting our hypothesis that the other isoforms that met our criteria may also have housekeeping functions, including our newly discovered genes from Aguzzoli-Heberle et al. [3]. **(d)** *OAZ1* expression and relative abundance. *OAZ1* was previously identified as a housekeeping gene, though its housekeeping status has been debated. The new *OAZ1* isoform is consistent with our defined housekeeping expression pattern, being expressed across all nine tissues and are within log2-fold change of each other (Supplemental Figure S42 has unedited plot). Whether *OAZ1* fits criteria for a housekeeping gene may vary according to which isoform(s) are being measured in a given assay (e.g., different PCR primers). **(e)** The two newly discovered *DGUOK* isoforms suggest the gene may serve both preferential and housekeeping biological needs. BambuTx978 appears to be preferentially expressed, while BambuTx977 exhibits housekeeping-like expression behavior (Supplemental Figure S43 has unedited plot) **(f)** CPM values of the two *DGUOK* isoforms, showing clearly unique expression profiles across the nine tissues included in this study

We identified 23 isoforms that were preferentially expressed in a single tissue, 46 preferentially expressed in two tissues, and 68 in three tissues (Fig. 5a). Exactly 22 were from new genes (5 in single tissue, 8 in two tissues, and 9 in three tissues). Unsurprisingly, most of these isoforms were preferentially expressed in at least one brain tissue (12, 39, and 62 from single tissue, two tissues, and three tissues, respectively).

Isoforms preferentially expressed in tissues other than brain were especially intriguing, given they were discovered in brain tissue. *MAOB*, for example, encodes an enzyme that helps break down neurotransmitters (often in the brain) [70] and is a target for treating Parkinson’s disease symptoms. Parkinson’s patients suffer from tremors, rigidity, bradykinesia (slow movement), and resting tremors, in part because of insufficient dopamine levels. MAO-B inhibitors are used to decrease *MAOB* activity and increase dopamine levels, therefore mitigating symptoms in Parkinson’s disease patients [71]. According to our analyses, the newly discovered isoform for *MAOB* is preferentially expressed in muscle, liver, and the left ventricle of the heart, which may imply a distinct function in these tissues (median CPM from 17.37 to 32.48, next highest is 5.87 in brain [putamen]; Fig. 5b; Supplemental Figure S39). Output from IsoformSwitchAnalyzeR predicts that the newly discovered isoform is non-coding (Supplemental Figure S40). Whether this isoform has functional consequences remains unknown. This preferential expression in other tissues highlights the need for a more in-depth interrogation of *MAOB* isoforms.

#### Newly discovered isoforms exhibit potential housekeeping expression patterns

Opposite of genes and RNA isoforms that demonstrate tissue specificity or preference are genes and RNA isoforms that perform housekeeping functions—genes and isoforms that are broadly required across cells and tissues for proper function. Here, we consider isoforms as demonstrating “housekeeping” expression patterns if they were expressed in all nine tissues above our threshold and demonstrated relatively similar expression across all nine tissues (i.e., were within a log2-fold change of two for all tissues). Using these thresholds, we identified 35 of the newly discovered isoforms that met our criteria for housekeeping expression patterns (Fig. 5c). As potential validation, 23 of these isoforms matched genes listed in Joshi et al. [72] as housekeeping genes (or Gini genes) using GTEx data, based on the Gini coefficient—a statistical measure of the inequality among groups, often used in economics where a lower Gini coefficient indicates lower income inequality. We further calculated the Gini coefficient for each of the 35 isoforms and found that 19 isoforms had a Gini coefficient < 0.3; 13 of the isoforms were from Gini genes listed in the above paper (Supplemental Figure S41; Supplemental Table S8). Two of the housekeeping isoforms we found come from newly discovered genes.

One of the Gini genes is *OAZ1*, which has been listed as a housekeeping gene [72, 73], though some publications disagree with this assignment [74, 75]. These discrepancies could be due to the lack of annotation for the newly discovered isoform which we classify as housekeeping and is highly expressed (median CPM from 413.71 to 1,341.99; Fig. 5d; Supplemental Figure S42).

There were two genes, *DGUOK* and *EEF1AKMT1*, that were especially interesting because they both had an isoform that met our criteria for preferential expression, while the other isoform met our criteria for a potential housekeeping isoform. Both genes were also identified as housekeeping genes by Joshi et al. [72]. One of these genes, *DGUOK*, encodes an enzyme that is critical in mitochondria. As most cells contain mitochondria, it is unsurprising that *DGUOK* would be classified as a housekeeping gene based on the Gini index and that a specific isoform could be the primary contributor for its housekeeping status, as is the case with one of the new isoforms, BambuTx977. The other new isoform (BambuTx978), however, is preferentially expressed in brain (Fig. 5e, f; Supplemental Figure S43). This is supported by output from IsoformSwitchAnalyzeR which indicates that this isoform has a different usage ratio for the brain regions compared to all other tissues (Supplemental Table S6). This preference in brain may be because the brain has high energy demands and may require tissue-specific functions from *DGUOK.* Based on this hypothesis, however, it is unclear why an additional isoform would be required instead of simply increasing expression for another isoform in brain regions. Additionally curious is that IsoformSwitchAnalyzeR output predicts both isoforms as non-coding (Supplemental Figure S44). Only deeper investigation will be able to assess whether these new *DGUOK* isoforms have essential housekeeping and brain-specific functions, respectively, and what those functions might be.

### Web application for GTEx RNA isoform expression

Using the GTEx data from this study, we created a resource for fellow researchers to explore and query this RNA isoform expression data across nine human tissues. The web app can be found at https://ebbertlab.com/gtex_rna_isoform_seq.html.

## Discussion

Alternative splicing has long been known to play an important role in the biology of complex organisms, like humans, but our understanding of the role various isoforms play is limited. In our opinion, it seems unlikely that 20,000+ protein-coding genes performing a single function can enable such complex organisms [3]. Thus, here, we have provided a broad survey of the RNA isoform landscape to demonstrate both the complexity and diversity of RNA isoforms across nine human tissues using data generated by Glinos et al. [16] and Aguzzoli-Heberle et al. [3]. We have also tried to demonstrate how little is known about the various isoforms and why it is essential to fully characterize and quantify individual RNA isoform expression across the human cell types, tissues, and lifespan. One of the most important next steps in biology will be to determine the function of individual RNA isoforms for every gene.

Short-read sequencing technologies have been a major boon for assessing total gene expression across a range of tissue and cell types, and across various diseases, but short-read sequencing struggles to accurately quantify expression for individual isoforms. Essentially, the expression of every isoform within a gene is collapsed into a single expression measurement because of the technical limitations of short-read sequencing data.

Long-read sequencing, however, provides a major improvement in our ability to accurately characterize and quantify individual RNA isoforms. Here, we also demonstrate that accurately quantifying expression for individual RNA isoforms is not a solved problem, as have others [3, 14, 21]. For example, these studies highlight the challenges associated with quantifying RNA isoforms given that a large percentage of reads cannot be uniquely assigned to a single isoform, though it is still a major improvement over short-read sequencing data.

Perhaps the most important discussion item arising from this work, however, is whether the many isoforms expressed for certain genes have distinctly different, biologically meaningful functions, or are simply biological redundancy or spurious splicing. We already know of various examples where the distinct isoforms for a given gene are not only biologically meaningful, but essential. *BCL-X* (*BCL2L1*) [8] is a classic example, where one isoform is pro-apoptotic (BCL-Xs) while the other is anti-apoptotic (BCL-XL). Other examples (e.g., *RAP1GDS1* and *TRPM3*) are known to have more subtle functional differences. Subtle differences do not necessarily imply they are insignificant, however. We anticipate that further study of isoforms will prove fruitful for many diseases, such as Alzheimer’s disease. As we continue to learn more about individual isoforms, we expect to find some proportion that result from biological redundancy or spurious alternative splicing, but we also expect that many will have biological significance. Ultimately, only deeper studies—including experimental work—will be able to fully address these questions.

## Conclusion

As sequencing technology (including preserving natural RNA) and associated algorithms advance, our ability to study individual RNA isoforms will improve dramatically. Here, we provided a broad, bioinformatic survey of the RNA isoform landscape, demonstrating the isoform diversity across nine tissues and emphasizing the need to better understand how individual isoforms from a single gene body contribute to human health and disease. We found genes whose isoform expression patterns differed in interesting and potentially significant ways and we validated isoforms recently discovered in Aguzzoli-Heberle et al. [3]. We also identified isoforms that exhibit patterns consistent with preferential expression for a given set of tissues and others that demonstrated potential housekeeping expression patterns. The breadth and depth of what can and should be studied is vast and warrants significant additional efforts to understand the complexities and subtleties of human health and disease.

## Methods

### Downloading GTEx data, read pre-processing, genomic alignment, and quality control

We obtained the publicly available GTEx nanopore long-read cDNA RNAseq data from Glinos et al. [16] for this study through the AnVIL portal [76]. The data consists of 88 GTEx samples from 15 different human tissues and cell-lines. The data were re-processed using the same methods used in our recent paper by Aguzzoli-Heberle et al. [3].

Briefly, we pre-processed the cDNA data with Pychopper (version 2.7.6), applying settings compatible with the PCS109 sequencing kit, since the GTEx data were sequenced using this chemistry. Pychopper discards any reads missing primer sequences on either end and recovers reads that contain primer sequence in the middle, which result from fused molecules. Pychopper also orients the reads according to their genomic strand and removes any adapter or primer sequences. We then aligned the pre-processed reads to GRCh38 with minimap2 version 2.26-r1175 [77], including the “-x splice” (preset option to allow spliced alignments; parameters settings: -k15 -w5 --splice -g2k -G200k -A1 -B2 -O2,32 -E1,0 -b0 -C9 -z200 -ub --junc-bonus=9 --cap-sw-mem=0 --splice-flank=yes) and “-uf” (to identify splicing sites using the transcript strand) alignment parameters. We used the GRCh38 reference genome without alternate contigs for alignment. We removed reads that mapped with a Mapping Quality (MAPQ) score below 10 using samtools (version 1.17). The BAM files were then sorted by genomic coordinates and indexed using samtools. See **Code availability** for all scripts. Due to minor differences between our pipelines (i.e., we employed Pychopper), the number of reads we included for isoform quantification in Bambu is lower than what Glinos et al. used. See Supplemental Table S1 for number of reads analyzed by Bambu. See Glinos et al. [16] for quality control data for these samples.

### Sample inclusion criteria

Glinos et al. [16] originally sequenced 88 cDNA GTEx samples across 15 tissues and cell lines using the Oxford Nanopore Technologies MinION. For our analyses, we ultimately only included nine of the 15 tissues after applying the following inclusion criteria: (1) we only included tissues with samples from at least five unique subjects; (2) excluded samples with experimental conditions (i.e., *PTBP1* knockdown); (3) excluded technical replicates; (4) excluded samples with < 1,000,000 reads; and (5) excluded any samples that did not cluster with their respective tissue group based on a principal component analysis (PCA). In the end, we retained 58 samples across nine tissues, including cerebellar hemisphere (brain), frontal cortex (brain), putamen (brain), cultured fibroblasts, atrial appendage (heart), left ventricle (heart), liver, lung, and skeletal muscle. When selecting which sample to retain among technical replicates, we chose the sample with the highest number of total reads that was below the maximum total reads for that tissue (to avoid including a sample that would be an outlier). We then performed a PCA analysis, using the DESeq2 [78] (version 1.42.0) plotPCA function after DESeq2 normalization on the total counts matrix from Bambu (excluding the filtered samples) and excluded one liver sample due to poor clustering (Supplemental Figures S1,S2). The full list of included samples can be found in Supplemental Table S1.

### GTEx analyses

To limit false positives and to be consistent with our recent work in Aguzzoli-Heberle et al. [3] we selected CPM = 1 as the noise threshold and further required a median unique counts ≥ 1, to exclude any isoforms that have not been consistently observed uniquely across samples. Unless otherwise specified, we only included isoforms with a median CPM > 1 (and median unique counts ≥ 1) in our analyses. We calculated counts-per-million (CPM), gene CPM, relative abundance, and the median relative abundance for each isoform using Bambu’s “total counts” metric. For reference, relative abundance is the percent expression for a given isoform within a gene. We used Ensembl v109 annotations [1] (2023) combined with the 700 new isoforms discovered in Aguzzoli-Heberle et al. [3] to quantify isoform expression with Bambu. We quantified the number of isoforms expressed within each tissue across a range of CPM thresholds from 0 to 10.01 in 0.01 increments, as shown in Fig. 2a and further stratified these analyses across a range of gene and isoform types, including protein-coding isoforms, medically relevant isoforms, protein-coding isoforms from medically relevant genes, and brain-disease-relevant isoforms. Medically relevant genes were as defined by Wagner et al. [22] with additions from Aguzzoli-Heberle et al. [3]. We generated an upset plot evaluating the overlap of isoform expression across several combinations of different tissues in rank order using R package ggupset version 0.3.0 [79]. We also assessed the significance of protein-coding to non-protein-coding isoforms between cerebellar hemisphere and heart’s left ventricle by running a Chi-square test.

#### Isoform heatmap across nine GTEx tissues and pathway analysis

We wanted to identify genes that have a high number of isoforms expressed, so we created a list of genes that express > 5 isoforms in at least one tissue (above our ‘noise’ thresholds described above). Using those genes and the number of isoforms expressed by tissue, we created a clustered heatmap with the raw isoform numbers (not expression values). We performed pathway analyses using Metascape [60] (accessed January 2024). We also plotted the relationship between total gene expression and number of isoforms expressed. We (1) ranked expression, isoform counts, and gene length, (2) calculated the expression and isoform count residuals, controlling for gene length, (3) ran a Spearman correlation test for the significance of the correlation, and (4) plotted the residuals.

#### IsoformSwitchAnalyzeR

 To assess protein domains and differential isoform usage, we selected a list of 14 genes to run through the following tools: IsoformSwitchAnalyseR [24], CPAT3 [25], Pfam [26], IUPred2A [27], SignalP6 [28], DeepLoc2 [29], and DeepTMHMM [30]. These genes were chosen based on the other analyses we ran. We passed the raw counts and CPM matrices (filtered to isoforms from the 14 genes) to IsoformSwitchAnalyzeR. We used the preFilter function to remove isoforms that were not expressed CPM > 1 in at least one tissue. We ran isoformSwitchTestSatuRn (due to the large number of pairwise comparisons (36) per isoform) with reduceToSwitchingGenes = FALSE and reduceFurtherToGenesWithConsequencePotential = FALSE. We also added open reading frames (ORFs) for those that did not have any, selecting the longest ORF where it started on a canonical ORF when possible. Then the nucleotide and amino acid sequences for each isoform were outputted and used in the additional external tools. The external tools and their output are described below. After gathering the outputs and adding them to the IsoformSwitchAnalyzeR object, we plotted the isoforms for each gene, visually depicting the outputs from the various tools. P-values for isoform switch analysis are FDR corrected (by IsoformSwitchAnalyzeR) and the full table of these values are available in Supplemental Table S6.

We have multiple singularity containers for the above-mentioned tools, but due to restrictions, we are not allowed to release our containers containing IUPred2A, SignalP6, and DeepLoc2, though the definition files are available. The other container is available for download (see **Code Availability**).

##### CPAT3

We passed the nucleotide sequences to CPAT3 to predict coding potential. In order to pass the results back to IsoformSwitchAnalyzeR, we had to reformat the output file to match the CPAT2 web results output, as IsoformSwitchAnalyzeR currently is not updated for CPAT3 output file format. We used 0.364 as the coding cutoff as this is suggested by CPAT for humans and they state that the sensitivity and specificity for this cutoff is 0.966 [25].

##### Pfam

We passed the amino acid fasta file to Pfam to predict protein families present in the isoform sequences.

##### IUPred2A

We passed the amino acid fasta file to IUPred2A to predict instrindically disordered proteins/protein regions.

##### SignalP6

We passed the amino acid fasta file to SignalP6 to predict signal peptides present in the isoforms.

##### DeepLoc2

We passed the amino acid fasta file to DeepLoc2 to predict the location of isoforms in the cell. We had to reformat the output of this to pass back to IsoformSwitchAnalyzeR.

##### DeepTMHMM

We passed the amino acid fasta file to DeepTMHMM to predict the topology of the protein.

### New isoforms from Aguzzoli-Heberle et al. [3]

To assess expression patterns for the 700 new isoforms reported in Aguzzoli-Heberle et al. [3], we separated the isoforms into new isoforms from known genes (433 isoforms) and new isoforms from new gene bodies (267 isoforms) and created an additional subset of new isoforms from medically relevant genes (54 isoforms). Note that in Aguzzoli-Heberle et al., they reported 53 isoforms in this category—the discrepancy between these numbers is due to filtering out the spliced mitochondrial isoform, which we do not exclude here. We then calculated the proportion of new isoforms present in each tissue at a median unique counts ≥ 1 and median CPM > 1 (as well as median CPM > 0) by taking the number of isoforms expressed in the group and dividing it by the total number of isoforms possible in the group. We also calculated the number of tissues each isoform was expressed in at a median CPM > 1. Taking the isoforms that were only expressed in a single tissue, we looked at which tissue was expressing the isoform. We plotted the log2(median CPM + 1) of the isoforms in the new from medically relevant genes and new genes categories at a CPM > 0.

### Potential preferential and housekeeping isoforms from new isoforms

To assess whether any of the new isoforms were potentially preferentially expressed in a given set of tissues, or exhibit housekeeping-like behavior (i.e. expressed across all tissues), we performed pairwise differential expression analyses between each tissue pair for each isoform using DESeq2 [78] (normalizations using the total counts matrix with all isoforms, then filtering to only newly discovered isoforms for comparisons). To meet our criteria for preferential tissue expression, we required a log2-fold change > 1, a false discovery rate (FDR) less than 0.1, and the isoform had to be upregulated relative to the other tissues. To meet potential housekeeper criteria, we required isoforms to be expressed in all nine tissues above our noise threshold and demonstrate similar expression across all nine tissues (i.e., within a log2-fold change of two for all pairwise tissue comparisons).

Further, we calculated the Gini coefficient for those isoforms that were discovered in Aguzzoli-Heberle et al. [3]. We did this using the R package DescTools. We chose to use the cutoff of 0.3 to determine the Gini isoforms and then compared them to the isoforms we identified using our pairwise differential expression method and the Gini genes identified in Joshi et al. [72].

### Figures and tables

We created figures and tables using a variety of python (version 3.11.3) and R (version 4.3.1) scripts. Some figures were downloaded from our Rshiny app (R version 4.3.0). The IsoformSwitchAnalyzeR analyses were done using R version 4.4.3. All scripts are available on GitHub (see **Code availability**). Isoform structures were visualized using R package ggtranscript [80] (version 0.99.9). Final figures were assembled using Adobe Illustrator.

### Singularity containers

The singularity containers used for these analyses are available for download from sylabs, and our github repository has the definition files we used for building them. There are two containers we were not able to publish, as the tools in them are proprietary, but the definition files for how we built the containers are available. We would encourage pulling the pre-built containers when possible as we cannot guarantee the same software versions if the container is rebuilt. See our github singularity directory readme for how to download the containers.

## Supplementary Information

Below is the link to the electronic supplementary material.

Supplementary Material 1

Supplementary Material 2

## Data Availability

Ensembl v109 annotation was downloaded from the following link: https://ftp.ensembl.org/pub/release-109/gtf/homo_sapiens/Homo_sapiens.GRCh38.109.gtf.gz. GTEx long-read RNAseq data used is available through the AnVIL project at the following link: https://anvil.terra.bio/#workspaces/anvil-datastorage/AnVIL_GTEx_V9_hg38. GTEx counts matrices from Bambu can be downloaded here: 10.5281/zenodo.17092739. Data can be visualized on the Ebbert Lab website at https://ebbertlab.com/gtex_rna_isoform_seq.html and the deep long-read frontal cortex data from Aguzzoli-Heberle et al. can be viewed at https://ebbertlab.com/brain_rna_isoform_seq.html.

## Code availability

All code used in the manuscript is available at: https://github.com/UK-SBCoA-EbbertLab/landscape_of_RNA_isoform_expression.
