# Supplementary material for: A bioinformatic survey of RNA isoform diversity and expression across 9 GTEx tissues using long-read sequencing data
Source: BMC Genomics. 2025 Dec 5;26:1078. doi: 10.1186/s12864-025-11919-w (PMC12679790; doi:10.1186/s12864-025-11919-w)
Supplement: Supplementary file 1 — Supplementary Material 1 [file 12864_2025_11919_MOESM1_ESM.pdf]

Supplemental Tables:

Supplemental Table 1: Samples used by tissue with the number of primary aligned reads with MAPQ >= 10 from our analysis

Supplemental Table 2: Number of isoforms expressed per gene, based on a unique counts threshold (across all samples combined; unique counts thresholds—1, 5, 10, and 20)

Supplemental Table 3: Number of genes expressing at least N isoforms (based on unique counts, all samples combined) from all genes, protein-coding genes, and medically relevant genes.

Supplemental Table 4: Number of genes expressing at least N isoforms (by tissue) from all genes or protein-coding genes.

Supplemental Table 5: Genes expressing more than five isoforms in at least one tissue and the number of isoforms expressed per tissue for those genes.

Supplemental Table 6: Pairwise comparison output from IsoformSwitchAnalyzeR and associated tools for differential isoform usage (t-test).

Supplemental Table 7: List of new isoforms (from Aguzzoli-Heberle et al.) expressed in a single tissue above our noise thresholds.

Supplemental Table 8: Gini coefficients for newly discovered isoforms that passed our criteria for being considered housekeeping.

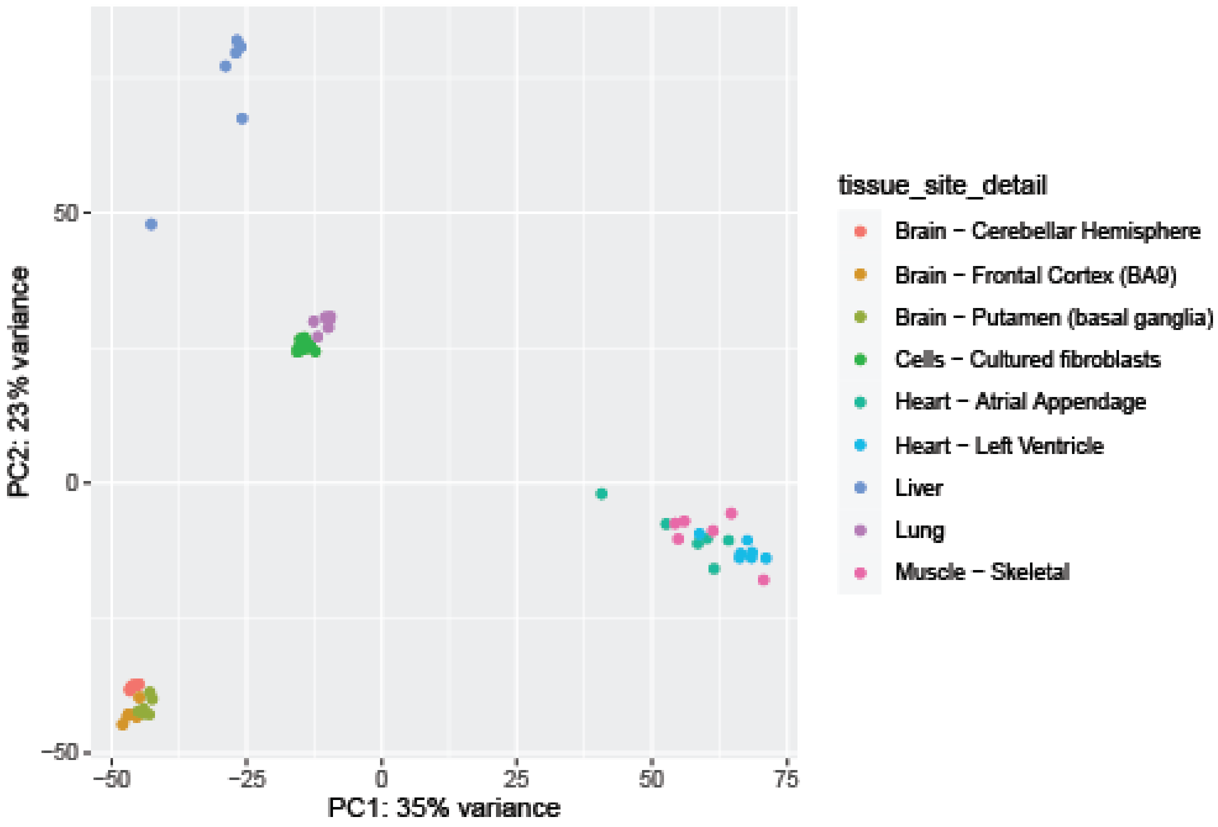

Supplemental Figure 1: DESeq2 PCA after filtering out technical replicates/experimental samples and those with low read counts.

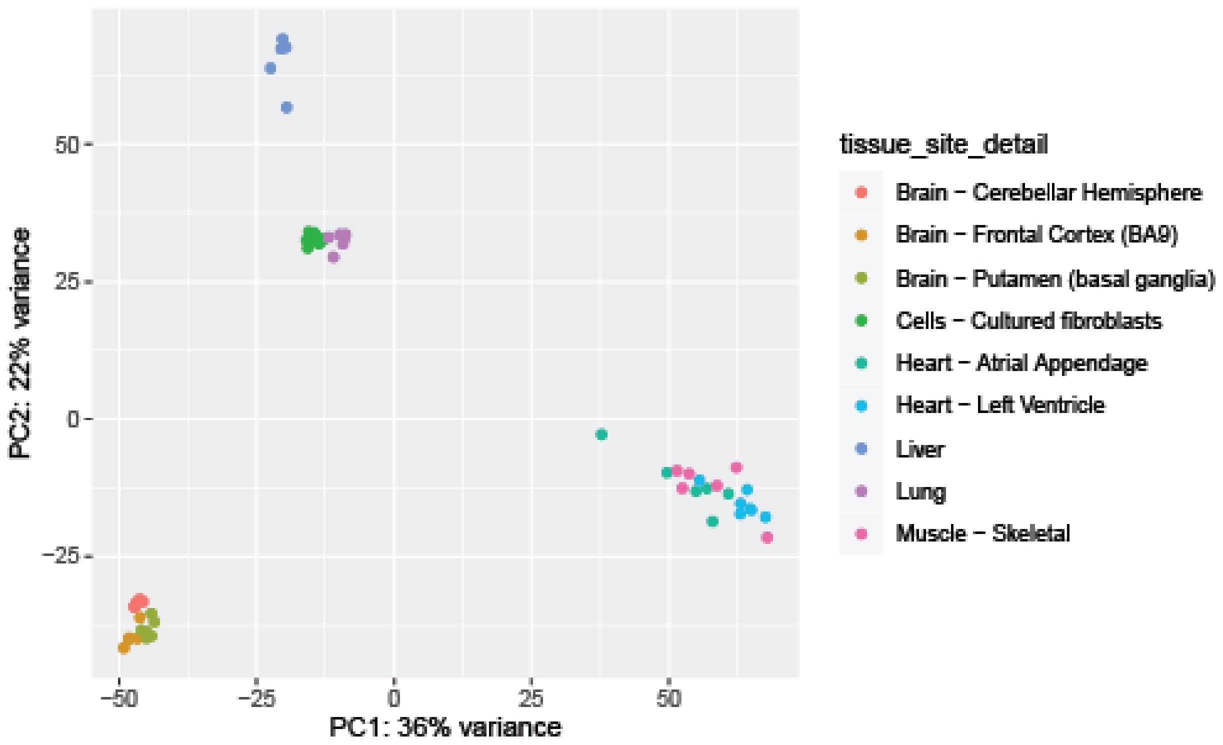

Supplemental Figure 2: Final DESeq2 PCA after sample with low clustering is removed.

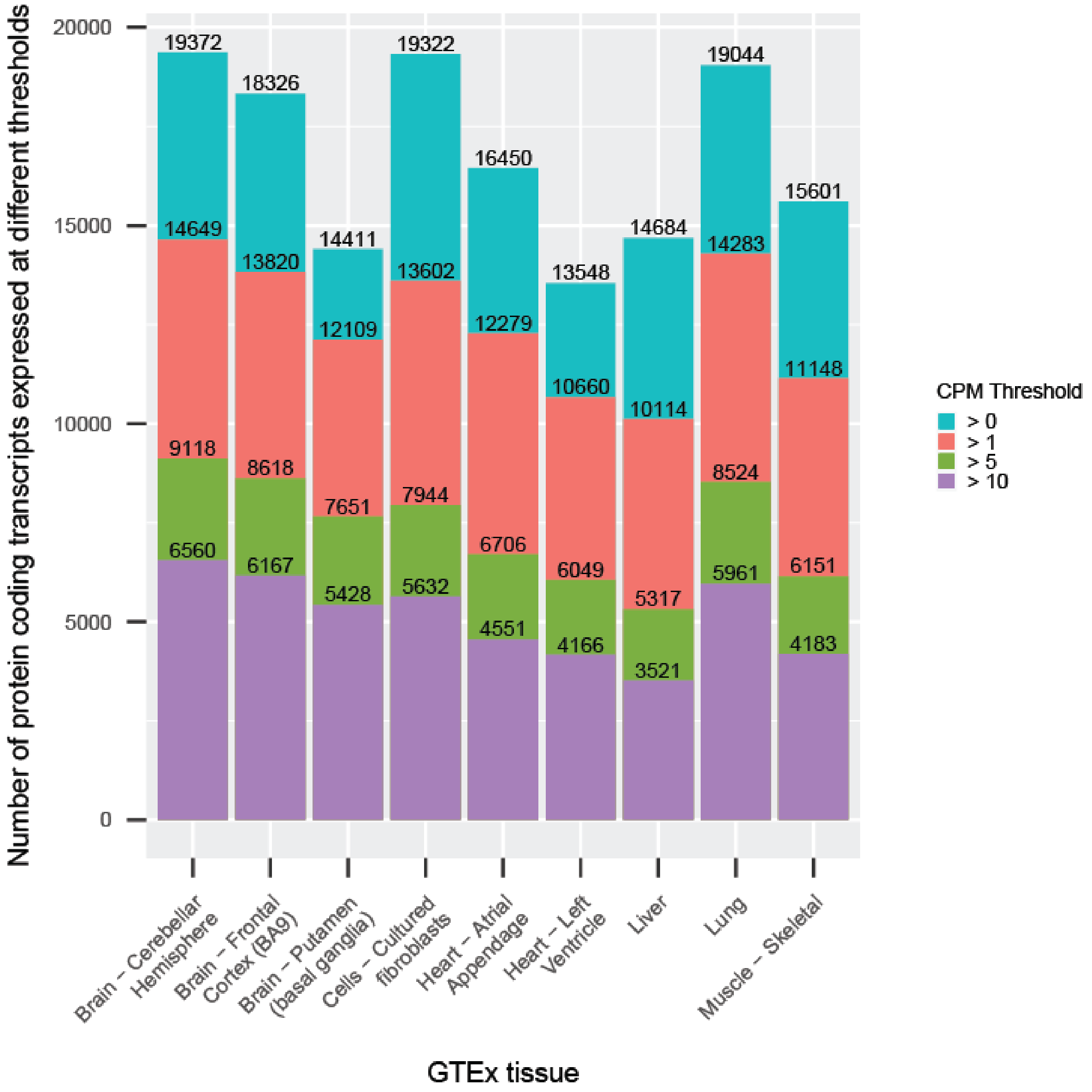

Supplemental Figure 3: Number of protein-coding isoforms expressed in each tissue across four thresholds. Number of protein-coding isoforms that passed four different thresholds in each tissue. The pink is the normal threshold used throughout this paper. Others are reported for interest.

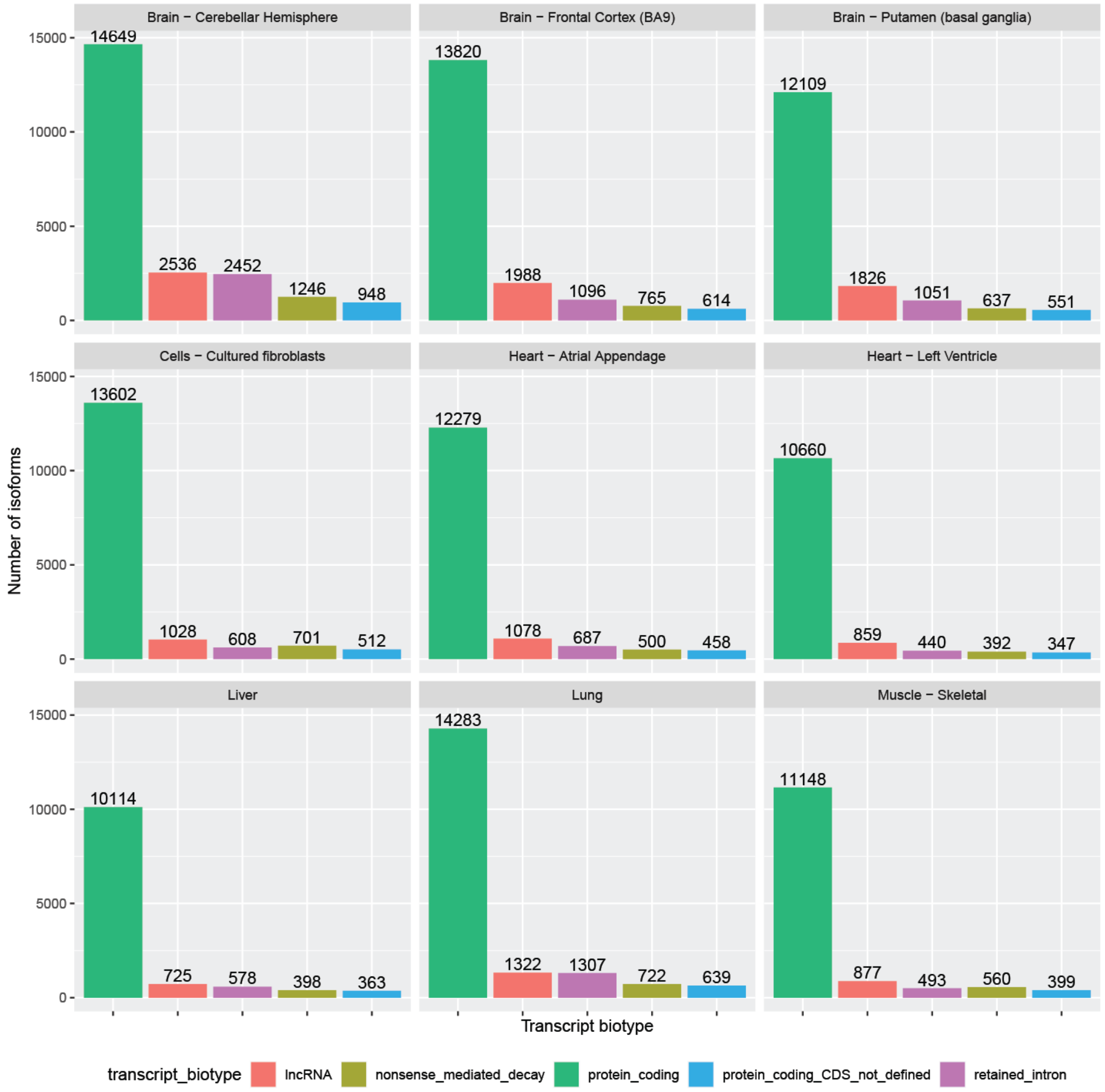

Supplemental Figure 4: Top five transcript biotypes expressed for each tissue. When compared with Figure1e in Page et al. the order of the most expressed biotypes does not match the order of the most annotated transcript biotypes. Note, skeletal muscle and cultured fibroblasts also differ from the other tissues in that nonsense mediated decay is a more prevalent biotype than retained intron.

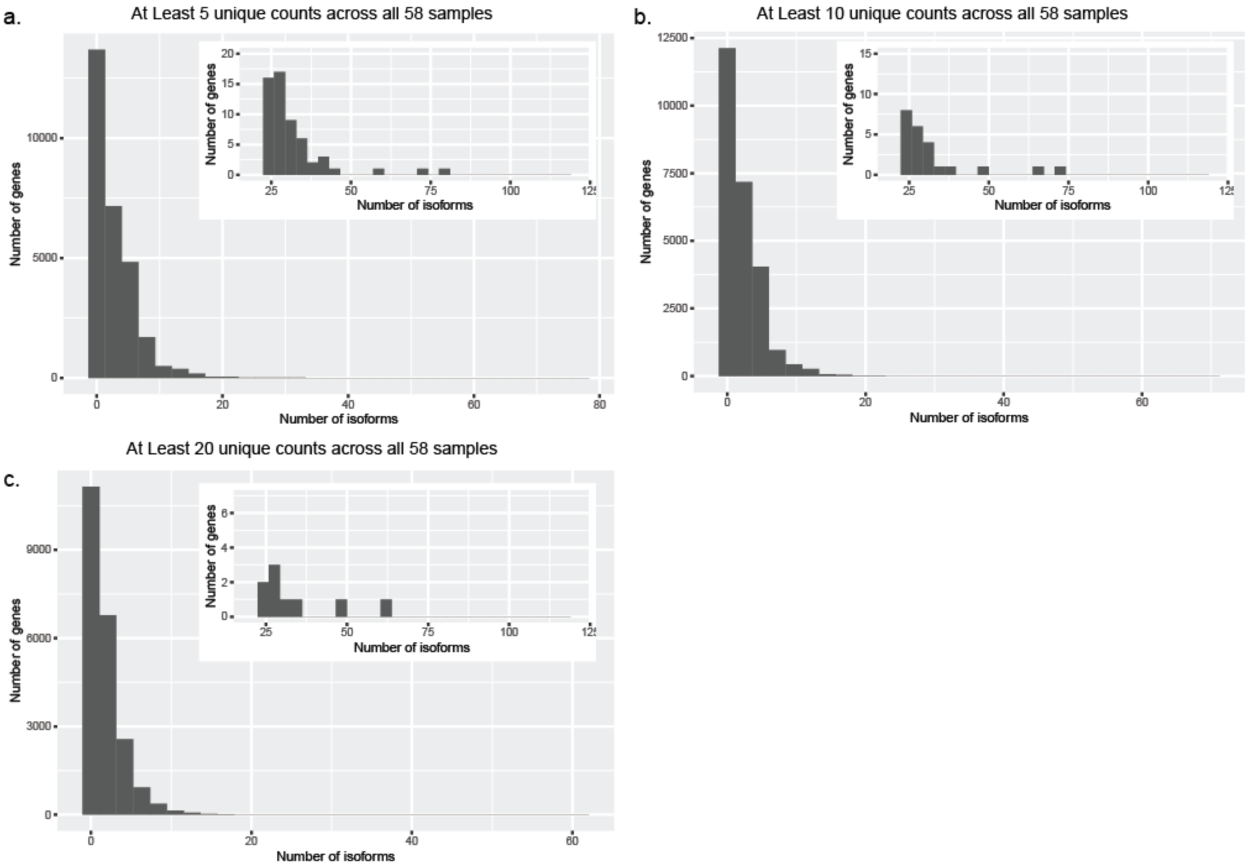

Supplemental Figure 5: Isoforms expressed per gene based on unique counts. Histograms of number of isoforms expressed per gene with at least **a.** 5, **b.** 10, or **c.** 20 unique counts across all 58 samples. Nested plots show a zoomed in portion of the histogram.

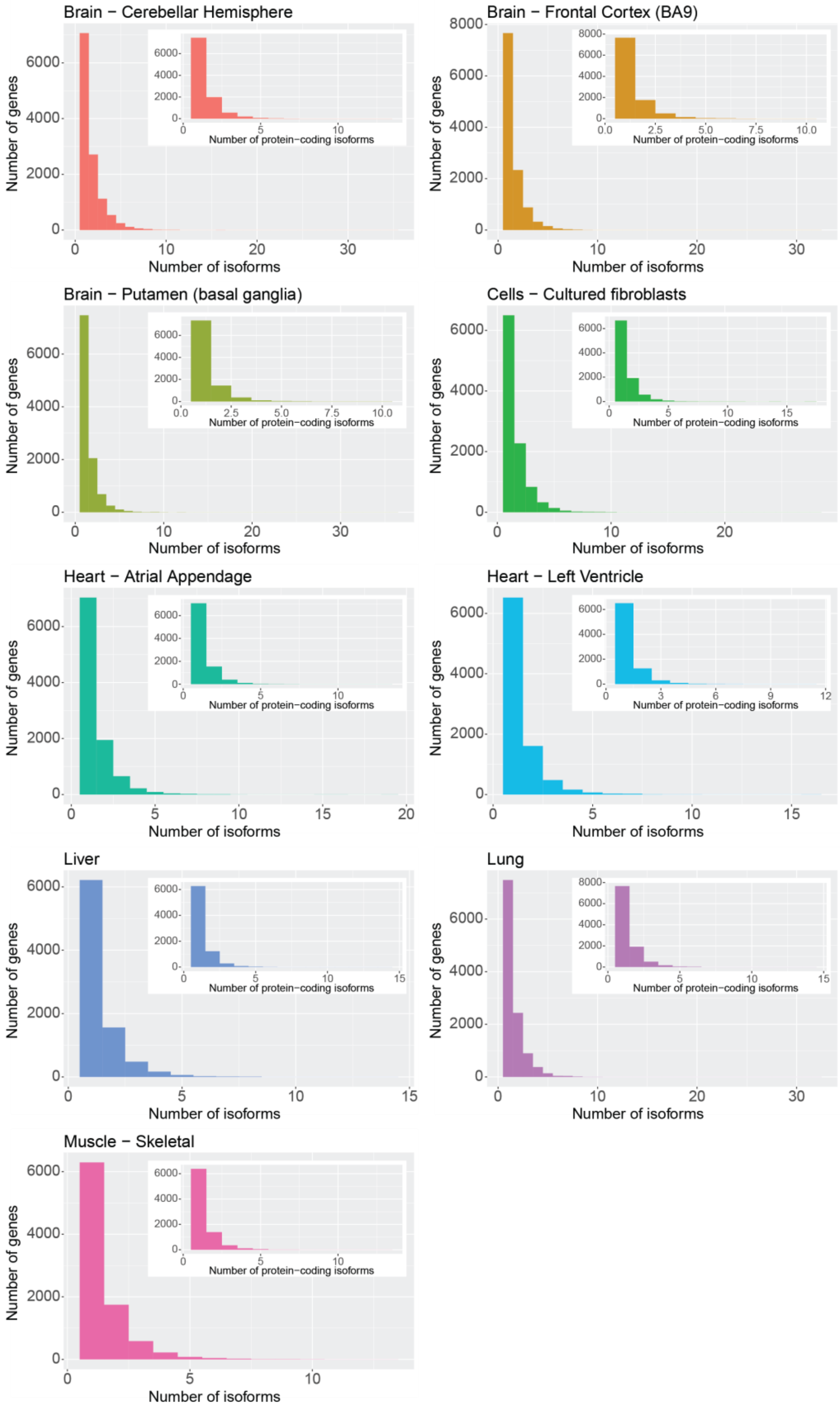

Supplemental Figure 6: Isoforms expressed per gene by tissue. Histograms of number of isoforms expressed per gene. Nested histograms show the number of protein-coding isoforms expressed per gene.

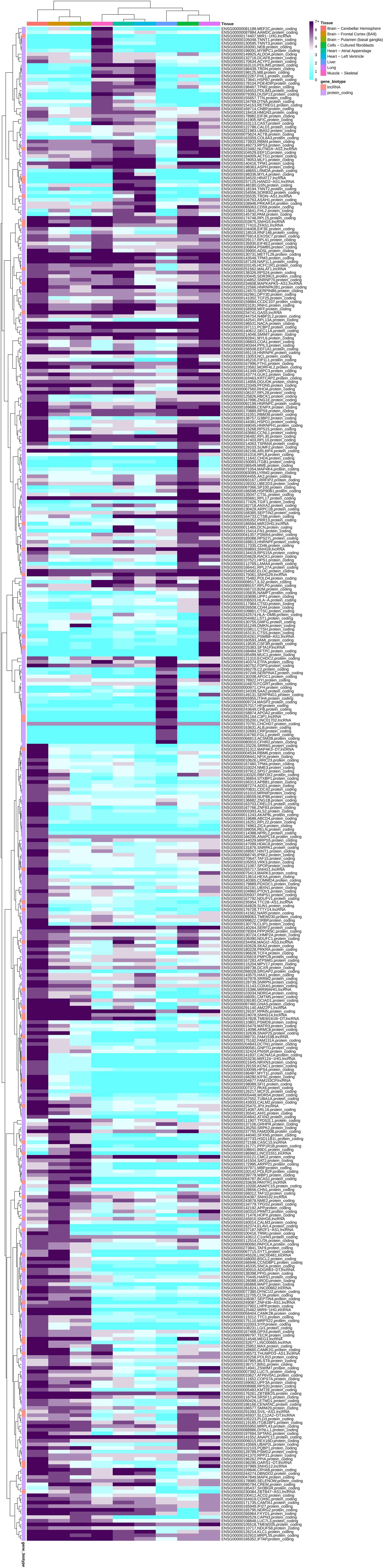
Tissue
Brain – Cerebellar Hemisphere
Brain – Frontal Cortex (BA9)
Brain – Putamen (basal ganglia)
Cells – Cultured fibroblasts
Heart – Atrial Appendage
Heart – Left Ventricle
Liver
Lung
Muscle – Skeletal
gene_biotype
lncRNA
protein_coding
7+
6
5
4
3
2
1
0
gene_biotype

Supplemental Figure 7: Clustered heatmap of number of isoforms expressed per gene. Clustered heatmap of 416 genes that expressed more than five isoforms in at least one tissue, including the GeneID's, Gene symbol (if available), and gene biotype (if available).

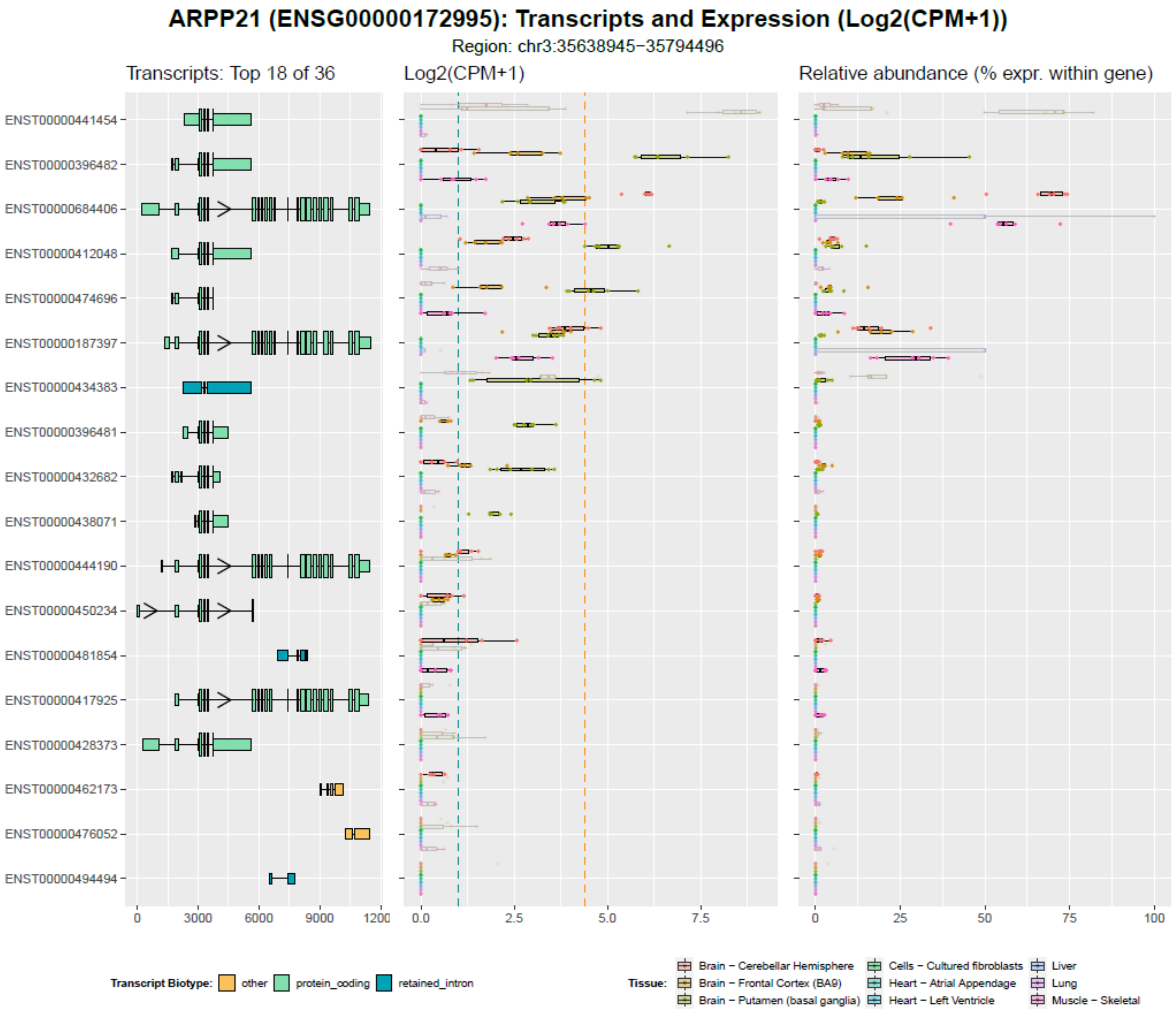

Supplemental Figure 8: ARPP21 isoforms, expression, and relative abundance. Isoforms expressed above a median CPM > 1 are considered in our analyses. Faded box-plots represent tissues where the isoform did not pass our unique counts threshold (median unique counts ≥ 1), and therefore are not included in our analyses. Blue dashed line is CPM = 1 and orange dashed line is CPM = 20. Putamen expressing the greatest number of isoforms.

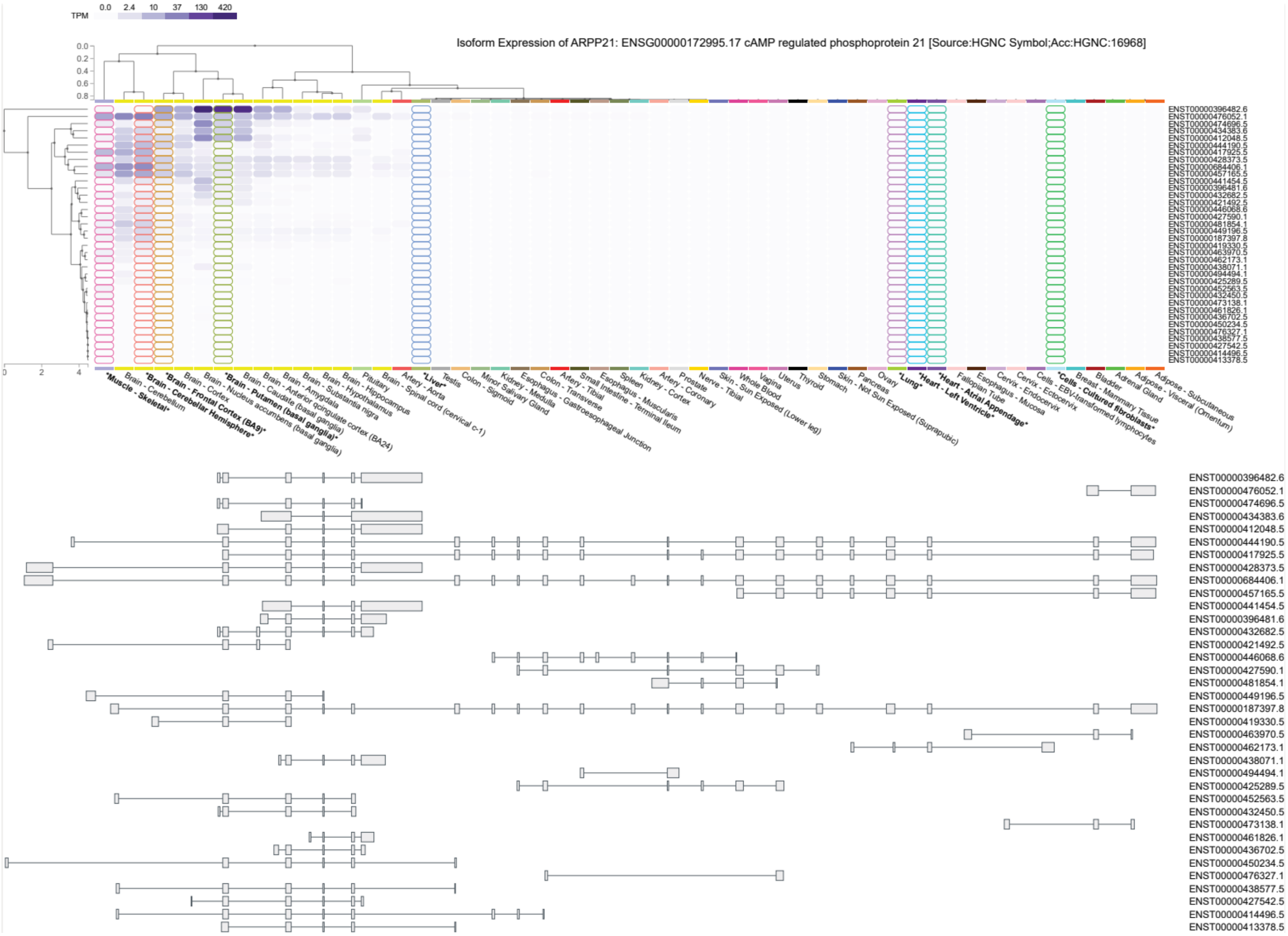

Supplemental Figure 9: ARPP21 GTEx short read isoform expression Screenshot of short read GTEx isoform expression for ARPP21. Tissues used in our long read data are highlighted.

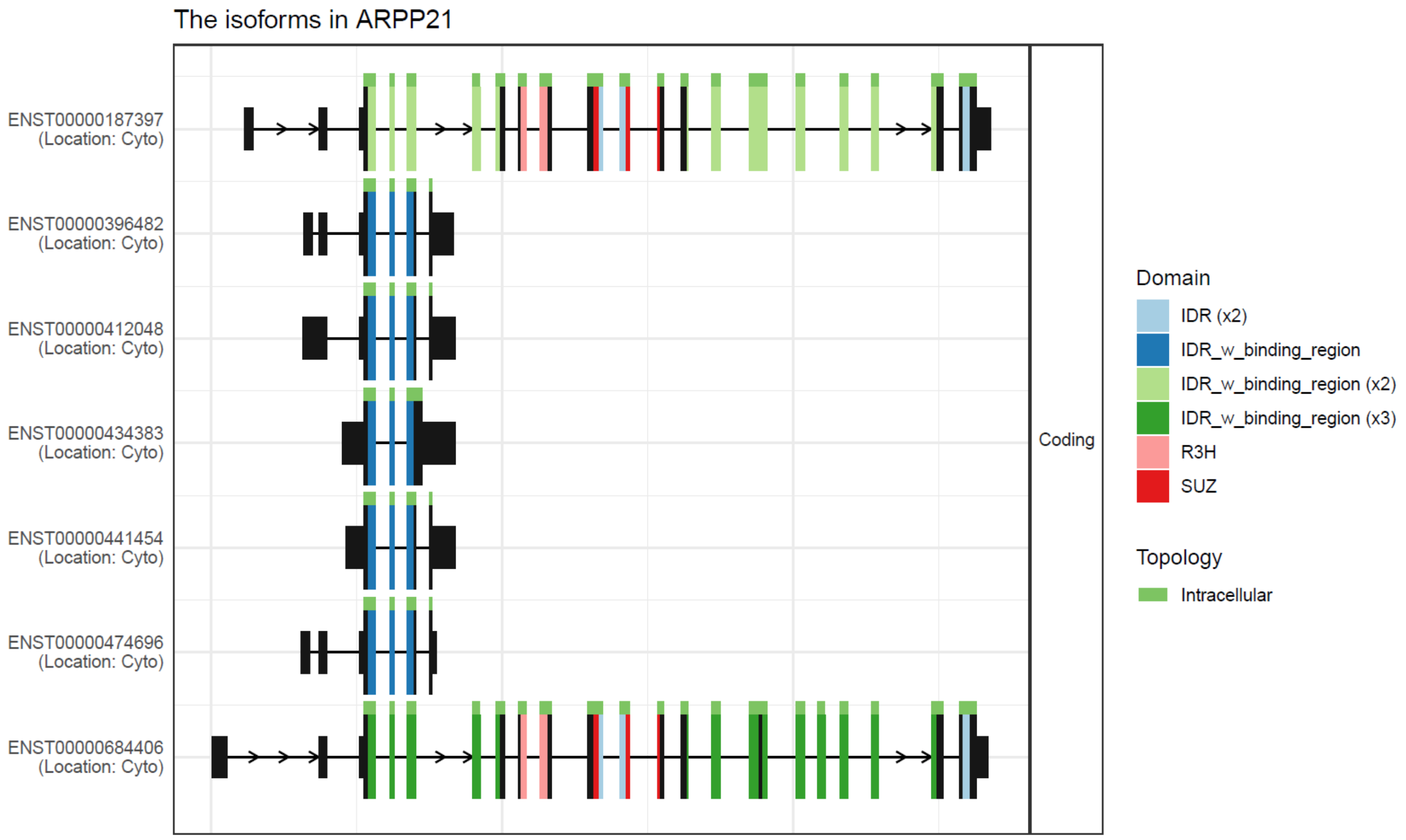

Supplemental Figure 10: ARPP21 IsoformSwitchAnalyzeR isoform plot output. The five shorter isoforms all seem to have the same domains. The longer isoforms are more complex.

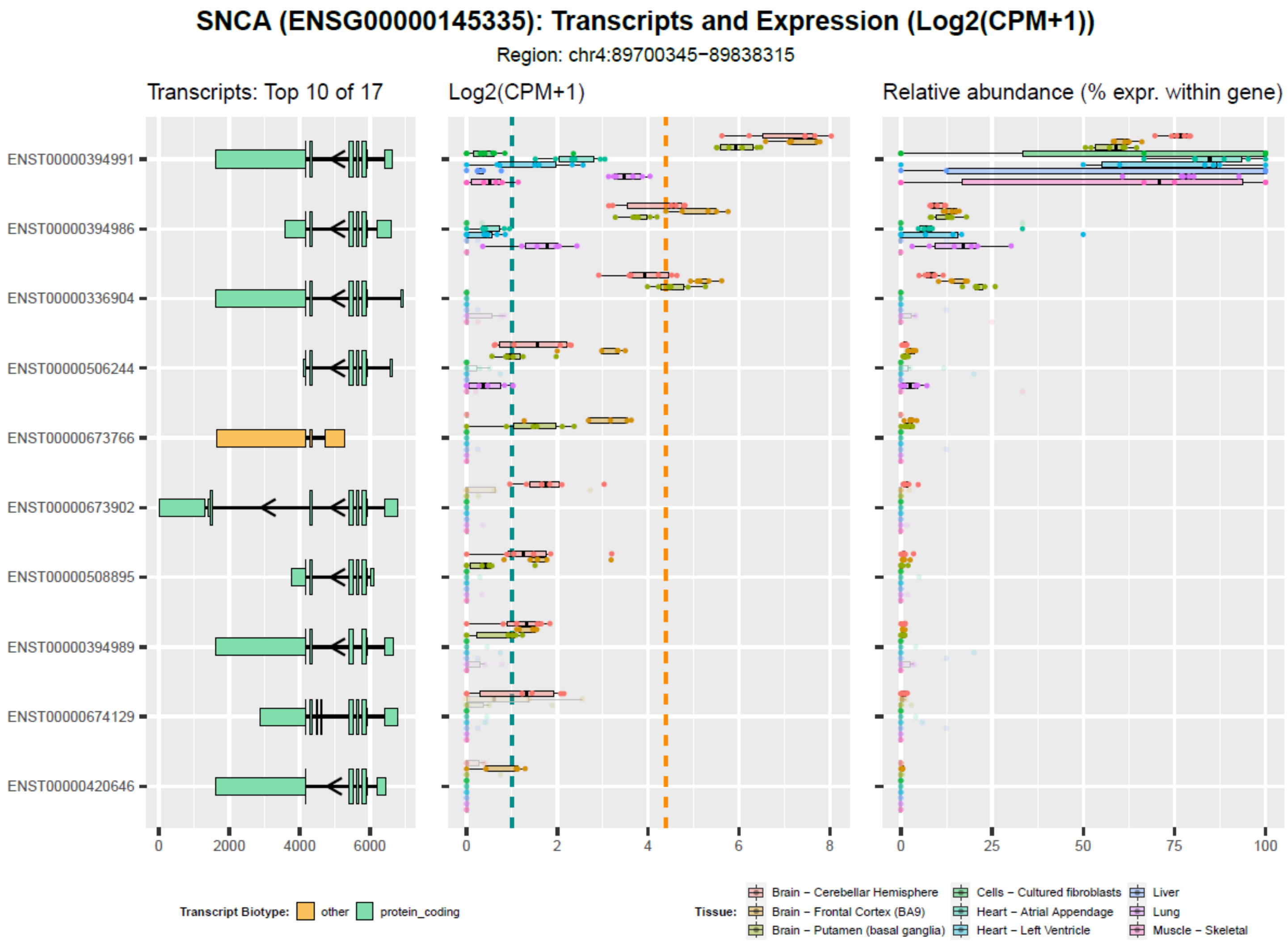

Supplemental Figure 11: SNCA isoforms, expression, and relative expression. Isoforms expressed above a median CPM > 1 are considered in our analyses. Faded box-plots represent tissues where the isoform did not pass our unique counts threshold (median unique counts ≥ 1), and therefore are not included in our analyses. Blue dashed line is CPM = 1 and orange dashed line is CPM = 20.

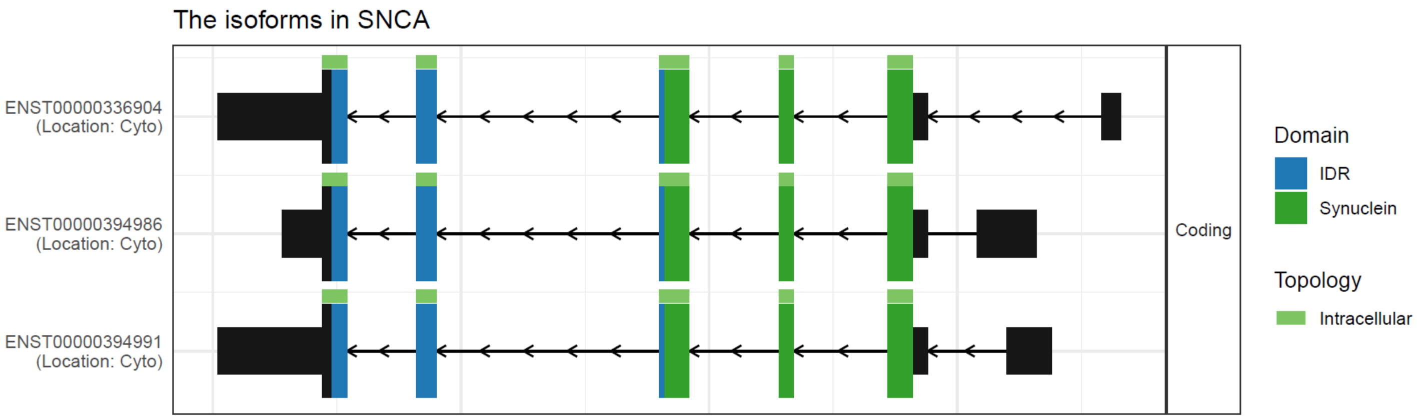

Supplemental Figure 12: SNCA IsoformSwitchAnalyzeR isoform plot output. The coding regions of these isoforms are the same.

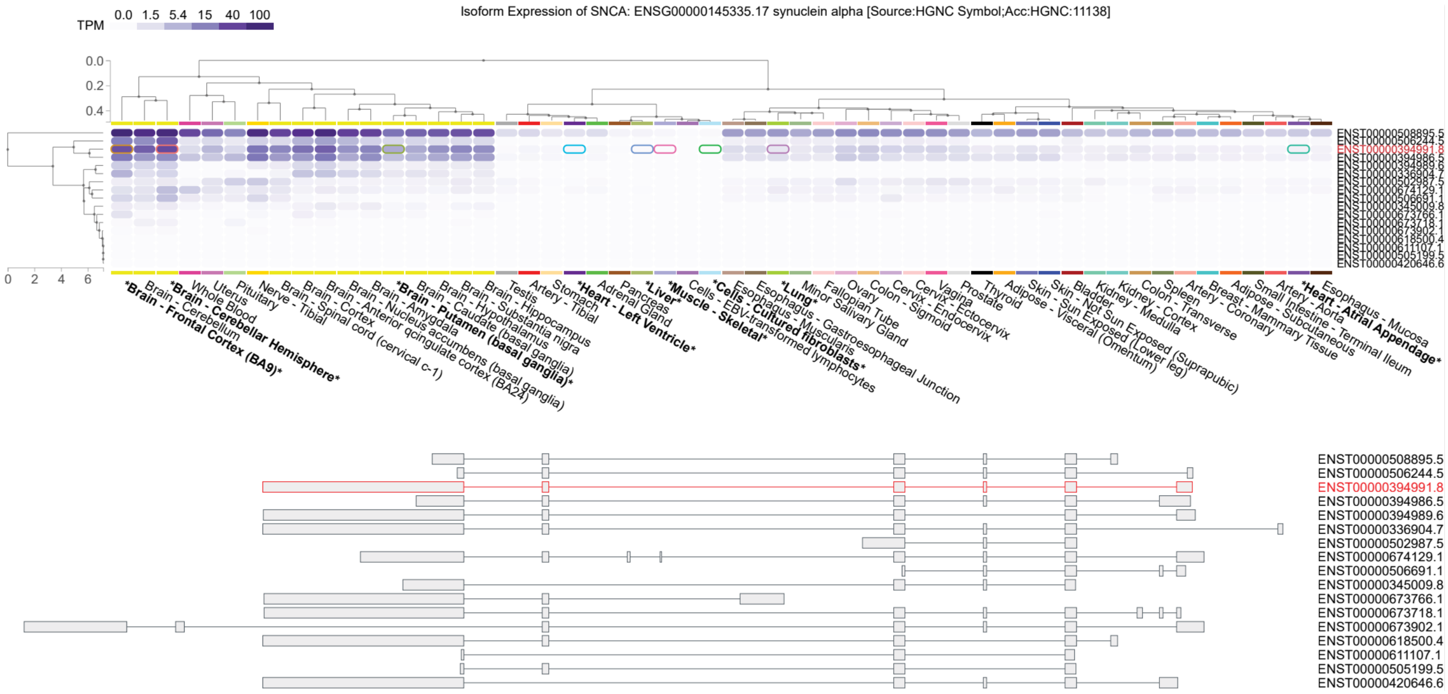

Supplemental Figure 13: SNCA GTEx short read isoform expression. Screenshot of short read GTEx isoform expression for *SNCA*. Tissues used in our long read data are highlighted, and the isoform most highly expressed in our data is highlighted.

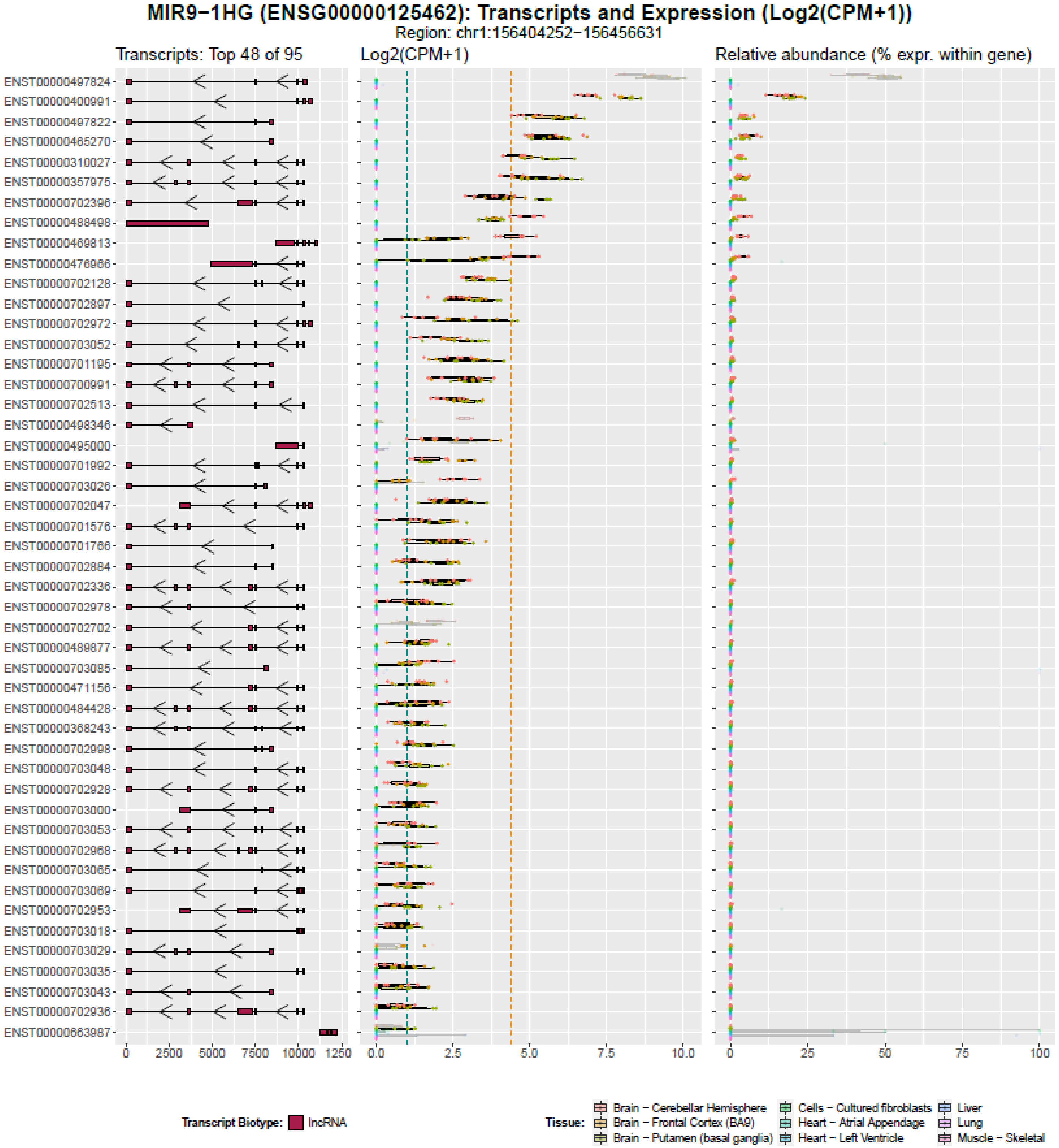

Supplemental Figure 14: MIR9-1HG isoforms, expression, and relative expression. Isoforms expressed above a median CPM > 1 are considered in our analyses. Faded box-plots represent tissues where the isoform did not pass our unique counts threshold (median unique counts ≥ 1), and therefore are not included in our analyses. Blue dashed line is CPM = 1 and orange dashed line is CPM = 20.

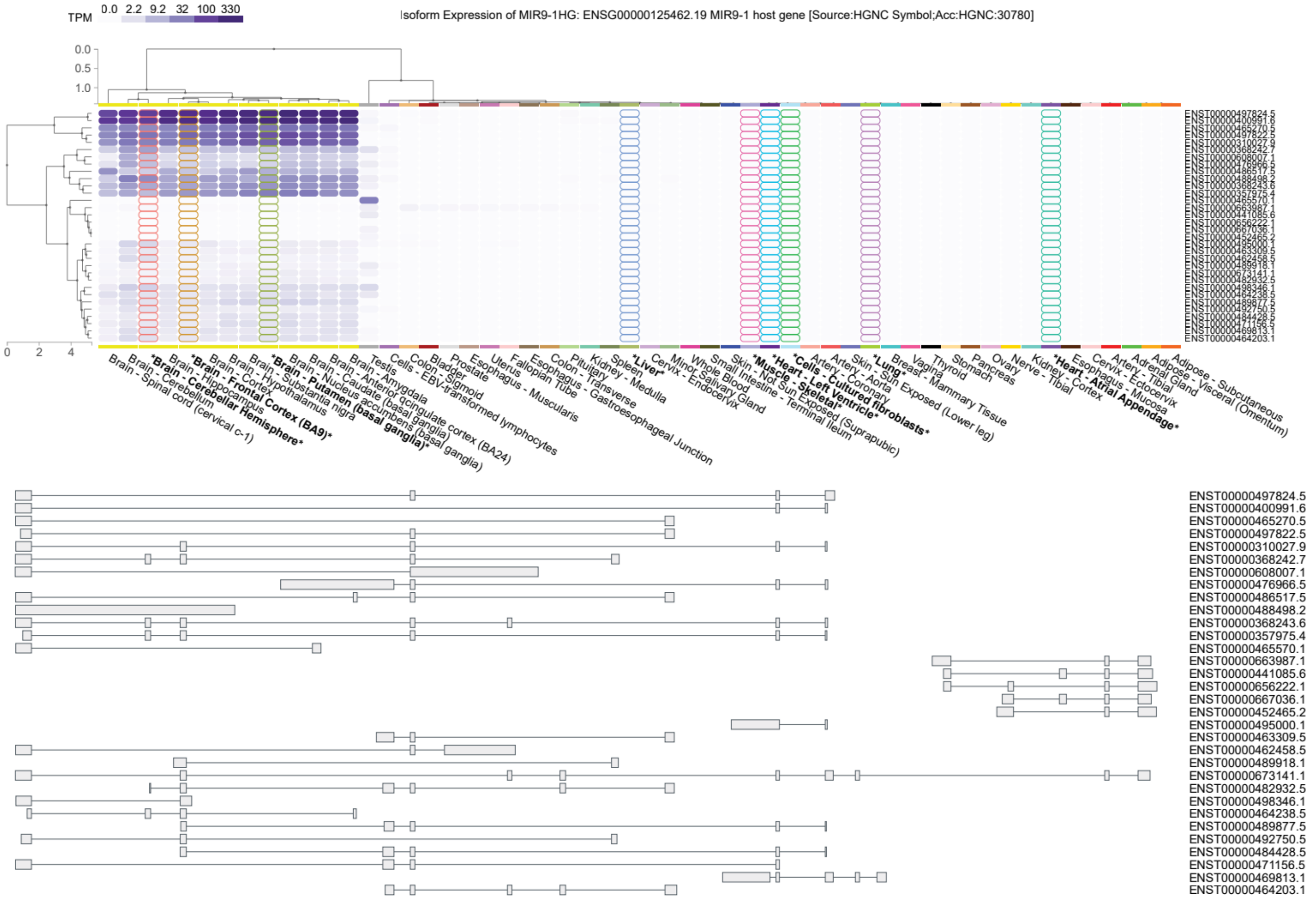

Supplemental Figure 15: MIR9-1HG GTEx short read isoform expression. Screenshot of short read GTEx isoform expression for *MIR9-1HG,* showing the isoforms expressed in the short read data. Tissues used in our long read data are highlighted.

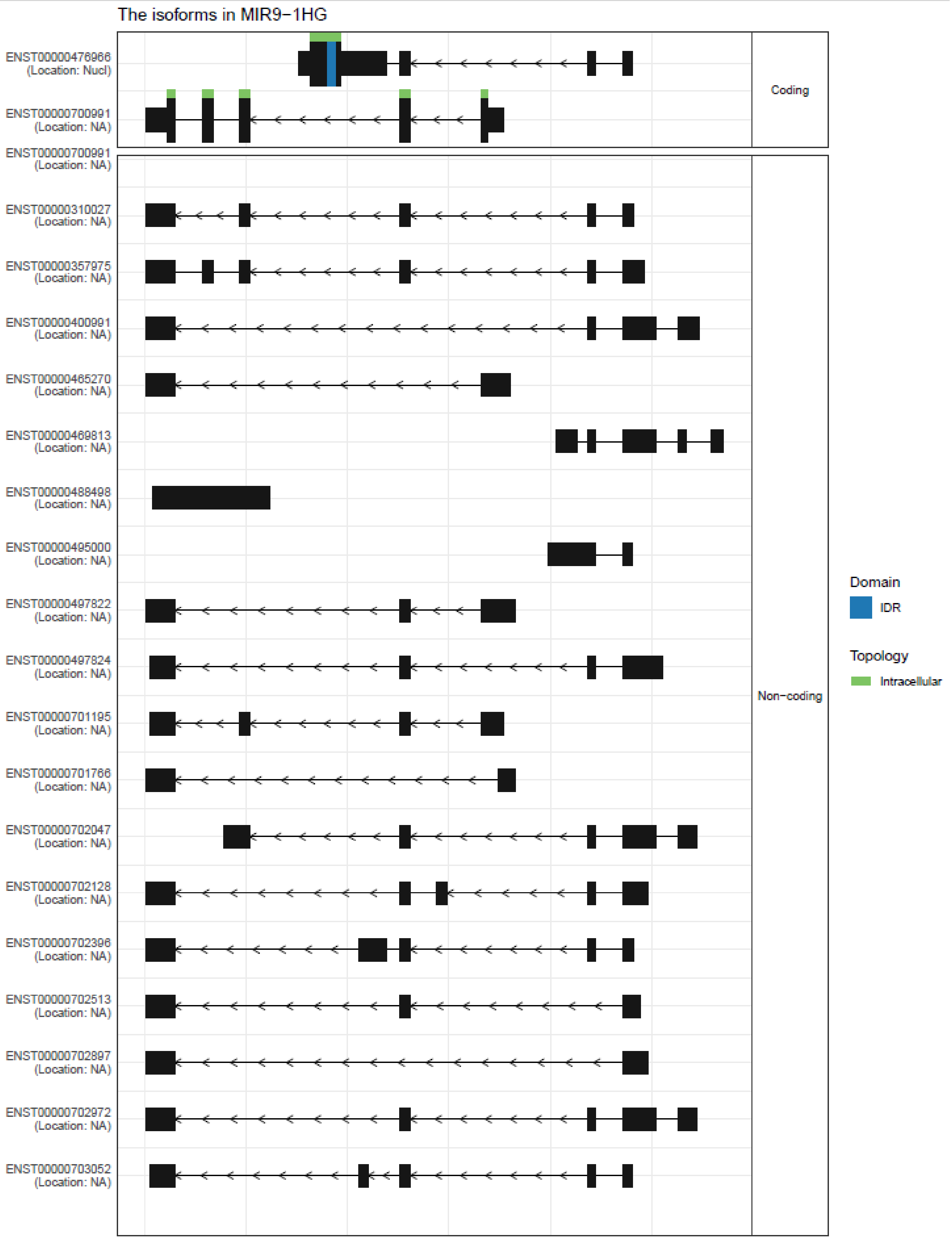

Supplemental Figure 16: MIR9-1HG IsoformSwitchAnalyzeR isoform plot output. One isoform is predicted to be protein coding.

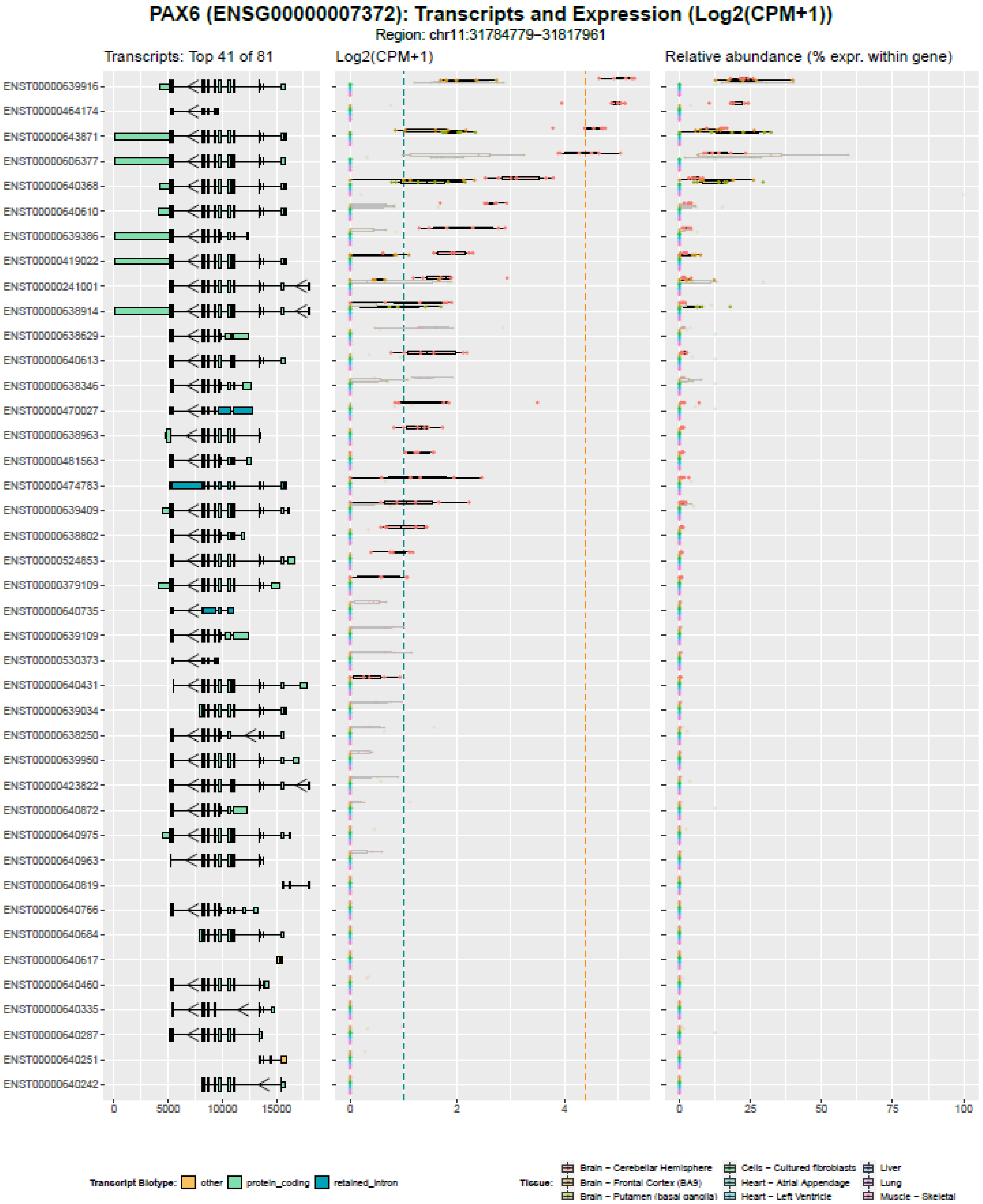

Supplemental Figure 17: PAX6 isoforms, expression, and relative abundance. Isoforms expressed above a median CPM > 1 are considered in our analyses. Faded box-plots represent tissues where the isoform did not pass our unique counts threshold (median unique counts ≥ 1), and therefore are not included in our analyses. Blue dashed line is CPM = 1 and orange dashed line is CPM = 20. Relative abundance for each isoform seems similar across the brain tissues.

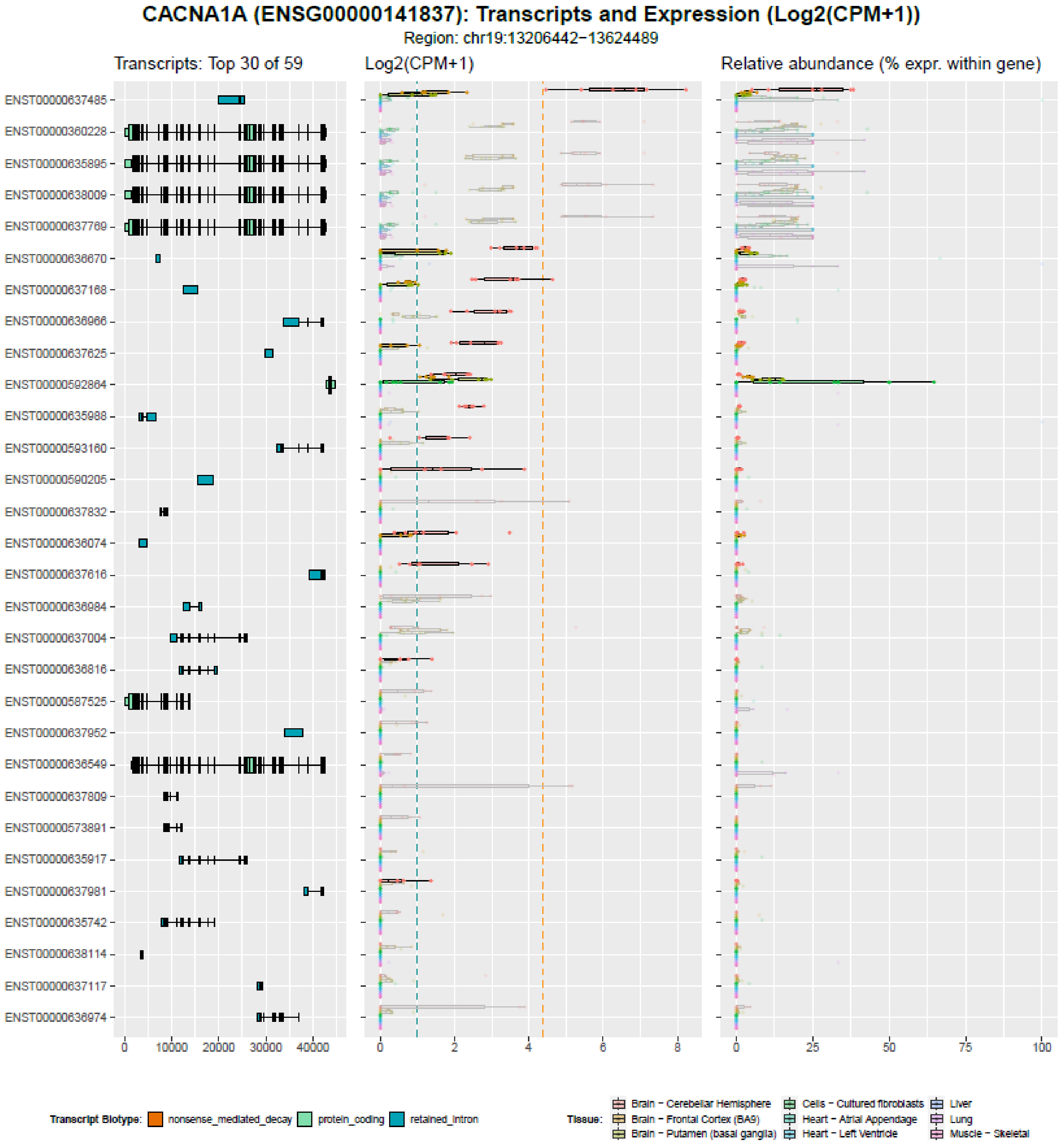

Supplemental Figure 18: CACNA1A isoforms, expression, and relative expression. Isoforms expressed above a median CPM > 1 are considered in our analyses. Faded box-plots represent tissues where the isoform did not pass our unique counts threshold (median unique counts ≥ 1), and therefore are not included in our analyses. Blue dashed line is CPM = 1 and orange dashed line is CPM = 20. Cerebellar hemisphere is expressing the greatest number of isoforms.

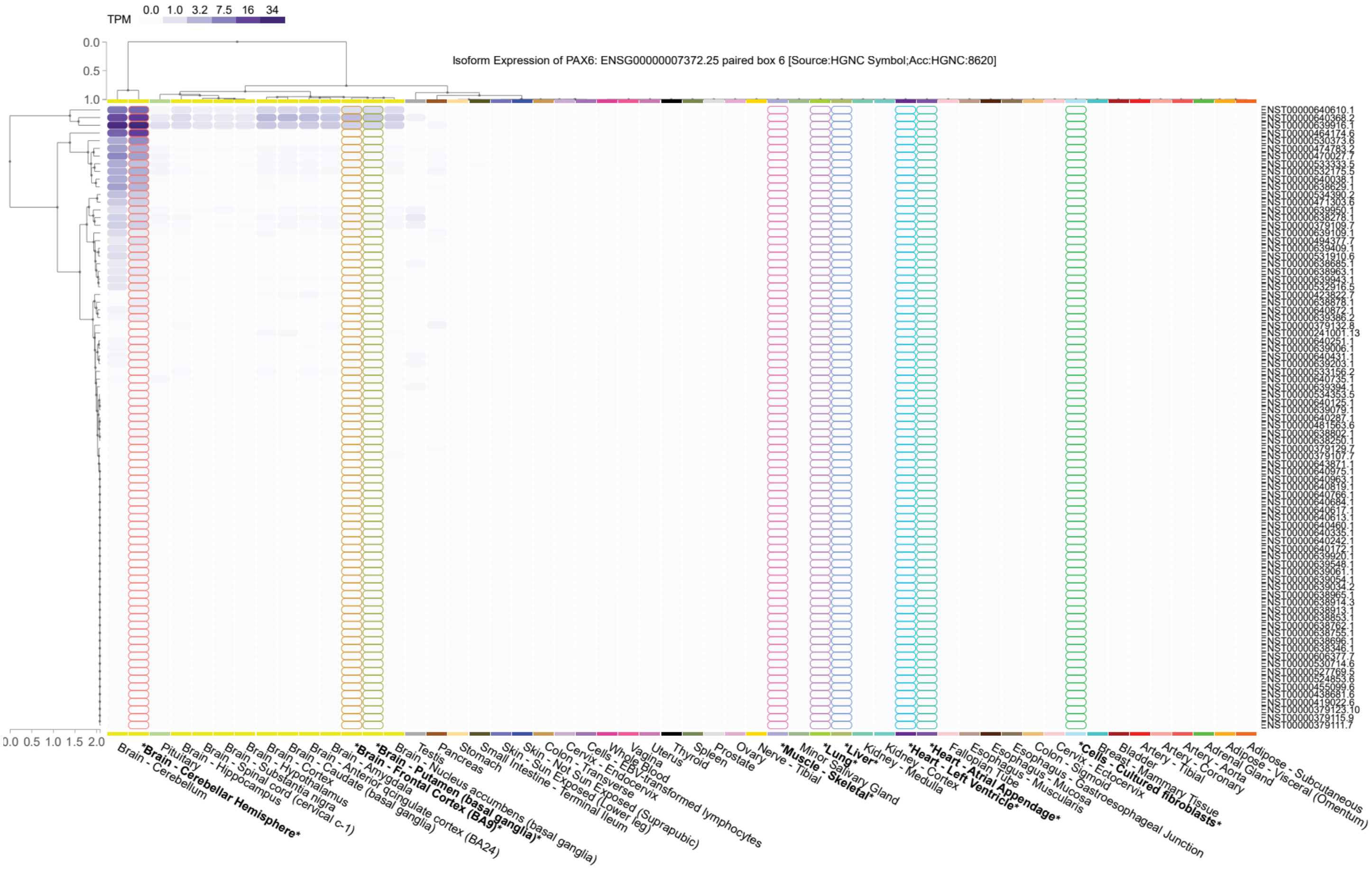

Supplemental Figure 19: PAX6 GTEx short read isoform expression. Screenshot of short read GTEx isoform expression for *PAX6*. Tissues used in our long read data are highlighted.

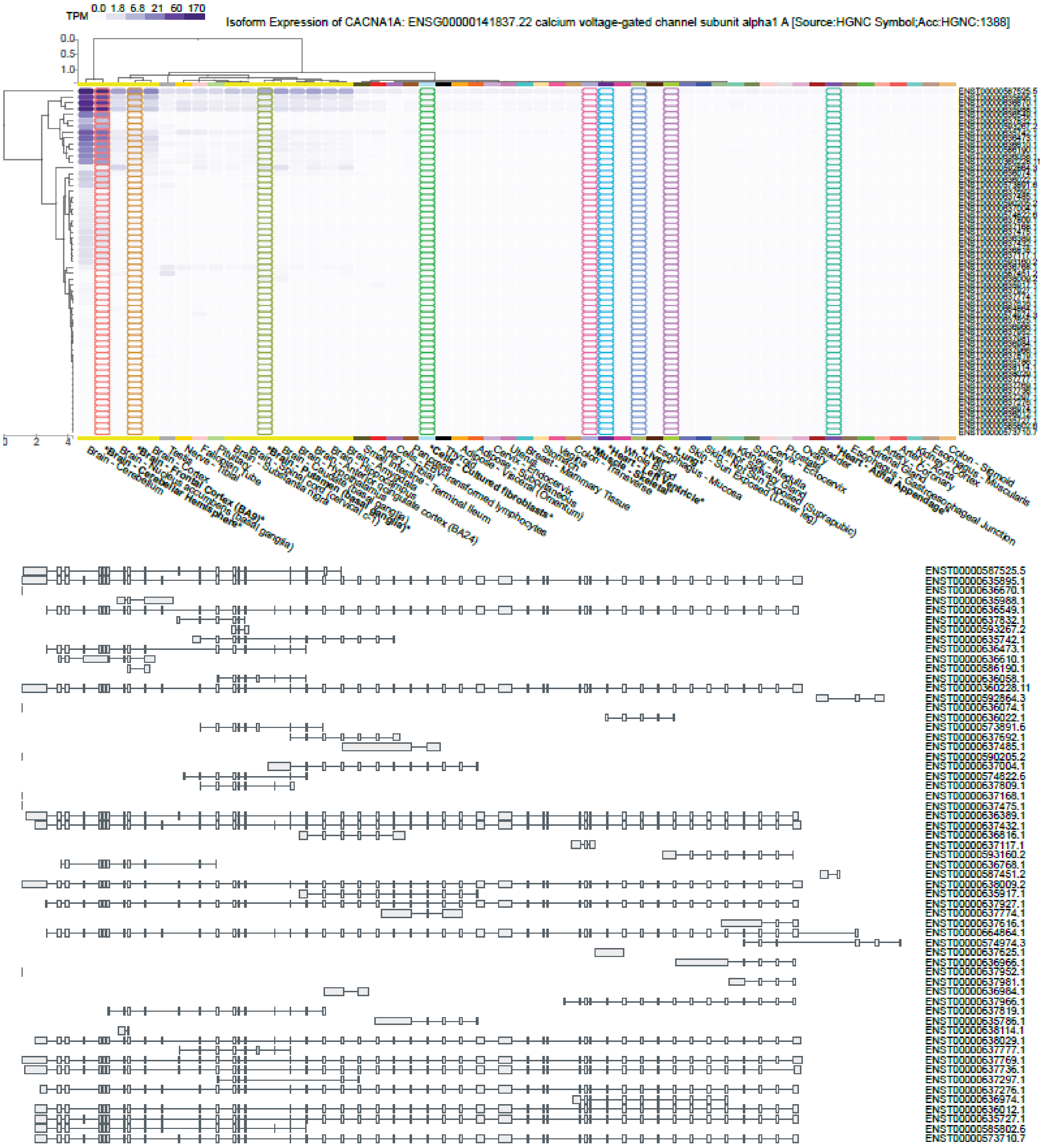

Supplemental Figure 20: CACNA1A GTEx short read isoform expression. Screenshot of short read GTEx isoform expression for *CACNA1A*. Tissues used in our long read data are highlighted.

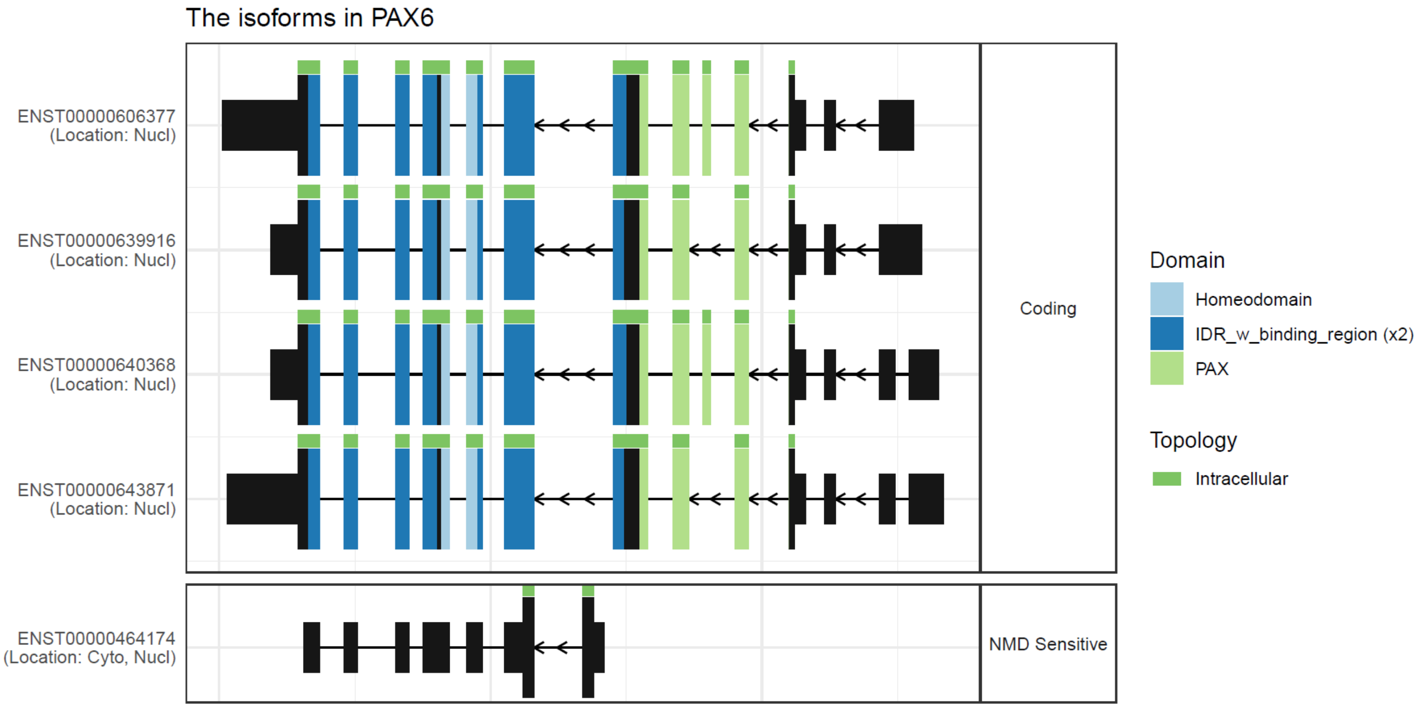

Supplemental Figure 21: PAX6 IsoformSwitchAnalyzeR isoform plot output.

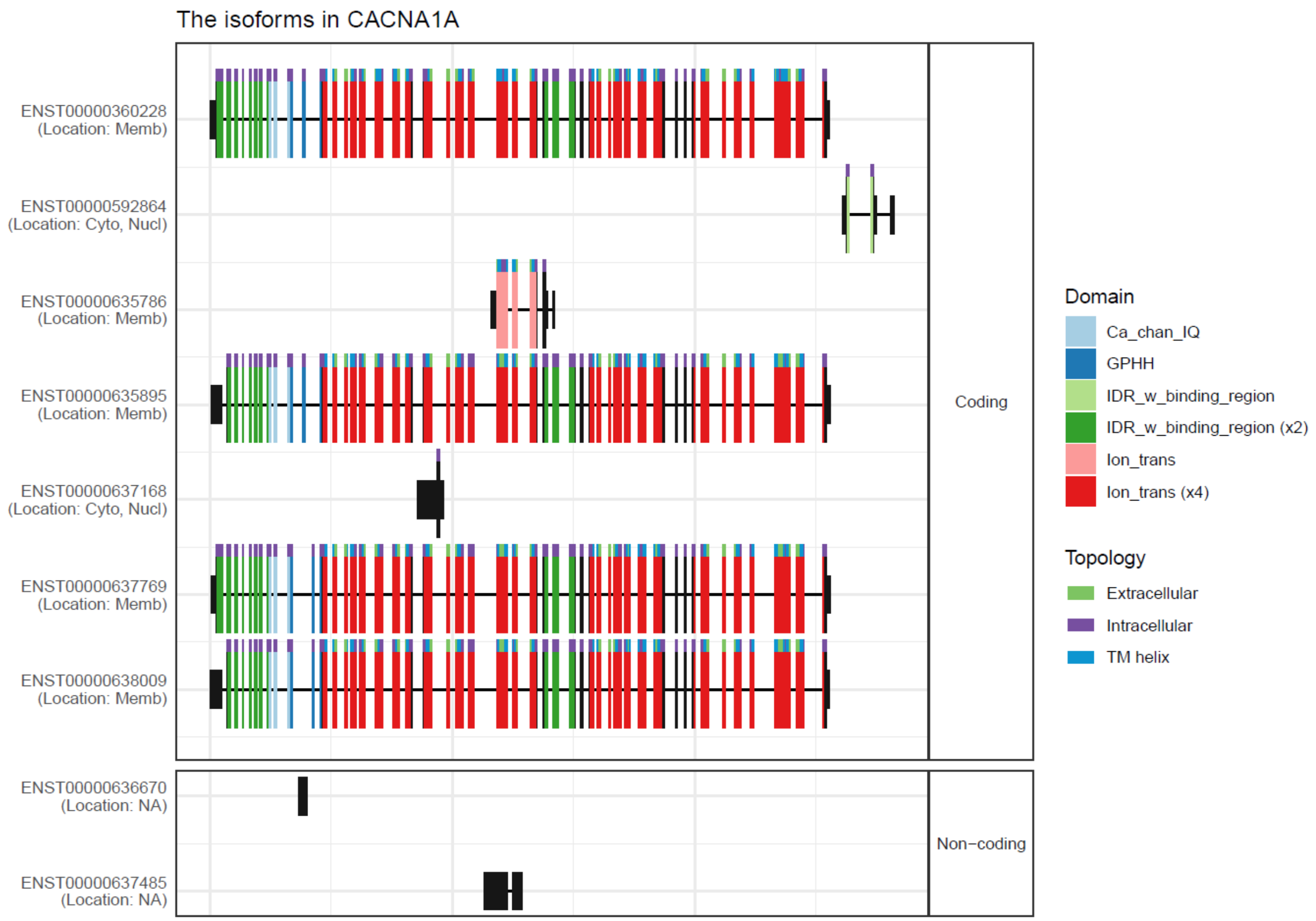

Supplemental Figure 22: CACNA1A IsoformSwitchAnalyzeR isoform plot output.

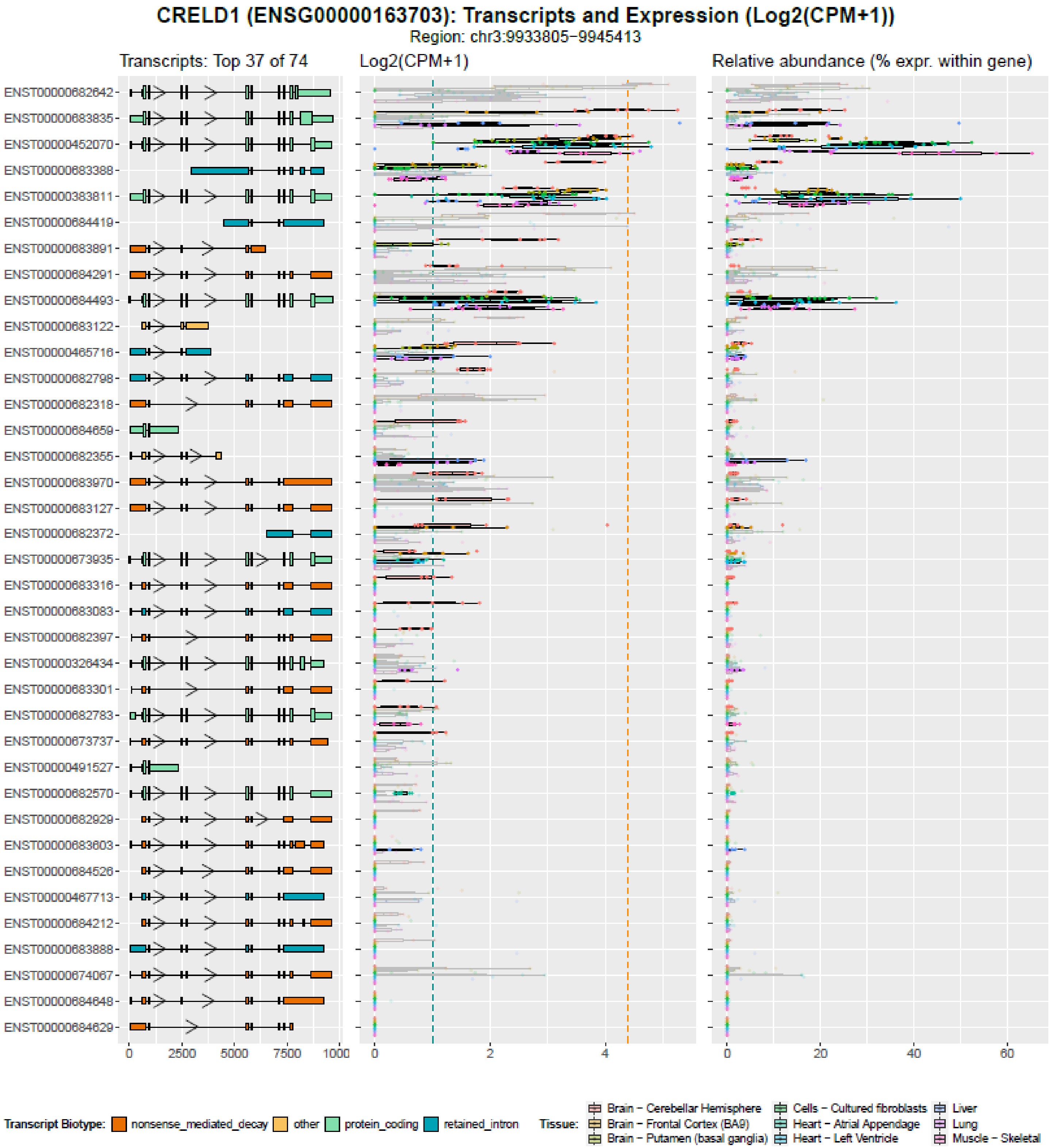

Supplemental Figure 23: CRELD1 isoforms, expression, and relative expression. Isoforms expressed above a median CPM > 1 are considered in our analyses. Faded box-plots represent tissues where the isoform did not pass our unique counts threshold (median unique counts ≥ 1), and therefore are not included in our analyses. Blue dashed line is CPM = 1 and orange dashed line is CPM = 20. Cerebellar hemisphere expresses the greatest number of isoforms.

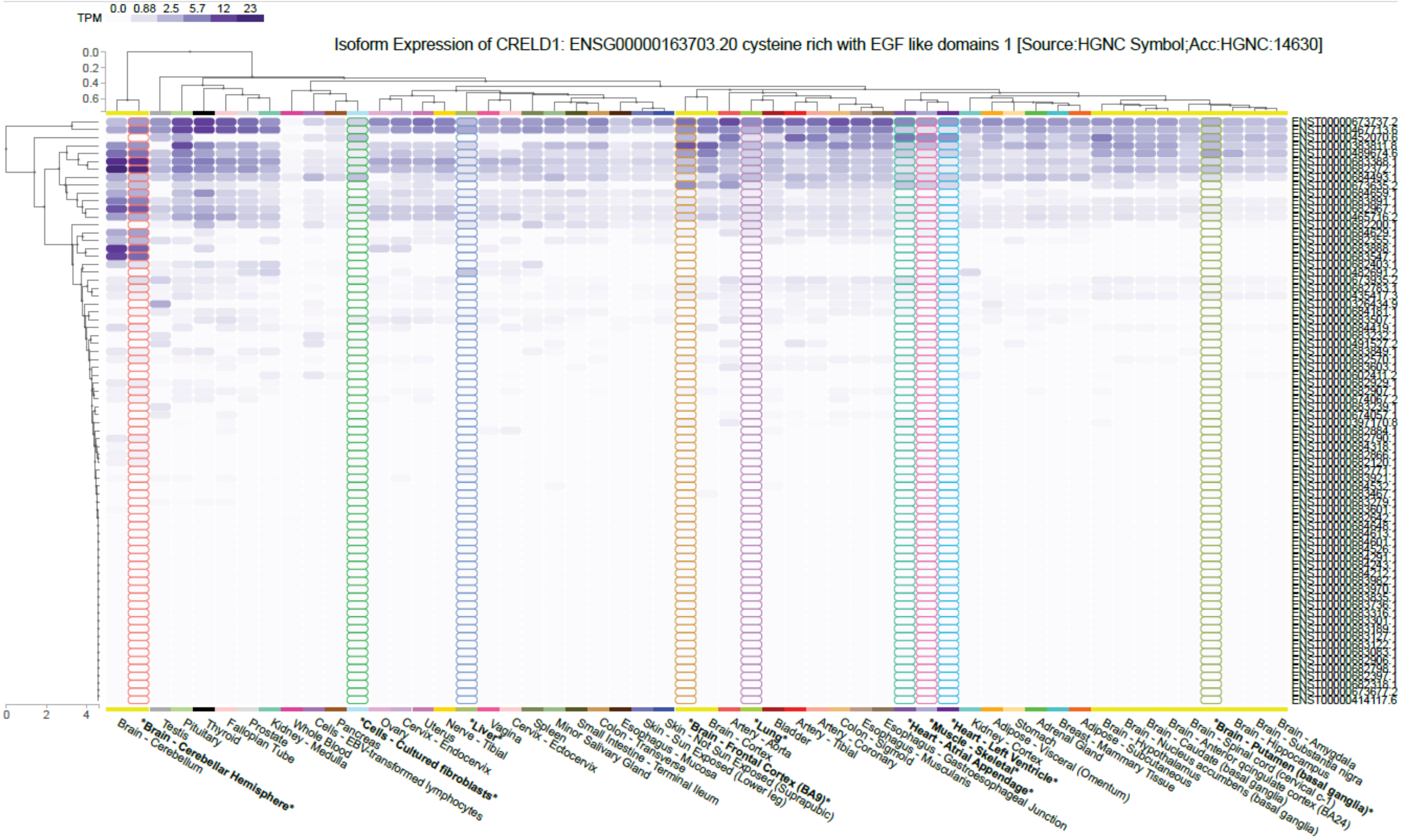

Supplemental Figure 24: CRELD1 GTEx short read isoform expression. Screenshot of short read GTEx isoform expression for *CRELD1*. Tissues used in our long read data are highlighted. Cerebellar Hemisphere expresses *CRELD1* more than heart.

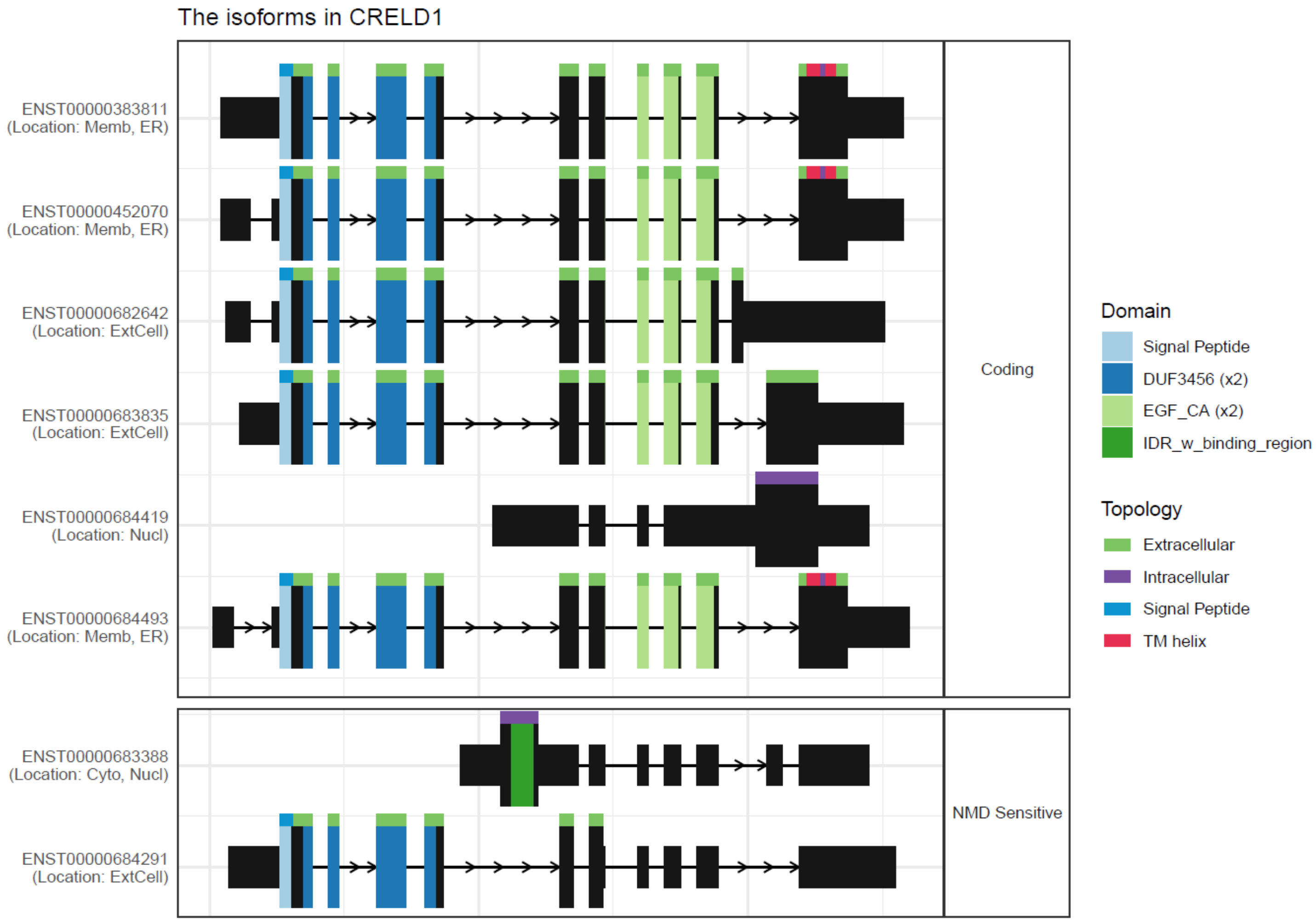

Supplemental Figure 25: CRELD1 IsoformSwitchAnalyzeR isoform plot output.

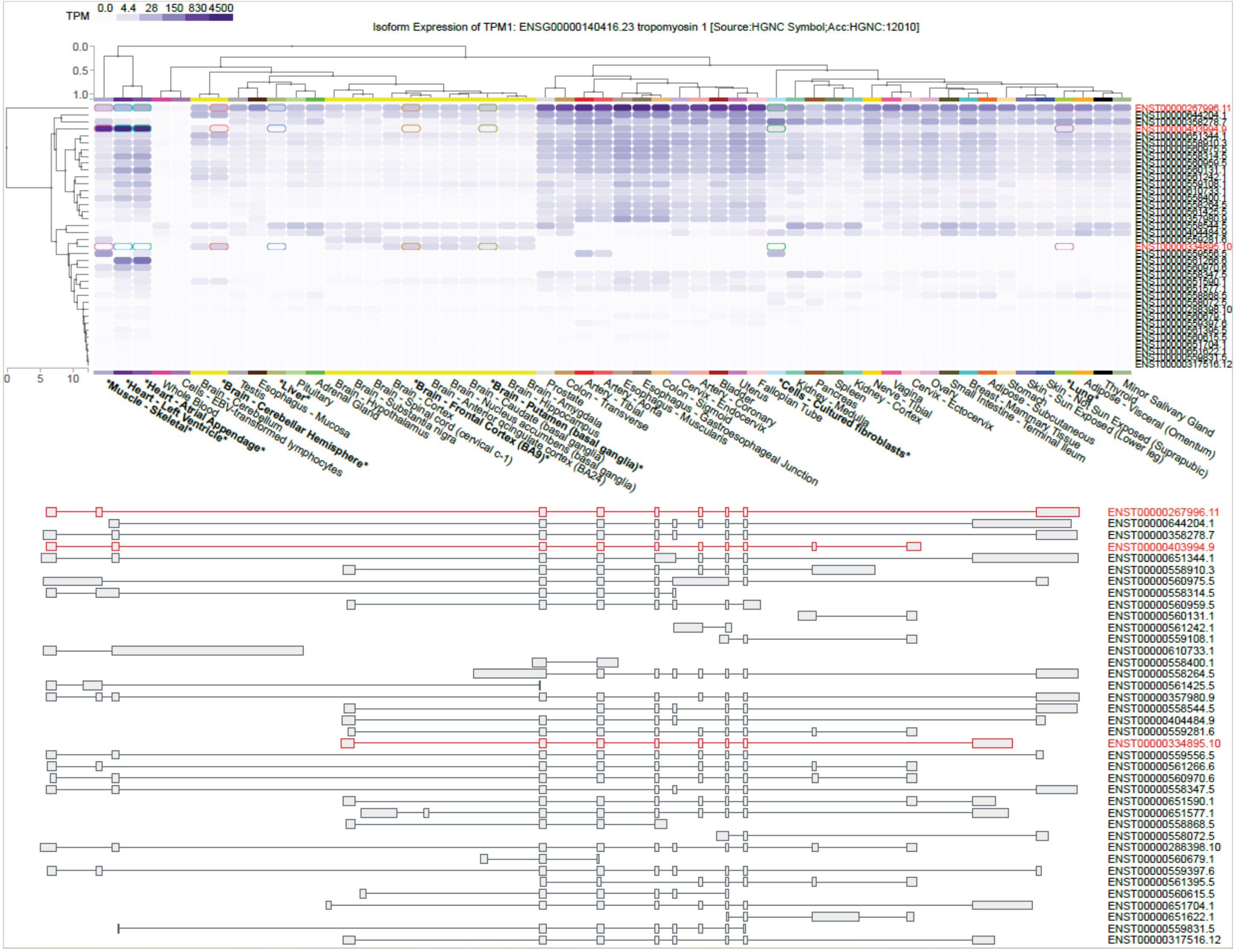

Supplemental Figure 26: TPM1 GTEx short read isoform expression. Screenshot of short read GTEx isoform expression for *TPM1*. Tissues used in our long read data are highlighted, and the isoforms we highlight in our text are highlighted here.

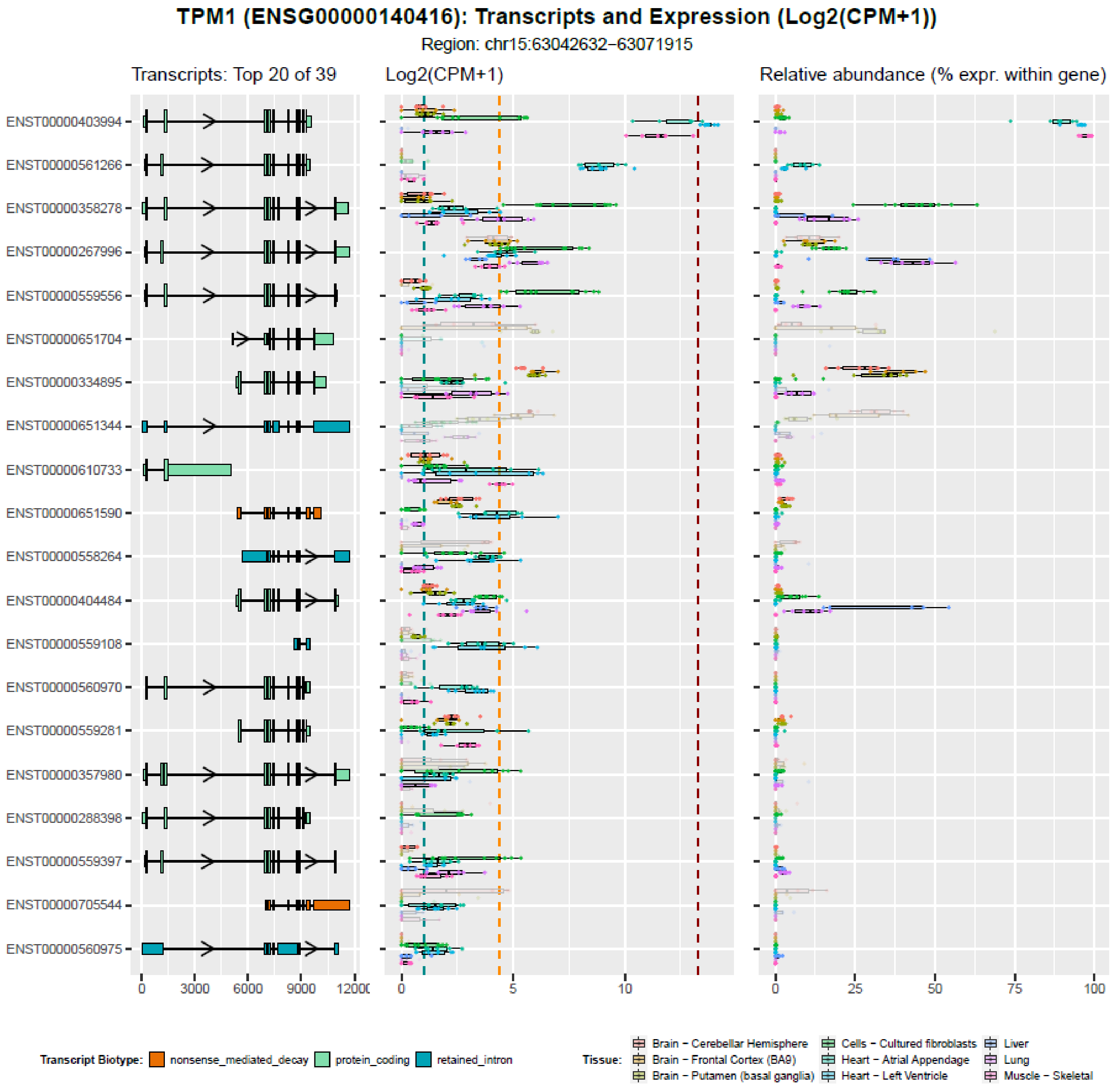

Supplemental Figure 27: TPM1 isoforms, expression, and relative expression. Isoforms expressed above a median CPM > 1 are considered in our analyses. Faded box-plots represent tissues where the isoform did not pass our unique counts threshold (median unique counts ≥ 1), and therefore are not included in our analyses. Blue dashed line is CPM = 1, orange dashed line is CPM = 20, and red dashed line is CPM = 10000.

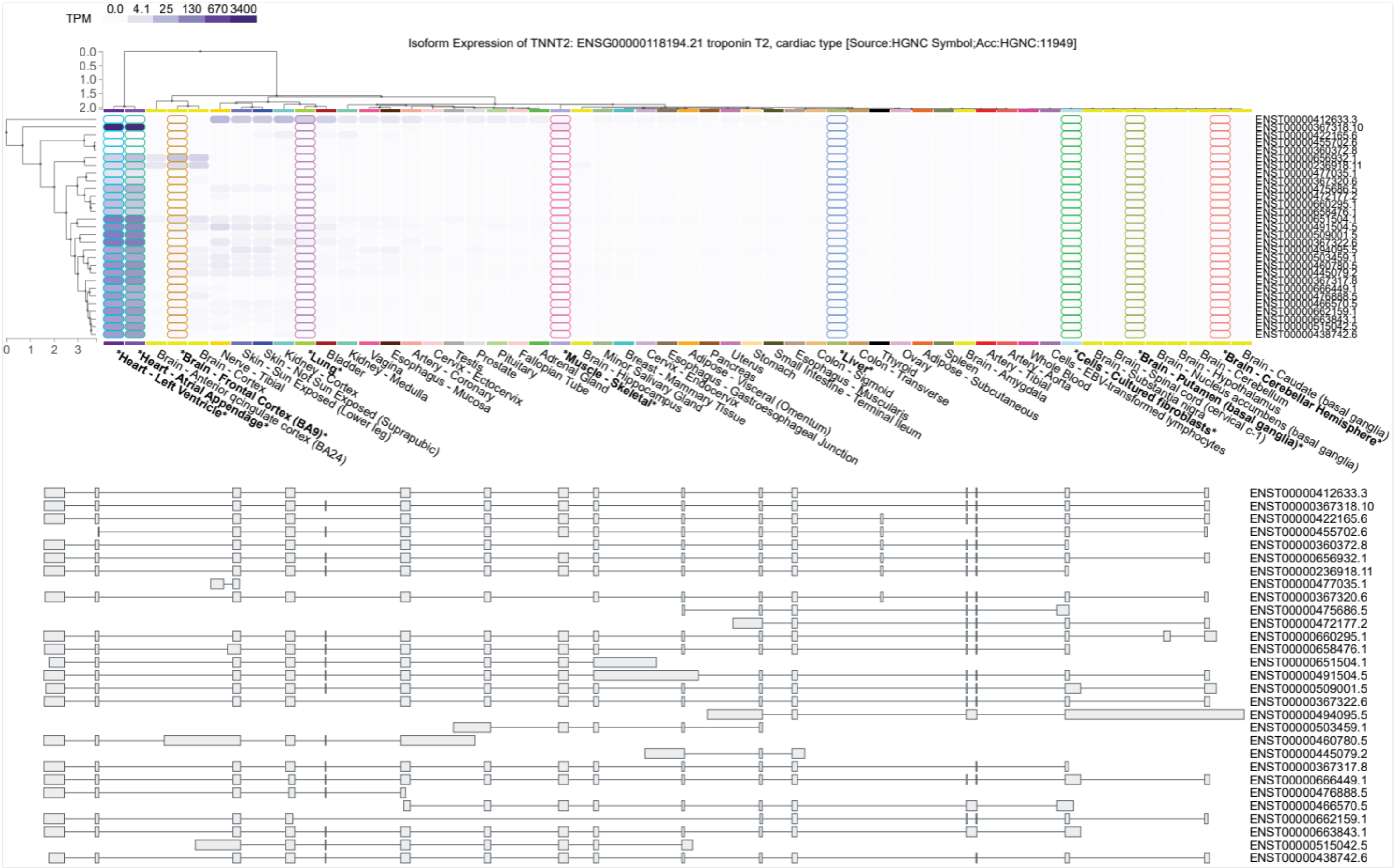

Supplemental Figure 28: TNNT2 GTEx short read isoform expression. Screenshot of short read GTEx isoform expression for *TNNT2*. Tissues used in our long read data are highlighted.

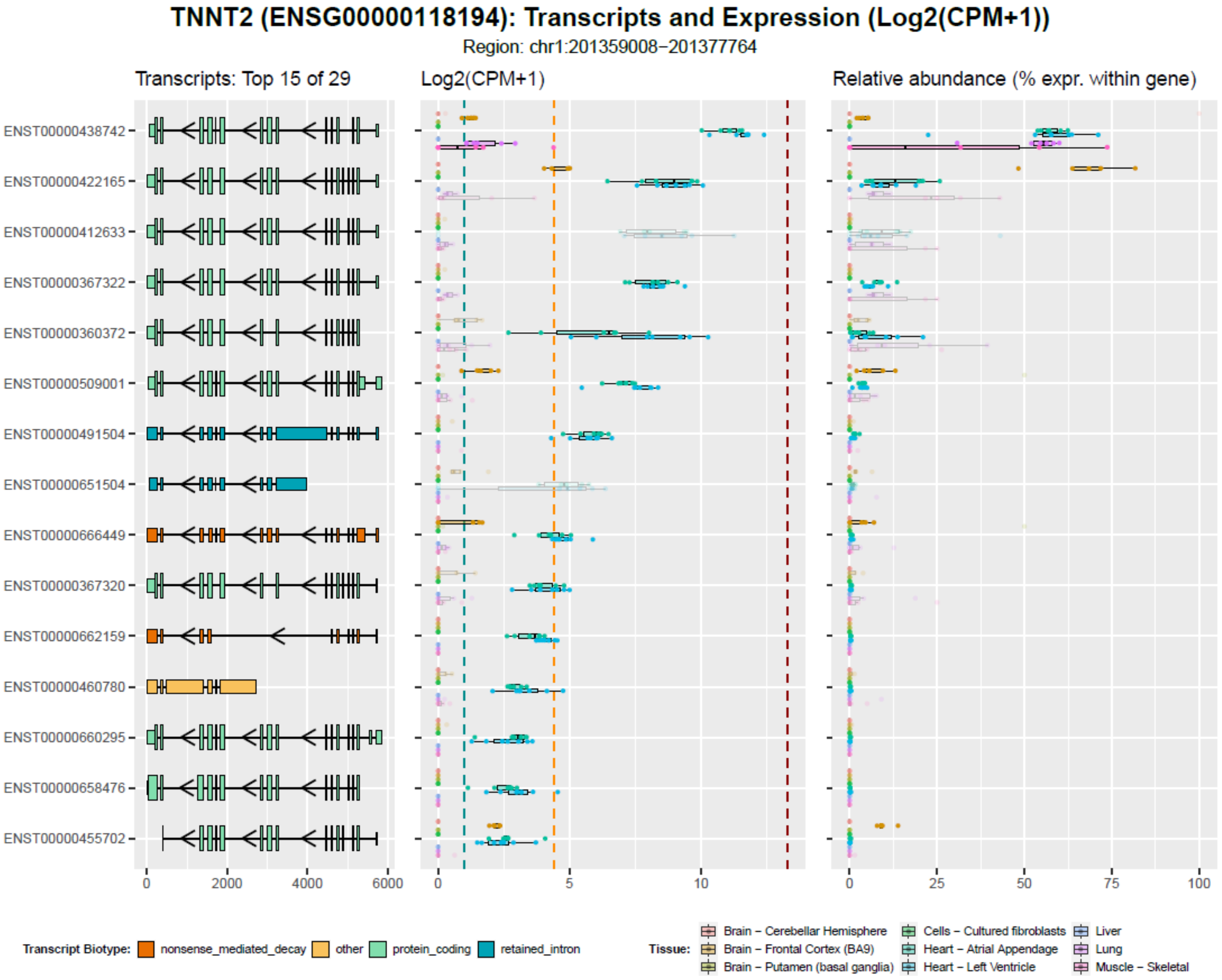

Supplemental Figure 29: TNNT2 isoforms, expression, and relative expression. Isoforms expressed above a median CPM > 1 are considered in our analyses. Faded box-plots represent tissues where the isoform did not pass our unique counts threshold (median unique counts ≥ 1), and therefore are not included in our analyses. Blue dashed line is CPM = 1, orange dashed line is CPM = 20, and red dashed line is CPM = 10000.

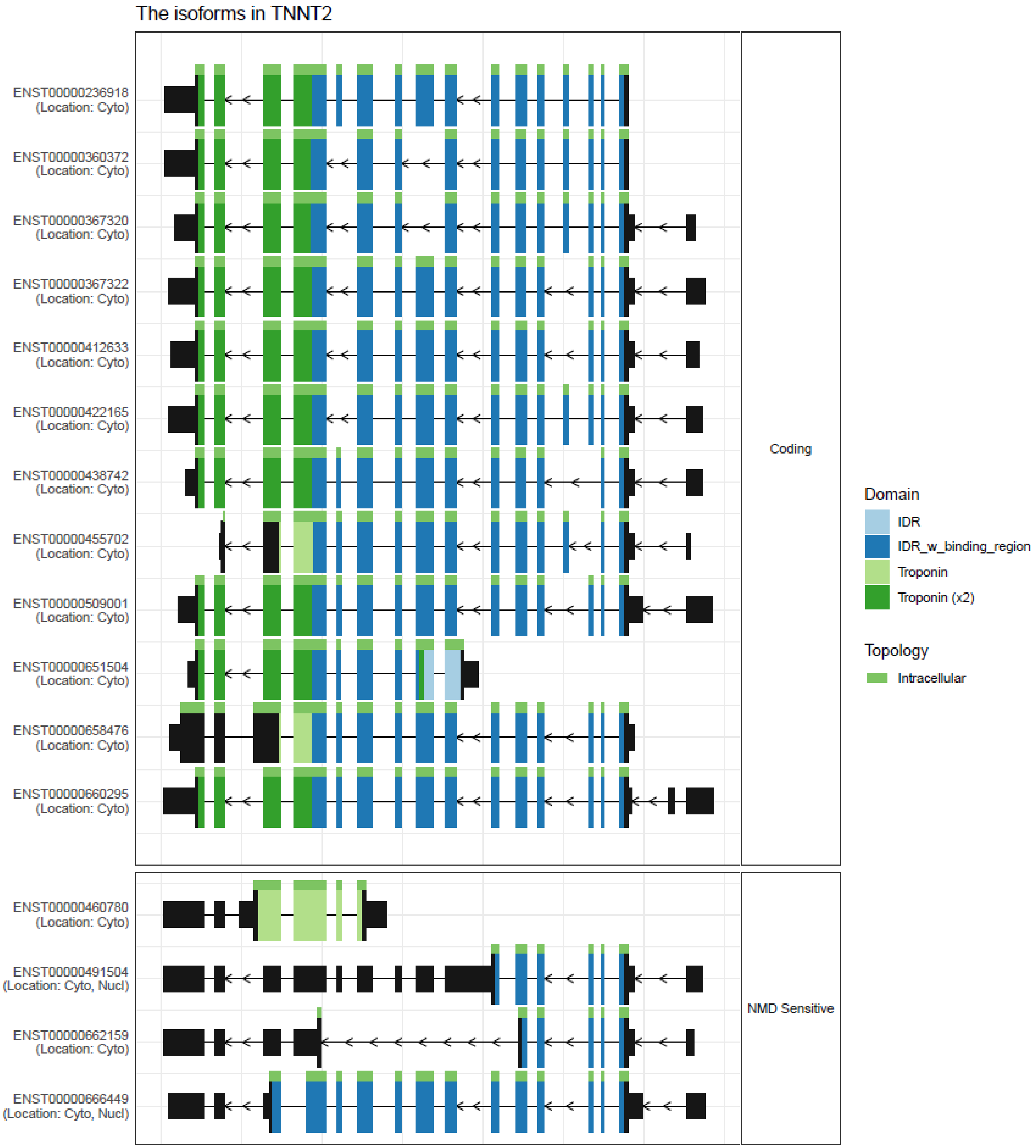

Supplemental Figure 30: TNNT2 IsoformSwitchAnalyzeR isoform plot output. ENST00000438742 is the most highly expressed isoform in Brain – frontal cortex while ENST00000422165 is the most highly expressed in heart tissues. The two isoforms differ in their exons and UTRs.

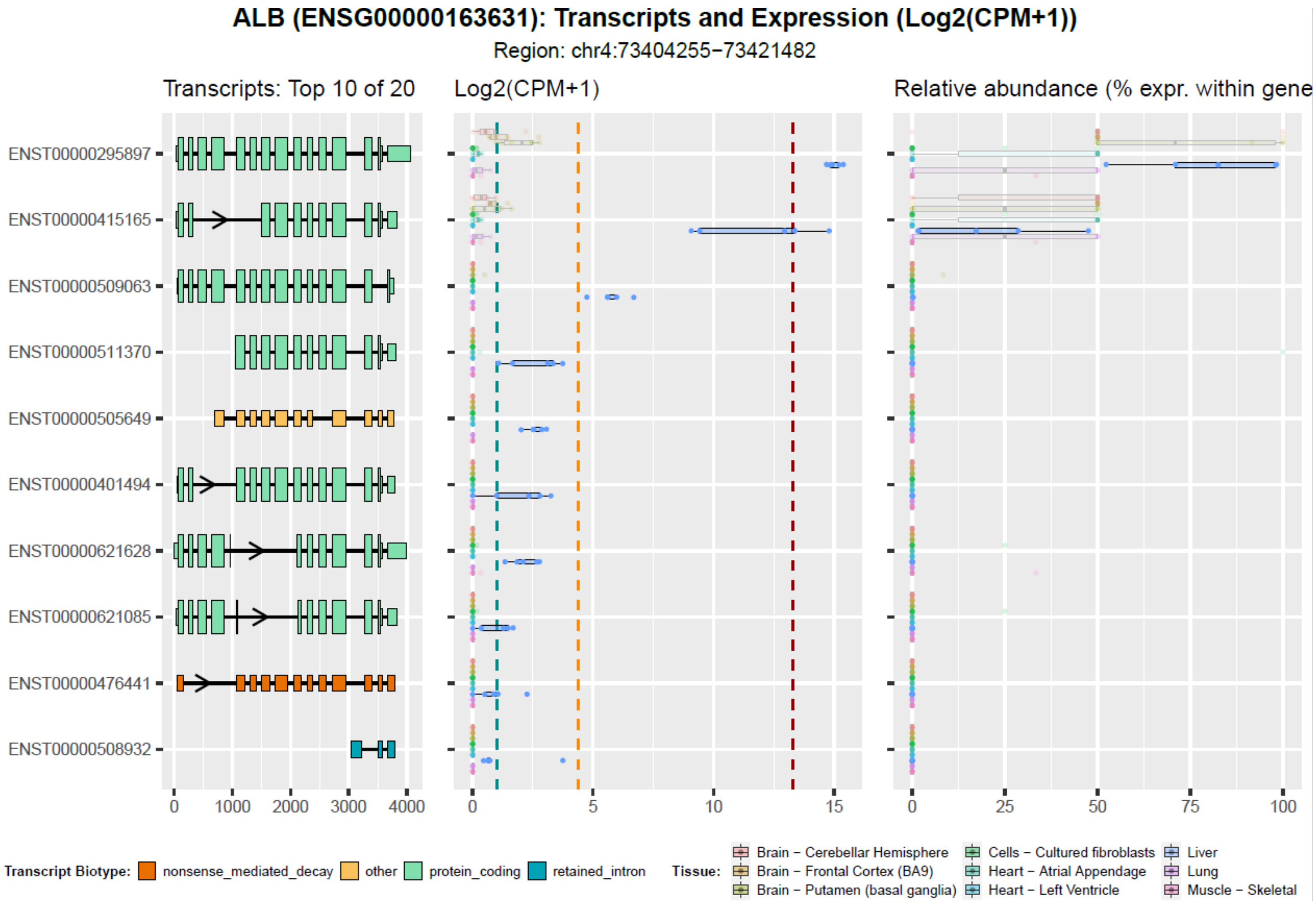

Supplemental Figure 31: ALB isoforms, expression, and relative expression. Isoforms expressed above a median CPM > 1 are considered in our analyses. Faded box-plots represent tissues where the isoform did not pass our unique counts threshold (median unique counts ≥ 1), and therefore are not included in our analyses. Blue dashed line is CPM = 1, orange dashed line is CPM = 20, and red dashed line is CPM = 10000.

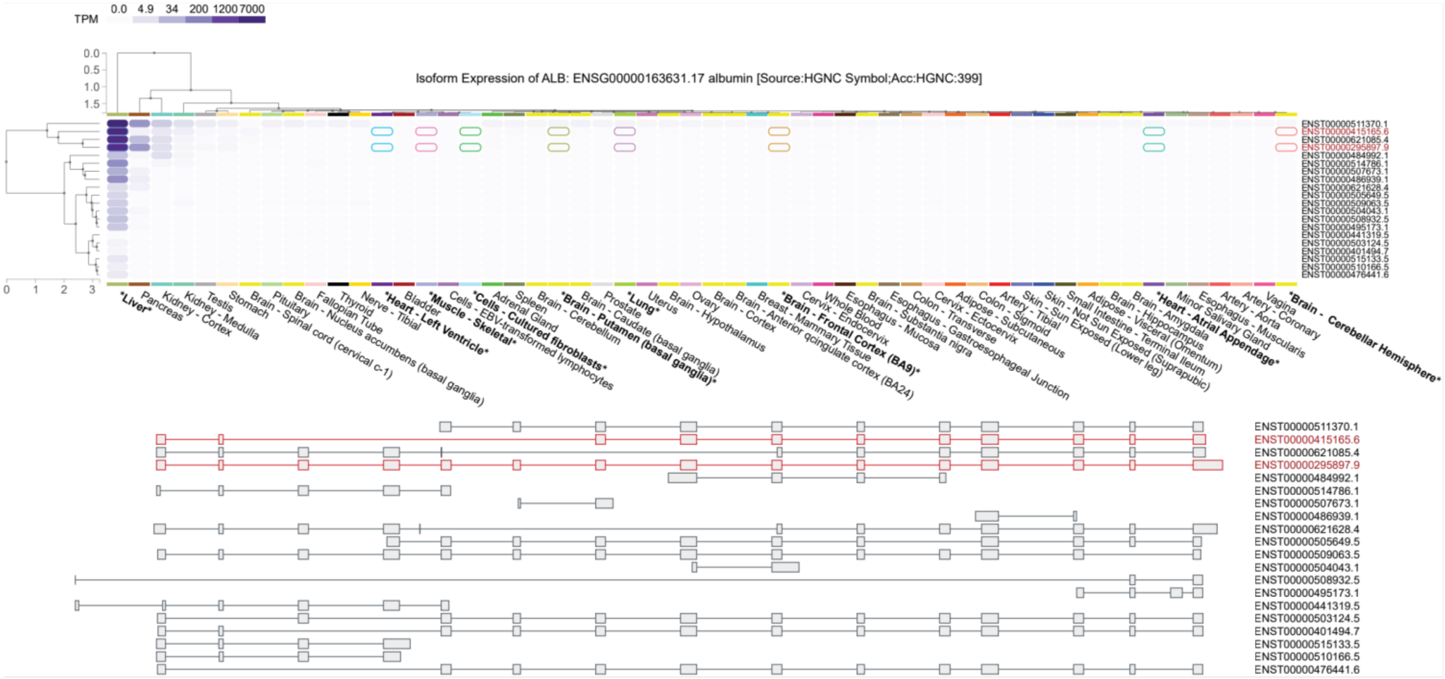

Supplemental Figure 32: ALB GTEx short read isoform expression. Screenshot of short read GTEx isoform expression for *ALB*. Tissues used in our long read data are highlighted, and isoforms we highlight are highlighted here.

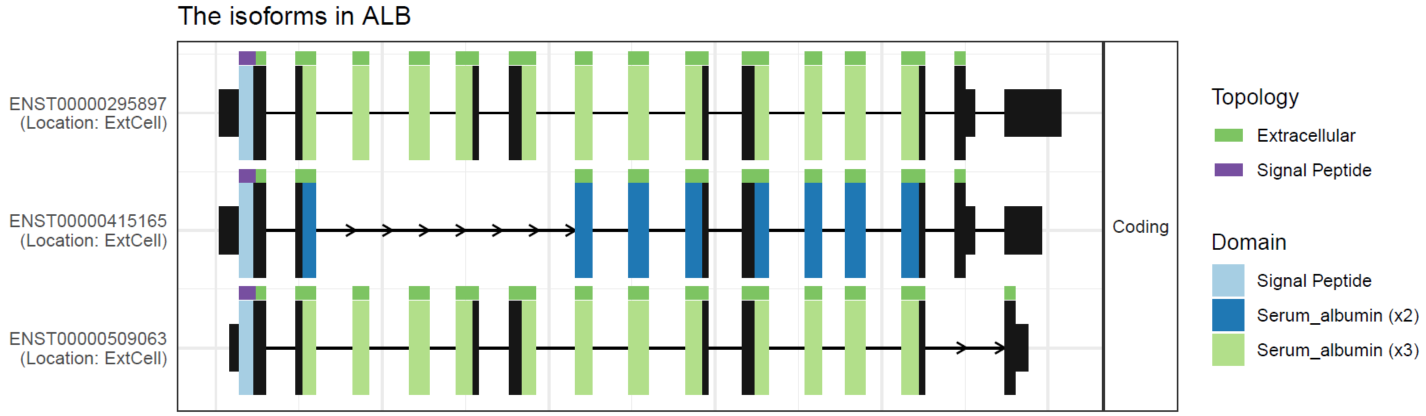

Supplemental Figure 33: ALB IsoformSwitchAnalyzeR isoform plot output.

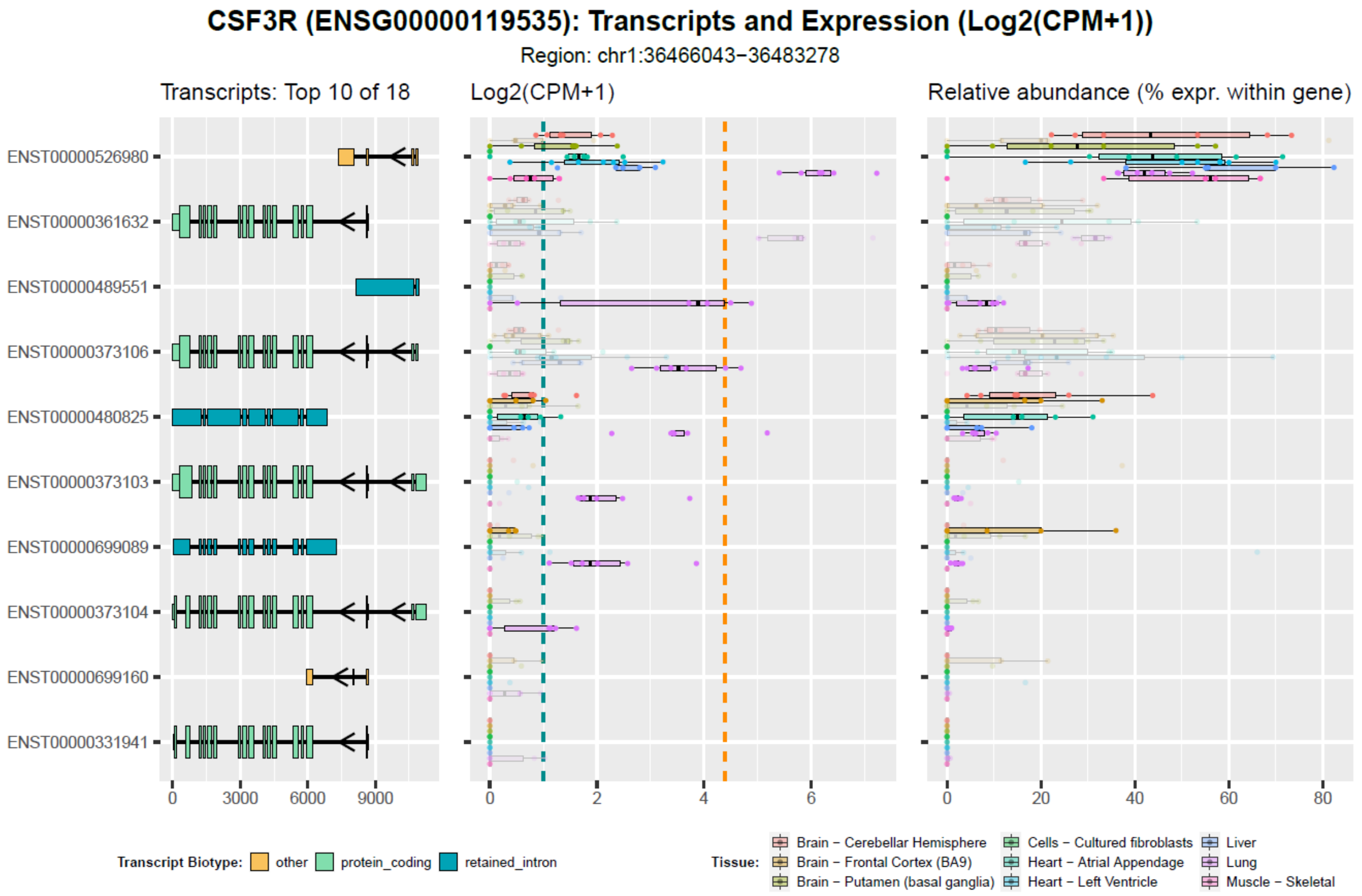

Supplemental Figure 34: CSF3R isoforms, expression, and relative expression. Isoforms expressed above a median CPM > 1 are considered in our analyses. Faded box-plots represent tissues where the isoform did not pass our unique counts threshold (median unique counts ≥ 1), and therefore are not included in our analyses. Blue dashed line is CPM = 1 and orange dashed line is CPM = 20.

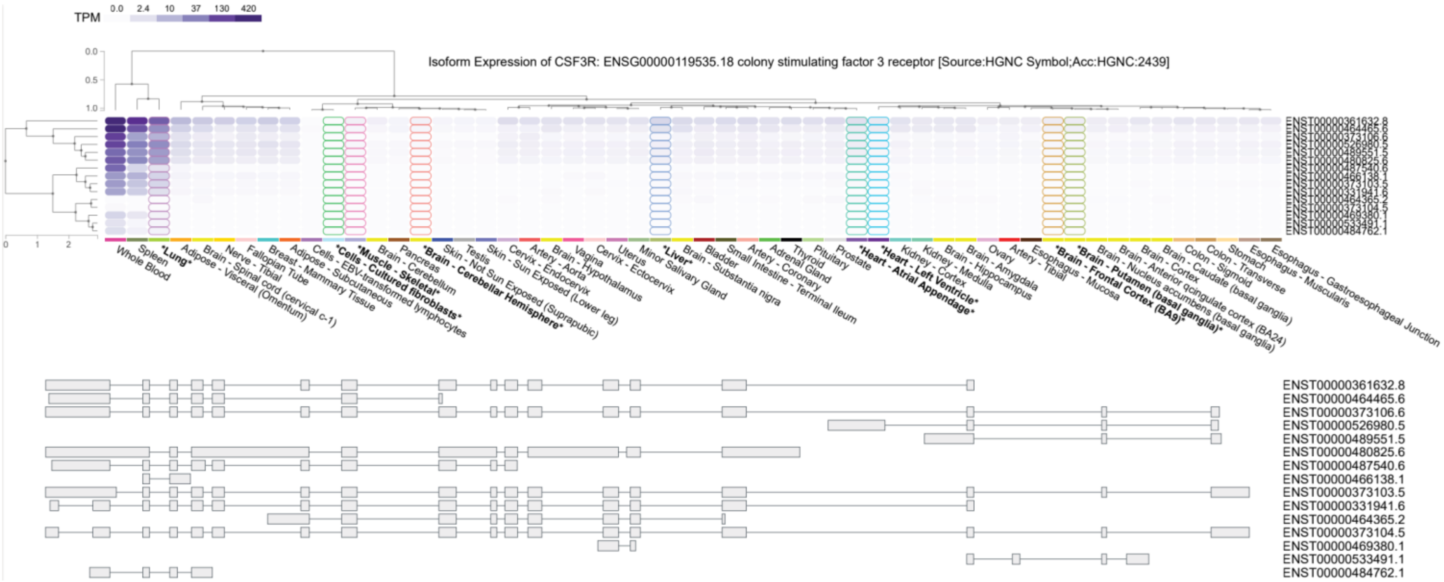

Supplemental Figure 35: CSF3R GTEx short read isoform expression. Screenshot of short read GTEx isoform expression for *CSF3R*. Tissues used in our long read data are highlighted.

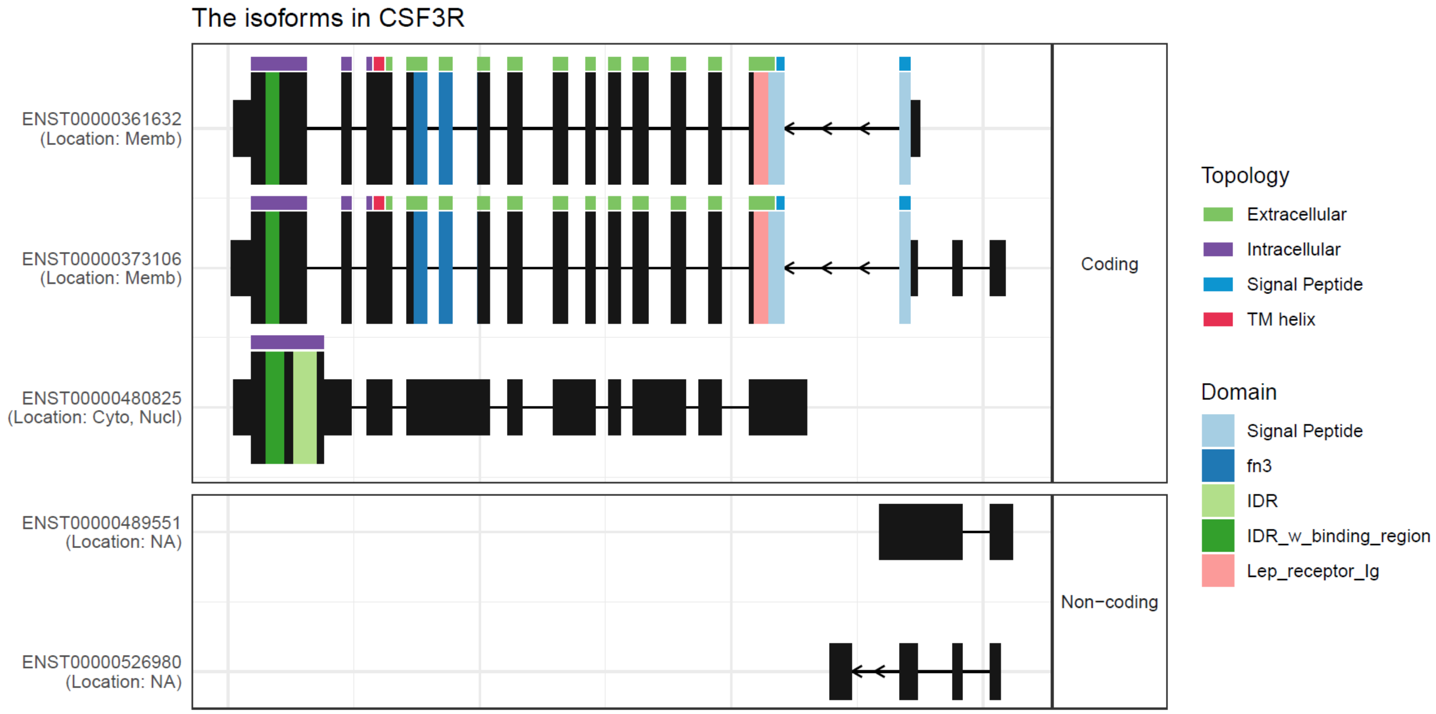

Supplemental Figure 36: CSF3R IsoformSwitchAnalyzeR isoform plot output. ENST00000480825 is annotated as 'retained intron' but here is predicted to be protein coding.

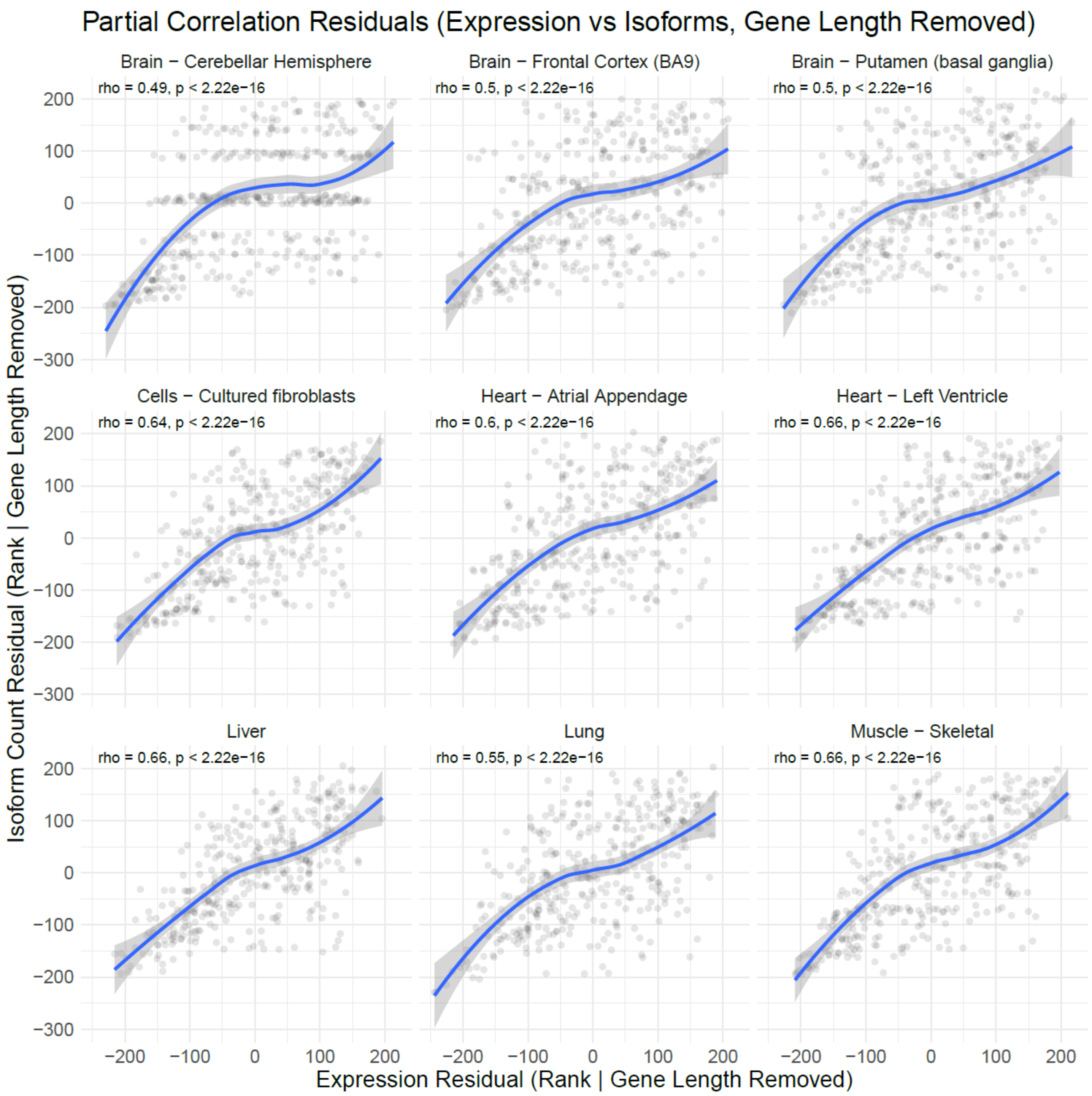

Supplemental Figure 37: Isoform count residual verses gene expression residual. Spearman comparison between number of isoforms and gene expression, controlling for gene length.

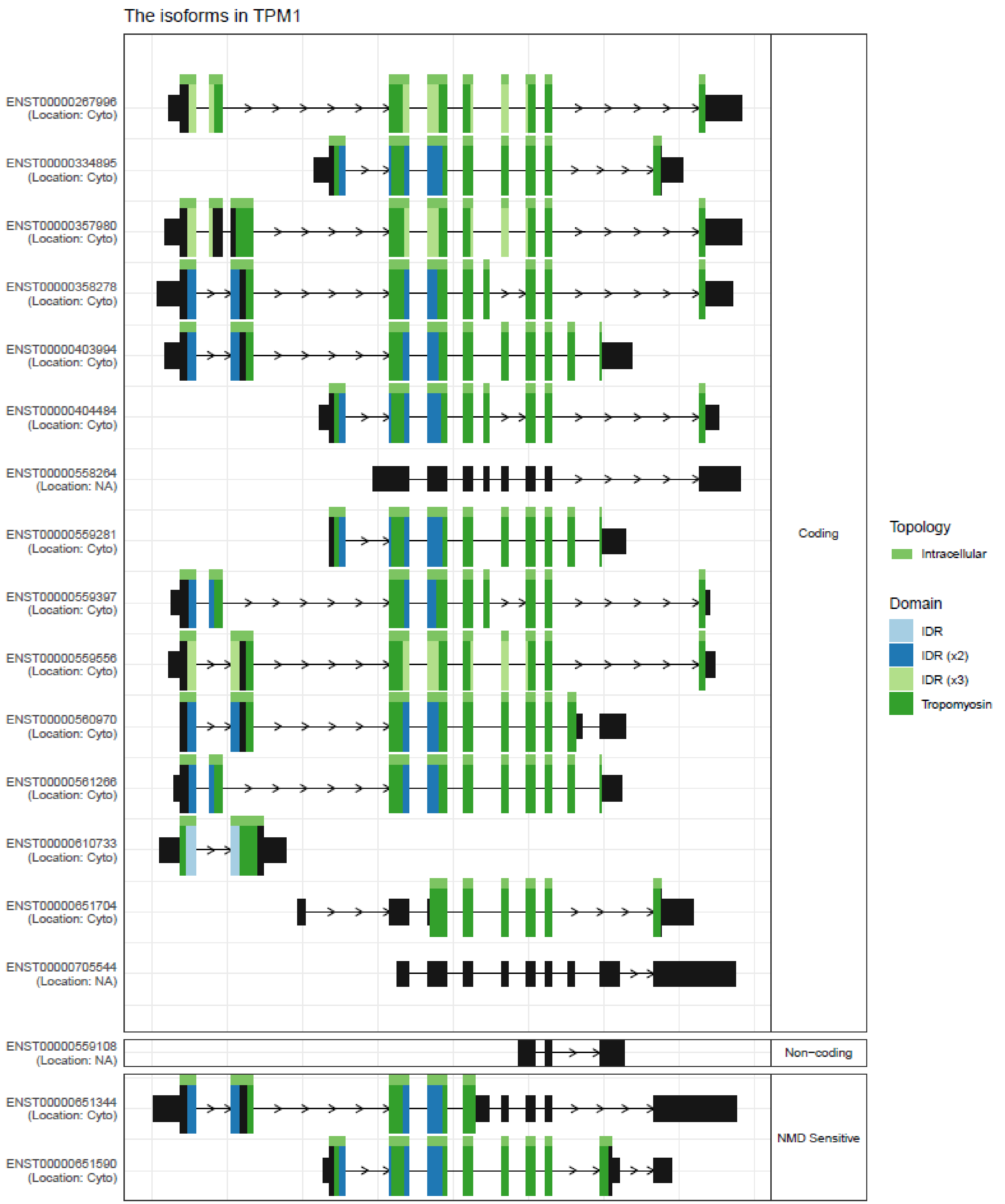

Supplemental Figure 38: TPM1 IsoformSwitchAnalyzeR isoform plot output. ENST00000403994, ENST00000267996, and ENST00000334895 all have different coding regions and our data shows they are preferential in different tissue sets.

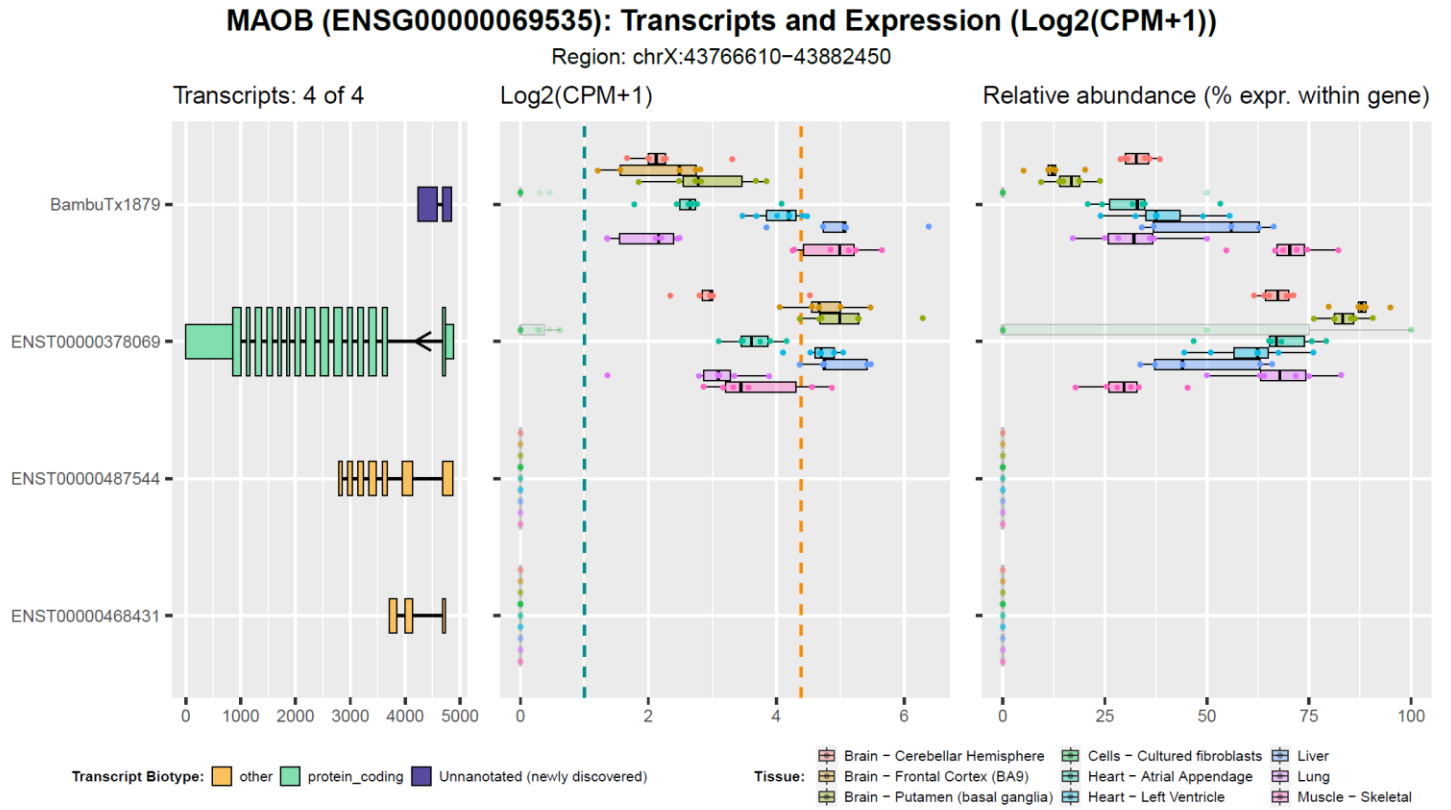

Supplemental Figure 39: MAOB isoforms, expression, and relative expression Isoforms expressed above a median CPM > 1 are considered in our analyses. Faded box-plots represent tissues where the isoform did not pass our unique counts threshold (median unique counts ≥ 1), and therefore are not included in our analyses. Blue dashed line is CPM = 1 and orange dashed line is CPM = 20.

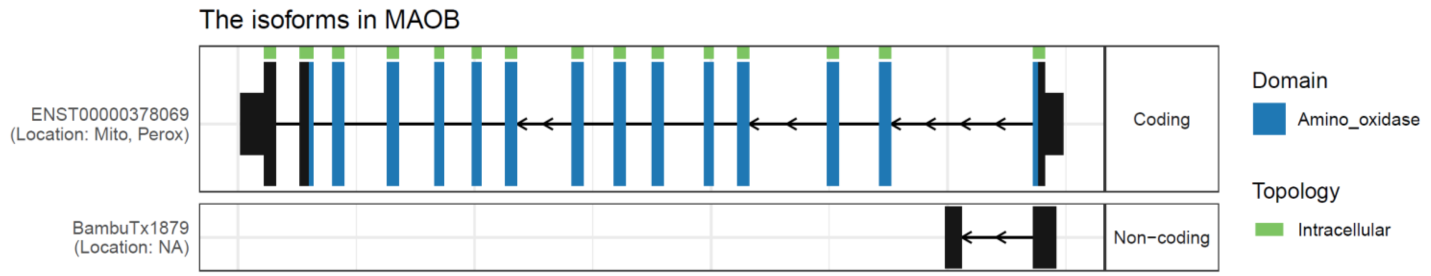

Supplemental Figure 40: MAOB IsoformSwitchAnalyzeR isoform plot output. The newly discovered isoform, BambuTx1879, is predicted to be non-coding.

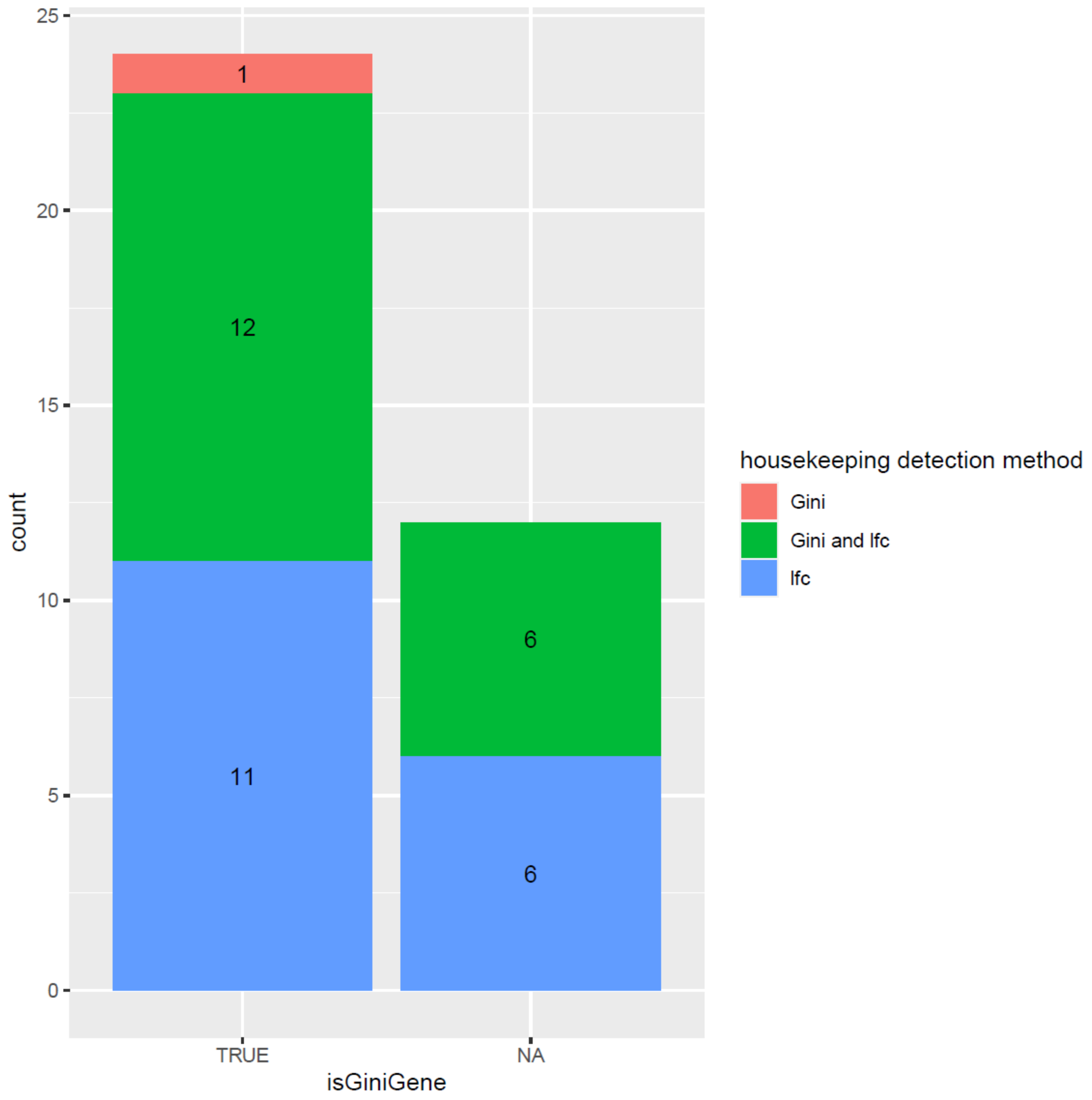

Supplemental Figure 41: Showcasing the detection method we used for determining potential housekeeping isoforms. We used the gini coefficient method (we chose Gini coefficient < 0.3 as our cutoff) as well as our original logfold change method. The X-axis shows whether the isoform we detected came from a gene that the Joshi et. al study identified as a Gini gene.

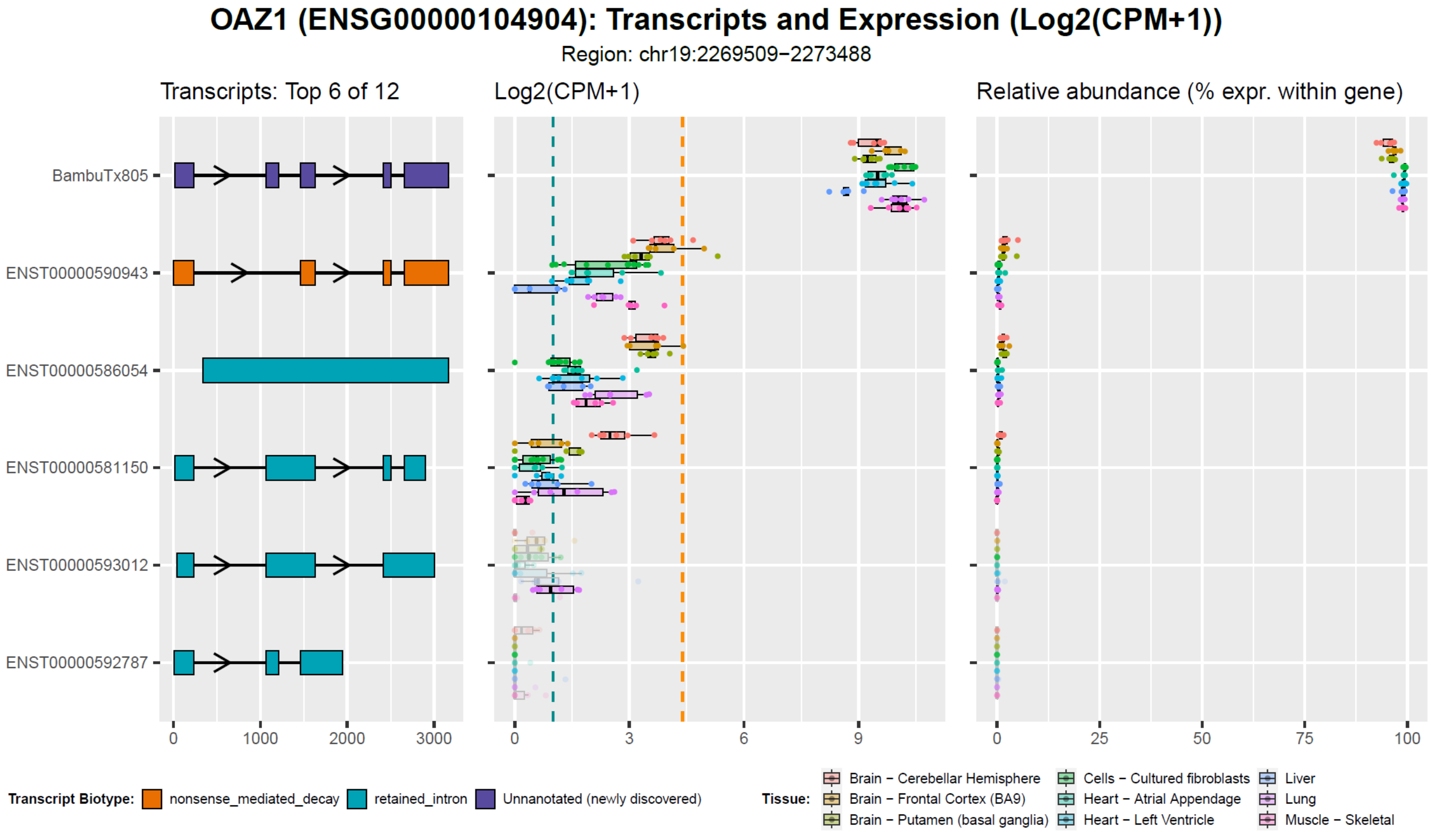

Supplemental Figure 42: OAZ1 isoforms, expression, and relative expression. Isoforms expressed above a median CPM > 1 are considered in our analyses. Faded box-plots represent tissues where the isoform did not pass our unique counts threshold (median unique counts ≥ 1), and therefore are not included in our analyses. Blue dashed line is CPxM = 1 and orange dashed line is CPM = 20.

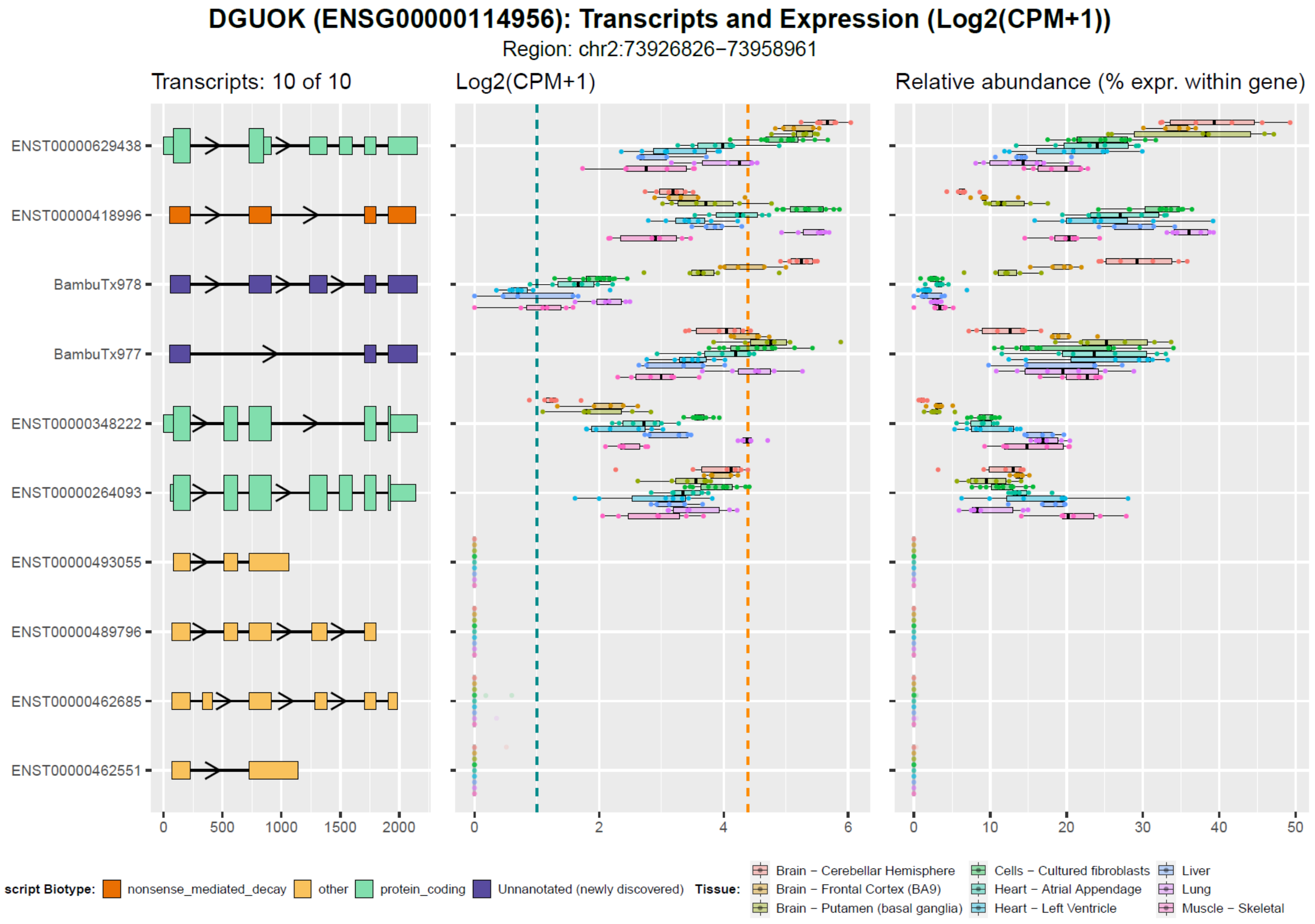

Supplemental Figure 43: DGUOK isoforms, expression, and relative expression. Isoforms expressed above a median CPM > 1 are considered in our analyses. Faded box-plots represent tissues where the isoform did not pass our unique counts threshold (median unique counts ≥ 1), and therefore are not included in our analyses. Blue dashed line is CPM = 1 and orange dashed line is CPM = 20.

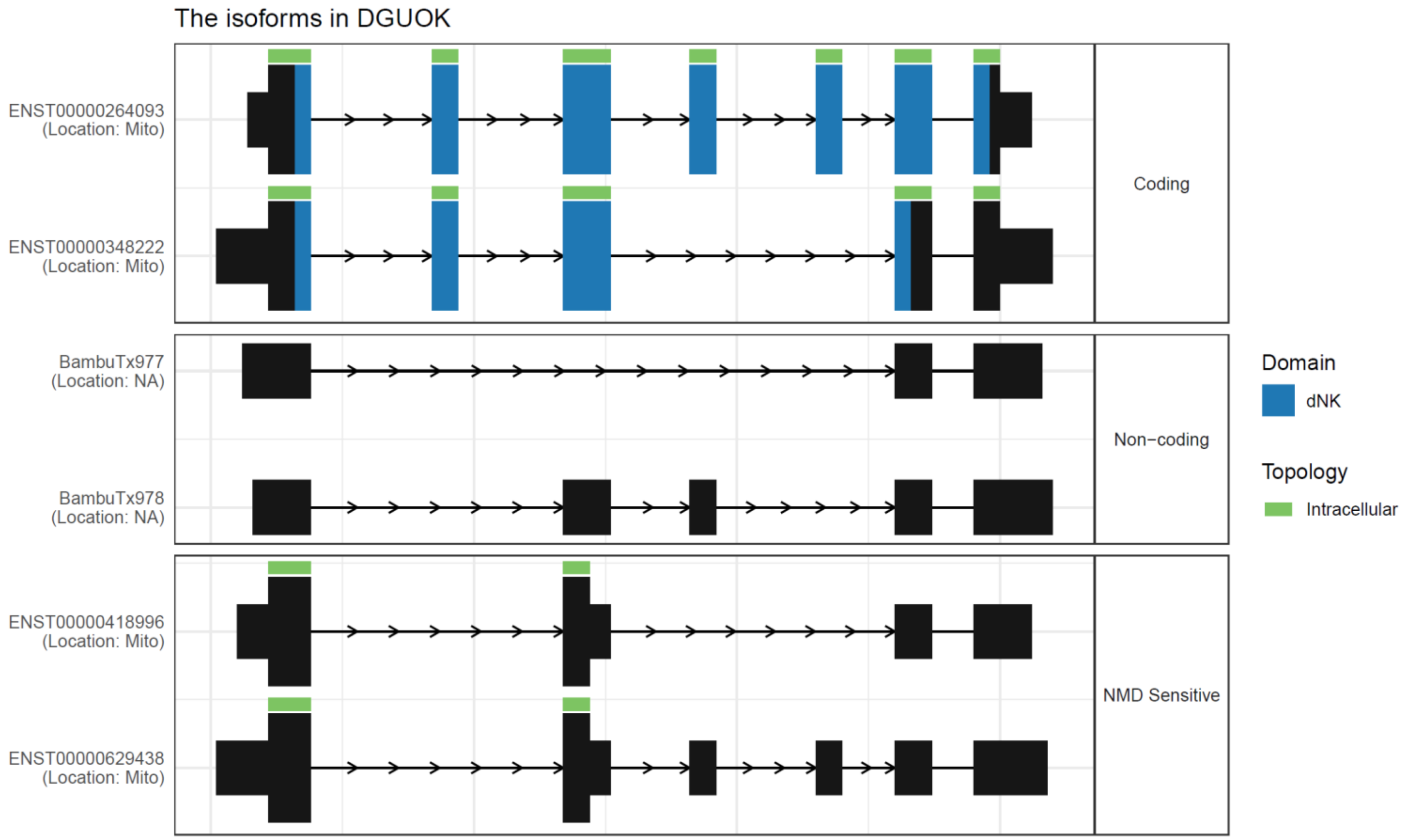

Supplemental Figure 44: DGUOK IsoformSwitchAnalyzeR isoform plot output. Both newly discovered isoforms are predicted to be non-coding.
